# Identification and characterization of CBL and CIPK gene families in canola (*Brassica napus* L*.*)

**DOI:** 10.1186/1471-2229-14-8

**Published:** 2014-01-07

**Authors:** Hanfeng Zhang, Bo Yang, Wu-Zhen Liu, Hongwei Li, Lei Wang, Boya Wang, Min Deng, Wanwan Liang, Michael K Deyholos, Yuan-Qing Jiang

**Affiliations:** 1 State Key Laboratory of Crop Stress Biology for Arid Areas and, College of Life Sciences, Northwest A & F University, Yangling, Shaanxi 712100, China; 2 Department of Biological Sciences, University of Alberta, Edmonton T6G 2E9, Canada

**Keywords:** Abiotic stress, *Brassica napus*, CBL, CIPK

## Abstract

**Background:**

Canola (*Brassica napus* L.) is one of the most important oil-producing crops in China and worldwide. The yield and quality of canola is frequently threatened by environmental stresses including drought, cold and high salinity. Calcium is a ubiquitous intracellular secondary messenger in plants. Calcineurin B-like proteins (CBLs) are Ca^2+^ sensors and regulate a group of Ser/Thr protein kinases called CBL-interacting protein kinases (CIPKs). Although the CBL-CIPK network has been demonstrated to play crucial roles in plant development and responses to various environmental stresses in Arabidopsis, little is known about their function in canola.

**Results:**

In the present study, we identified seven CBL and 23 CIPK genes from canola by database mining and cloning of cDNA sequences of six CBLs and 17 CIPKs. Phylogenetic analysis of CBL and CIPK gene families across a variety of species suggested genome duplication and diversification. The subcellular localization of three BnaCBLs and two BnaCIPKs were determined using green fluorescence protein (GFP) as the reporter. We also demonstrated interactions between six BnaCBLs and 17 BnaCIPKs using yeast two-hybrid assay, and a subset of interactions were further confirmed by bimolecular fluorescence complementation (BiFC). Furthermore, the expression levels of six selected BnaCBL and 12 BnaCIPK genes in response to salt, drought, cold, heat, ABA, methyl viologen (MV) and low potassium were examined by quantitative RT-PCR and these CBL or CIPK genes were found to respond to multiple stimuli, suggesting that the canola CBL-CIPK network may be a point of convergence for several different signaling pathways. We also performed a comparison of interaction patterns and expression profiles of CBL and CIPK in Arabidospsis, canola and rice, to examine the differences between orthologs, highlighting the importance of studying CBL-CIPK in canola as a prerequisite for improvement of this crop.

**Conclusions:**

Our findings indicate that CBL and CIPK family members may form a dynamic complex to respond to different abiotic or hormone signaling. Our comparative analyses of the CBL-CIPK network between canola, Arabidopsis and rice highlight functional differences and the necessity to study CBL-CIPK gene functions in canola. Our data constitute a valuable resource for CBL and CPK genomics.

## Background

Plants have developed complex signal transduction pathways to cope with a fluctuating environment throughout their life cycle. Environmental stresses, such as high salinity, drought, cold and pathogens affect not only plant growth and development but also their yield and food quality. Ca^2+^ is a ubiquitous second messenger that is involved in the signaling of a variety of environmental and developmental stimuli. In response to these stimuli, cells generate transient changes in the intracellular Ca^2+^ concentration and, these changes are sensed and decoded by Ca^2+^ sensors including calmodulins (CaMs), calmodulin-like proteins (CMLs), calcineurin B-like proteins (CBLs) and calcium-dependent protein kinases (CPKs) [1].

CBL and CBL-interacting protein kinase (CIPK) proteins were originally identified in the model plant Arabidopsis [2,3]. CBL proteins show high similarity to the regulatory B subunit of calcineurin (CNB) and neuronal calcium sensor (NCS) proteins in animals and yeast [2]. As a structural basis for Ca^2+^ binding, CBLs contain four EF-hand domains that can bind at most four Ca^2+^ ions [4,5]. CBLs specifically target a group of SNF1 (sucrose non-fermenting 1)-related serine/threonine kinases, group 3 (SnRK3), namely CIPKs, to transduce the perceived calcium signal [2,3]. Commonly, CIPK proteins consist of a conserved N-terminal kinase domain, and a C-terminal regulatory domain, which is separated from the kinase domain by a variable junction domain. Ca^2+^-bound CBLs interact with and activate the catalytic activity of targeting CIPKs through a conserved NAF or FISL motif within the rather divergent C-terminal regulatory domain [6,7]. Moreover, a few CIPKs can also interact with specific members of the 2C-type protein phosphatase (PP2C) through a protein–phosphatase interaction (PPI) domain within the C-terminus of these kinases [8]. So far, bioinformatic analyses of both CBL and CIPK families have identified a total of 10 CBLs and 26 CIPKs in Arabidopsis, and 10 CBLs and 30 CIPKs in rice (*Oryza sativa*), respectively, many of which have been reported to show distinct and selective interactions among these complementary partners [9]. This selectivity allows for a complex interplay of different CBL-CIPK combinations that, in turn, could decode the Ca^2+^ signals from different stimuli through spatiotemporal regulation of downstream signaling cascades. Furthermore, recent evidence demonstrates that phosphorylation of CBL proteins by their interacting CIPKs is required for full activity of CBL-CIPK complexes toward their target proteins [10,11].

Over the past decade, the CBL-CIPK network in Arabidopsis has been demonstrated to play an important role in regulating sodium (Na^+^), potassium (K^+^) and nitrate (NO_3_^-^) transport across the plasma membrane (PM) and/or tonoplast [12-15]. In Arabidopsis, a few members of CBL and CIPK family genes have also been identified to participate in auxin and abscisic acid (ABA) signaling, as well as many other developmental processes in Arabidopsis [9,16]. The first genetically defined CBL-CIPK network was identified in a genetic screen for a salt overly sensitive (SOS) phenotype, and in this pathway CBL4 (SOS3) interacts with CIPK24 (SOS2), and this interaction recruits the kinase to the plasma membrane, where it activates the plasma membrane-localized Na^+^/H^+^ antiporter (SOS1) and vacuolar H^+^-ATPase to promote salt tolerance [17-19]. Later on, it was reported that Arabidopsis CBL10 also interacts with CIPK24. The CBL10-CIPK24 complex is associated with the vacuolar compartments, and functions in protecting shoots from salt stress [20,21]. An Arabidopsis *cipk3* mutant shows ABA hypersensitivity during seed germination and alters the expression pattern of a number of stress marker genes in response to ABA, cold, and high salt [22].

In another forward-genetic screen, mutants sensitive to low potassium showed that loss of *CIPK23* (*LKS1*) function impaired growth under K^+^-limiting conditions, and the interaction of CBL1 or CBL9 with CIPK23 recruits it to the plasma membrane, where it phosphorylates and activates the K^+^ channel AKT1 [15,23], although recent evidence suggests that CBL1 or CBL9 may interact independently of CIPK23 with AKT1 [24]. Moreover, *Arabidopsis* CIPK6 and CIPK16 also interact with AKT1 in a yeast two-hybrid assay and enhance the activity of AKT1 in a CBL-dependent manner in *Xenopus* oocytes [25]. In addition, Arabidopsis CBL4-CIPK6 modulates the activity and plasma membrane (PM) targeting of another K^+^ channel, AKT2, by mediating translocation of AKT2 to the PM in plant cells and enhancing AKT2 activity in oocytes [26]. Besides, AtCIPK6 was shown to be involved in auxin transport and consequently in root development, as well as in the salt-stress response [27]. Another group identified two CIPKs that mediate nitrate nutrition, among which CIPK8 positively regulates the low-affinity phase of the primary nitrate response and CIPK23 can phosphorylate T101 of CHL1 (NRT1.1) to maintain a low-level primary response [12,28]. These studies demonstrate that CBL-CIPK networks play important roles in a variety of environmental stresses.

A similar CBL-CIPK network seems also to exist in rice and maize, as indicated by the presence of ten *CBLs* and 30 *CIPKs* in rice and, 43 *CIPKs* in maize [29,30]. Although the functional significance of most of these CBL-CIPK interactions is not yet clear, they suggest a very complex and dynamic signal transduction network regulated by this calcium sensor and Ser/Thr protein kinase system. Despite extensive studies of the CBL-CIPK network in *Arabidopsis*, so far, there is only one report that suggests *Brassica napus* CBL1-CIPK6 is involved in the plant response to high-salinity, phosphorous deficiency, and ABA signaling [31]. Therefore the identities and roles of the CBL-CIPK network in *B. napus* (canola, oilseed rape) are still largely unknown. How specific CBL-CIPK complexes participate in canola growth and development as well as in response to abiotic and biotic stresses is waiting to be revealed.

Canola-quality oil is defined by low erucic acid and low glucosinolates, and crops that produce canola oil are among the most important oil crops in China and worldwide. Losses from adverse environmental conditions greatly influence canola yield and quality. Understanding the molecular mechanisms of canola responses to abiotic stresses is a prerequisite for improving stress tolerance to meet the increasing demanding for edible oil. It is a promising approach to improve stress tolerance of plants through modulating the expression of key genes in plant breeding. To this end, we initiated the detailed characterization of CBL and CIPK genes in canola (i.e. *B. napus*) and systematically analyzed the interactions between each CBL and CIPK. We also examined the expression patterns of most of the identified CBL and CIPK genes in response to a variety of hormone and stress treatments. Through this work, we will better be able to understand the roles of CBL-CIPK network in canola responses to abiotic stress and hormone stimuli.

## Results and discussion

### The identification and cloning of CBL and CIPK genes in canola

In our previous transcritpomic analysis of canola seed coat development, at least four CIPK genes were shown to be differentially expressed with a two-fold change or more [32]. As the first step to understand the roles of CBL and CIPK genes in canola growth and development, as well as in response to abiotic stresses, we aimed to identify and clone CBL and CIPK genes from canola. Since the sequencing of the *Brassica napus* genome is still incomplete and Arabidopsis is a close relative to *B. napus*, we used 10 Arabidopsis CBL and 26 CIPK genes as queries, and ran BLAST searches of the expressed sequence tag (EST) database of *B. napus* in NCBI (http://www.ncbi.nim.nih.gov/dbEST/index.html). As a result, we identified 80 ESTs representing *CBLs* and 502 ESTs for *CIPKs* (Table 1, Additional file 1), which showed significant similarities, with an E-value lower than 10^-4^. These ESTs were further filtered and assembled to obtain contigs and singlets, which were then reciprocally BLAST searched against Arabidopsis database (http://www.arabidopsis.org/Blast/index.jsp) to identify the putative orthologs in the model plant Arabidopsis. The BnaCBL and BnaCIPK genes were therefore annotated based on the Arabidopsis orthologs with *Bna* standing for *Brassica napus* to differentiate it from *B. nigra* (*Bni*) (Table 1). Afterwards, the amino acids of each contig or singlet were predicted using DNAMAN or DNASTAR program. As a result, we successfully identified ESTs representing 7 BnaCBL and 25 BnaCIPK genes (Additional file 1).

**Table 1:** Canola CBL and CIPK genes identified and their characteristics

**gene**	**GenBank Acc No.**	**EST count**	**Arabidopsis ortholog/AGI No.**	**Rice ortholog/locus**	**No. Amino acids**	**Protein M.W.(kDa)**	**pI**	**No. of EF-hands**	**Palmitoylation sites**	**Myristoylation sites**
*BnaCBL1*	JQ708046	6	*AtCBL1/At4g17615*	*OsCBL1/LOC_Os10g41510*	213	24.6	4.57	4	yes	yes
*BnaCBL2*	JQ708048	11	*AtCBL2/At5g55990*	n.d.	226	25.9	4.79	4	yes	n.d.
*BnaCBL3*	JQ708049	25	*AtCBL3/At4g26570*	*OsCBL3/LOC_Os03g42840*	226	25.8	4.67	4	yes	n.d.
*BnaCBL4*	JQ708050	3	*AtCBL4/At5g24270*	*OsCBL7/LOC_Os02g18880*	221	25.4	4.68	4	yes	yes
*BnaCBL9*	JQ708051	18	*AtCBL9/At5g47100*	n.d.	213	24.4	4.5	4	yes	yes
*BnaCBL10*	JQ708047	16	*AtCBL10/At4g33000*	*OsCBL10/LOC_Os01g51420*	249	28.8	4.87	4	yes	n.d.
*BnaCIPK1*	JQ708052	28	*AtCIPK1/At3g17510*	*OsCIPK21/LOC_Os07g44290*	444	50	6.3	0	yes	n.d.
*BnaCIPK3*	JQ708061	13	*AtCIPK3/At2g26980*	*OsCIPK32/LOC_Os12g03810*	440	*50.3*	6.69	0	n.d.	n.d.
*BnaCIPK5*	JQ708062	11	*AtCIPK5/At5g10930*	*OsCIPK16/LOC_Os09g25090*	431	49.4	6.88	0	yes	n.d.
*BnaCIPK6*	JQ708063	199	*AtCIPK6/At4g30960*	*OsCIPK5/LOC_Os01g10890*	437	49	8.79	0	yes	n.d.
*BnaCIPK7*	JQ708064	13	*AtCIPK7/At3g23000*	*OsCIPK4/LOC_Os12g41090*	414	46.5	9.33	0	yes	n.d.
*BnaCIPK8*	JQ708065	26	*AtCIPK8/At4g24400*	*OsCIPK8/LOC_Os01g35184*	451	51	8.32	0	n.d.	n.d.
*BnaCIPK9*	JQ708066	28	*AtCIPK9/At1g01140*	*OsCIPK9/LOC_Os03g03510*	447	50.4	7.93	0	yes	n.d.
*BnaCIPK10*	JQ708053	14	*AtCIPK10/At5g58380*	*OsCIPK2/LOC_Os07g48100*	463	52.9	8.13	0	yes	n.d.
*BnaCIPK11*	JQ708054	30	*AtCIPK11/At2g30360*	n.d.	443	49.8	8.26	0	yes	n.d.
*BnaCIPK12*	JQ708055	35	*AtCIPK12/At4g18700*	*OsCIPK12/LOC_Os01g55450*	491	55	7.81	0	yes	n.d.
*BnaCIPK14*	KC414027	9	*AtCIPK14/At5g01820*	*OsCIPK19/LOC_Os05g43840*	431	48.6	9.06	0	yes	n.d.
*BnaCIPK15*	JQ708056	9	*AtCIPK15/At5g01810*	n.d.	423	48	8.61	0	n.d.	n.d.
*BnaCIPK17*	JQ708057	6	*AtCIPK17/At1g48260*	n.d.	427	47.7	7.68	0	yes	n.d.
*BnaCIPK23*	JQ708058	15	*AtCIPK23/At1g30270*	*OsCIPK23/LOC_Os07g05620*	482	53.4	9.25	0	yes	n.d.
*BnaCIPK24*	JQ708059	11	*AtCIPK24/At5g35410*	*OsCIPK24/LOC_Os06g40370*	453	51.6	9.2	0	n.d.	n.d.
*BnaCIPK25*	KC414028	18	*AtCIPK25/At5g25110*	n.d.	455	51.6	8.54	0	yes	n.d.
*BnaCIPK26*	JQ708060	18	*AtCIPK26/At5g21326*	*OsCIPK32/LOC_Os12g03810*	441	50	8.11	0	n.d.	n.d.

The calcineurin B-like proteins (CBLs) and CBL-interacting protein kinases (CIPKs) genes of canola (*Brassica napus* L.) were identified through mining the expression sequence tags (ESTs), with cDNA sequences cloned through RACE-PCR and deposited in the GenBank. The nomenclature of BnaCBL and CIPK followed that of Arabidospis through a comparison in InParanoid7 (http://inparanoid.sbc.su.se/cgi-bin/index.cgi). The molecular weight (M.W.) and isoelectric point (pI) of the encoded proteins were predicted by DNAMAN software. The number of EF-hands was predicted by PROSITE scan. The palmitoylation sites were predicted by CSS-Palm 3.0 and myristoylation sites by Myristoylator. n.d., not detected.

We noted that among all the *BnaCBLs* annotated, *BnaCBL3* had the largest number (25) of ESTs, followed by *BnaCBL9* with a total of 18 ESTs and, *BnaCBL10* with 16 ESTs while *BnaCBL*6 has only one EST (Additional file 1). Among the 25 *BnaCIPKs* annotated, *BnaCIPK6* had the largest number (199) of ESTs, followed by *BnaCIPK12* with a total of 35 ESTs and *BnaCIPK11* with 30 ESTs, while *BnaCIPK13, 18, 19* and *20* had only one EST each (Additional file 1). To facilitate subsequent phylogenetic, GFP fusion, and yeast two-hybrid analyses, we designed primers based on the identified ESTs for each of the *BnaCBL* and *BnaCIPK* genes to obtain full length cDNA sequences, employing RT-PCR together with RACE. As a result, we succeeded in cloning the cDNA sequences of six of these seven *BnaCBL* genes, and 17 of these 25 *BnaCIPK* genes (Table 1). We were also able to identify putative orthologs of these *BnaCBL and BnaCIPK* genes in both *Arabidopsis* and rice using the program InParanoid (http://inparanoid.sbc.su.se/cgi-bin/index.cgi) (Table 1).

**Figure 1:**
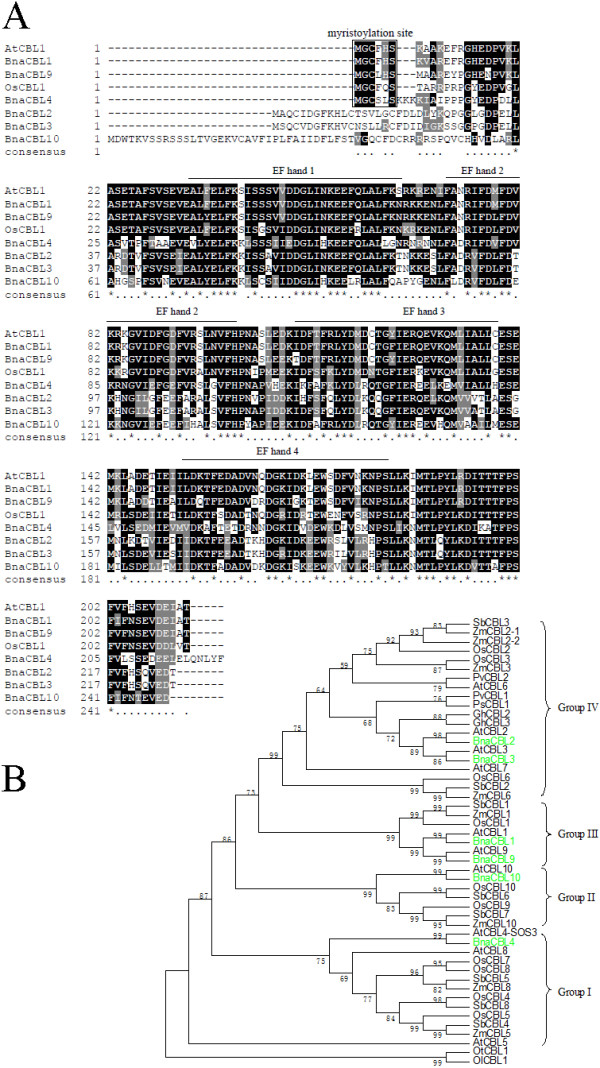
**Domain analysis and phylogenetic relationship of canola CBL proteins with CBLs from other species. (A)** Multiple sequence alignment was performed using the ClustalX1.83 and illustrated by BOXSHADE (http://www.ch.embnet.org/software/BOX_form.html). Identical amino acids are shaded in black, and similar amino acids are shaded in gray. The four EF-hand motifs are indicated by overbars. The myristoylation site is in the rectangle. **(B)** Phylogenetic relationship of canola CBL proteins with CBLs from other species. Protein sequences were aligned using ClustalX (v1.83) and a maximum parsimony (MP) bootstrap consensus tree was drawn using MEGA5.1. The CBLs can be clustered into four major groups (I-IV). The percentage of replicate trees is shown on the branches and it is calculated in the bootstrap test (1000 replicates) for the associated taxa being clustered together. At, *Arabidopsis thaliana*; Bna, *Brassica napus*; Gh, *Gossypium hirsutum*; Ol, *Ostreococcus lucimarinus*; Os, *Oryza sativa*; ; Ot, *Ostreococcus tauri*; Ps*, Psium sativum;* Pv, *Phaseolus vulgaris;* Sb, *Sorghum bicolor*; Zm, *Zea maize*.

The deduced amino acid sequences of six BnaCBL genes demonstrated great conservation in size (Figure 1A). The molecular weight of the predicted proteins ranged from 24.4 to 28.8 kDa. We also identified that the amino acid sequence identity of different BnaCBLs ranging from 46.7% to 90.7% (65.9-96.2% similarity, Additional file 2A), with highly conserved C-terminal regions flanking the EF-hand domains.

As in AtCBL1 and OsCBL1, four EF-hand motifs in the six BnaCBLs could be identified (Figure 1A). As Ca^2+^ sensors, CBL proteins are able to bind Ca^2+^ ions through the EF-hand motifs [4,5,33,34]. Indeed, each of the six BnaCBL proteins was found to contain four EF-hand motifs (Table 1), although some of the EF-hand motifs were not canonical, as compared to calmodulin (CaM) and calmodulin-like (CML) proteins from Arabidopsis, rice, *C. elegans*, yeast, fruit fly, zebrafish, mouse and human (Additional file 3). Each EF-hand consisted of a loop of 12 amino acids flanked by two helices (E helix and F helix). The E helix generally starts with a glutamate (E) and, F helix a leucine (L), phenylalanine (F) or isoleucine (I); both the E and F helices flanking the Ca^2+^-binding loop are generally each 9 amino acids long (Additional file 3). However, the EF1 loop of BnaCBLs contains an insertion of two amino acid residues between position 1 and position 3. Previous studies also demonstrated that the CBL–CIPK interaction may also affect the Ca^2+^-binding capacity of CBLs, as observed with AtCBL2/AtCIPK14 and AtCBL4/AtCIPK24 [5,33,34].

In addition, we used the Motif Scan program (http://myhits.isb-sib.ch/cgi-bin/motif_scan) and other programs to search other possible motifs that could be functionally important in fulfilling their roles. Myristoylation is an irreversible protein modification in which myristate, a 14-carbon saturated fatty acid, is covalently attached through an amide bond to an N-terminal glycine residue in a co-translational process [35]. Our analysis revealed that three BnaCBL proteins (BnaCBL1, -4 and -9) started with a conserved N-myristoylation motif (MGXXXS/T) that might function in membrane targeting of the CBL-CIPK complex, whereas the other three BnaCBL proteins did not have such a motif (Table 1, Figure 1A). This structural feature was also observed within rice CBL proteins [30,36]. In contrast, palmitoylation (more correctly known as S-acylation) is the reversible addition of fatty acids to proteins, which increases their membrane affinity. S-acylated proteins play a wide variety of roles in plants and affect calcium signalling, K^+^ movement, stress and hormone signaling and many other processes [37]. Both myristoylation and palmitoylation are involved in the membrane association of various proteins, such as G protein alpha-subunits, src tyrosine kinases and nitric oxide synthase in animals, however, little is known about roles of them in plants until recent years [38-41]. Interestingly, we found that all the six BnaCBL protein bear typical palmitoylation sites at the N-terminal, although the meaning awaits further investigation. A recent study demonstrated that palmitoylation of the Arabidopsis CBL2 associates it with the vacuolar membrane, which is essential for proper ABA responses [38]. Moreover, we observed in the canola BnaCBL1, -4 and -9 proteins a very conserved cysteine (C) residue following the glycine (G) as a structural feature, suggesting a potential dual N-terminal lipid modification of these three canola CBLs. Similar observations were also made with Arabidopsis CBL1, -4 and -9 and it was demonstrated that dual fatty acyl modification determines the localization and plasma membrane targeting of CBL1-CIPK1 signaling complexes in Arabidopsis [39].

As for BnaCIPK proteins, the molecular weight of the predicted proteins ranged from 46.5 to 55.0 kDa (Table 1). We also found that the amino acid sequence identity of different BnaCIPKs ranged from 37% to 76.2% (58.1-90% similarity, Additional file 2B). The deduced amino acid sequences of 17 BnaCIPK genes demonstrated great conservation in size (Additional file 4A). As in CIPKs from Arabidopsis, all BnaCIPKs consisted of a conserved N-terminal kinase domain, followed by a variable junction domain and a C-terminal regulatory domain, (Additional file 4A). Within the rather divergent regulatory domain, a conserved NAF or FISL motif was identified, which has been reported to be required for mediating CBL interaction [6,7]. MEME analysis showed that the amino acid residues at the 5^th^, 6^th^, 7^th^, 10^th^, 13^th^, 18^th^, 21th, 22th sites of the NAF/FSIL motif are rather conserved, while others not (Additional file 4B). Even at the conserved sites of this motif, variations also existed in canola CIPK proteins. For example, we noted that the fifth amino acid residue of the NAF motif in BnaCIPK7 was T (threonine) instead of N (asparagines), and the 21^st^ amino acid residue of BnaCIPK1 was F (phenylalanine) instead of L (leucine) (Additional file 4A). Whether these differences in amino acid residues of a CIPK have any influence on their ability to interact with CBLs needs to be investigated. Similar changes in other plant CIPKs were also observed, including AtCIPK4, AtCIPK7, OsCIPK29 and *Phaseolus vulgaris* (Pv) CIPK2 (data not shown). Sequence analysis also revealed a protein–phosphatase interaction (PPI) motif within the C-terminus of these kinases (Additional file 4A), which is assumed to mediate the CIPK interaction with type 2C protein phosphatases (PP2Cs) [8]. However, the amino acids within this PPI motif showed very limited conservation, except at sites 7, 8, 10, 17, 20, and 22, which are amino acids arginine (R), phenylalanine (F), serine (S), isoleucine (I), lysine (K) and glutamate (E), respectively (Additional file 4C).

### Phylogenetic analysis of BnaCBL and BnaCIPK proteins

To better understand the evolutionary history of both CBL and CIPK families, we also identified and retrieved CBL and CIPK genes from a variety of species using an HMM-based search. The search space was composed primarily of fully-sequenced genomes from the major land plant lineages including the bryophyte *Physcomitrella patens* (Pp), the lycophyte *Selaginella moellendorffii* (Sm), and several mono- and eudicotyledonous angiosperms, i.e. the eudicots *Arabidopsis thaliana* (At), *Medicago truncata* (Mt)*, Pisum sativum* (Ps) *Solanum lycopersicum* (Sl), and *Glycine max*[42], and the monocots *Oryza sativa* (Os), *Sorghum bicolor* (Sb), *Brachypodium distachyon* (Bd), and *Zea mays* (Zm) (Additional files 5 and 6) . To differentiate CBL genes from calmodulin (CaM) or calcium-dependent protein kinase (CDPK/CPK) genes, whose protein sequences also contain EF-hand motifs, and to separate CIPK genes from SnRK1s, SnRK2s or other types of kinase genes, we ran a reciprocal BLASTP search of these putative CBLs or CIPKs from other species against databases of Arabidopsis (http://www.arabidopsis.org, TAIR10) and rice (http://rice.plantbiology.msu.edu/index.shtml, release 7), and also analyzed the domain characteristics. Only CBL or CIPK genes were kept to reconstruct the phylogenetic trees. To trace the origins of both CBL and CIPK gene families, we also performed an HMM-based search of any possible CBL or CIPK genes from a marine green alga *Ostreococcus tauri* (*Ot*), which is the world’s smallest free-living eukaryote known to date [43] and also from a pico-eukaryotic (bacterial-sized) prasinophyte green alga *Ostreococcus lucimarinus*, which has one of the highest gene densities known in eukaryotes [44]. As a result, we identified one CBL and one CIPK gene from each of these two algal species (Additional files 5 and 6), which were also reported recently [45].

Although previous studies reported 30 CIPK genes in rice (subsp. *japonica*) [30,46], our search indentified an additional four putative OsCIPK genes, *LOC_Os07g48760*, *LOC_Os12g03810*, *LOC_Os11g03970* and *LOC_Os02g08140*. We named these *OsCIPK31 to OsCIPK34*, respectively (Additional file 6). The protein sequences of OsCIPK31, -32, -33 and -34 had complete kinase and regulatory domains, with NAF/FISL motif in the C-terminal regions, which are characteristics of CIPKs (Additional file 7). Similarity comparisons showed the encoded proteins of *OsCIPK31*, *-32*, *-33* and -*34* had high similarity to AtCIPK3 (63.2%), -3 (75.6%), -3(72.8%) and -21(53.8%), respectively, among the 26 AtCIPK proteins compared (Additional file 2)*.* On the other hand, using our criteria, we found that the previously identified rice OsCIPK8 and OsCIPK21 were atypical CIPKs, since OsCIPK21 had an incomplete kinase domain and OsCIPK8 had lost the region containing the FISL/NAF motif, which is pointed out previously [46] (Additional file 7). Moreover, we found that the locus number of *OsCIPK22* (*Os05g26870*) may have been wrongly annotated [46], since the encoded protein of Os05g26870 does not have any characteristics of a CIPK protein and does not show any significant similarity to any Arabidopsis protein either. We assigned a new locus number *LOC_Os05g26940* to *OsCIPK22* gene (Additional file 6). We also found that OsCIPK14 and OsCIPK15 had almost identical protein sequences (98.2% identity or 98.4% similarity, Additional file 3), except that there were three substitutions and a five amino acid insertions at the C-terminal end of OsCIPK14 (Additional file 7). As for OsCIPK32 and OsCIPK33, their protein sequences also showed 95.8% identity or 96% similarity (Additional file 3), except that there were three substitutions and a 16 amino acid insertion in the C-terminal region of OsCIPK33 (Additional file 7). The high identity existing between *OsCIPK14* and -*15* as well as *OsCIPK32* and -*33* suggests genome duplication of rice CIPK genes during evolution. According to our criteria, we excluded OsCIPK8 and -21 from the phylogenetic analysis of CIPK family as described below.

Similarly, we identified CBL and CIPK genes from other plant species, for instance, *Brachypodium distachyon* (eight different *CBLs* and 30 distinct *CIPKs*), tomato (*Solanum lycopersicum*, 13 *CBLs* and 23 *CIPKs*), *Medicago truncatula* (11 *CBLs* and 15 *CIPKs*), a diploid cotton (*Gossypium raimondii*, 13 *CBLs* and 38 *CIPKs*), apple (*Malus domestica*, 17 *CBLs* and 42 *CIPKs*), and the moss *Physcomitrella patens* (five *CBLs* and seven *CIPKs*) as listed in Additional files 5 and 6. The identification of multiple members of CBL and CIPK gene families in all the analyzed plant species suggests that CBL and CIPK proteins very likely form a complex signaling network to respond to developmental and environmental stimuli [9,16].

It was observed that the size of the canola CBL or CIPK gene family is comparable to that in either Arabidopsis or rice, although the exact numbers await to be determined after sequencing of canola genome is finished. On the other hand, only five CBL and seven CIPK genes were identified from the lower land plants *P. patens*, and a sole *CBL* and *CIPK* were identified in both *O. tauri* and *O. lucimarinus* (Additional files 5 and 6), as was recently described elsewhere [9,45]. This indicates an expansion of these two gene families after the divergence of flowering plants from the remainder of the tracheophyte lineage. Furthermore, comparing the numbers of CBL and CIPK genes between lower and higher organisms indicates an obvious expansion of both gene families during the long history of evolution.

**Figure 2:**
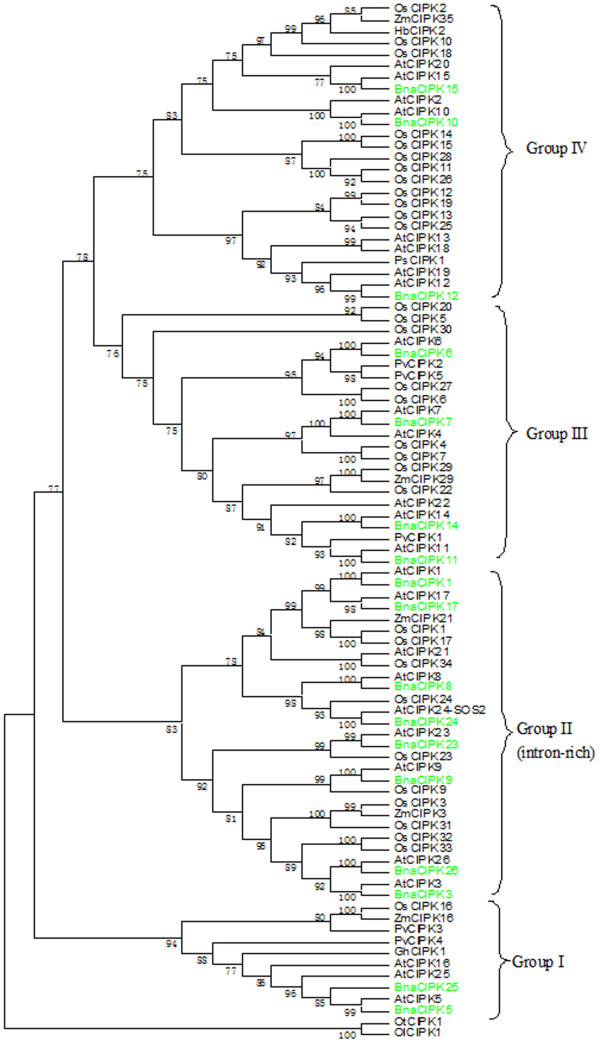
**Phylogenetic relationships of canola CIPK proteins were compared with those from other species.** The phylogenetic tree was based on the amino acid sequences of CIPK proteins from representative species. Protein sequences were aligned using ClustalX (v1.83) and a maximum parsimony tree was inferred using MEGA5.1. The percentage of replicate trees is shown on the branches and it is calculated in the bootstrap test (1000 replicates) for the associated taxa being clustered together. At, *Arabidopsis thaliana*; Bna, *Brassica napus*; Gh, *Gossypium hirsutum*; Os, *Oryza sativa*; Ps*, Pisum sativum;* Pv, *Phaseolus vulgaris;* Hb, *Hordeum brevisubulatum*; Zm, *Zea maize*, Ol, *Ostreococcus lucimarinus*; Ot, *Ostreococcus tauri.*

The amino acid sequences of BnaCBLs or BnaCIPKs together with CBLs or CIPKs from other species were aligned separately and a bootstrapped consensus maximum parsimony (MP) tree was inferred for CBL (Figure 1B, Additional file 8) and CIPK (Figure 2, Additional file 9) gene families, respectively. As shown by the tree’s topology, the CBL proteins from various species could be divided into four major groups (I to IV), each supported by highly significant bootstrap values. The six canola CBLs were distributed in each of the four groups, with BnaCBL1 and -9 belonging to Group III, BnaCBL2 and -3 Group IV, BnaCBL4 Group I, and BnaCBL10 Group II (Figure 1B, Additional file 8). It was also noted that the six BnaCBL members were always clustered closely with AtCBL orthologs, which indicates that the relationship of the two species in Brassicae family are evolutionarily more closely than that of other dicots or monocots we investigated here.

To gain insights in the evolutionary history of the CIPK family in plants, we used the 17 canola CIPK sequences as well as those identified and retrieved from various plant species (Figure 2, Additional file 9). The deduced amino acid sequences of a single CIPK gene from *O. tauri* (*Ot*) and *O. lucimarinus* (*Ol*) each were used to root a bootstrapped consensus tree. The resulting dendrogram showed all CIPKs could be classified into four distinct groups (I to IV), based on their sequence similarity, which was further supported by the high bootstrap values (Figures 2, Additional file 9). We found that the identified 17 BnaCIPKs could be assigned unambiguously to four separate groups, together with 20 AtCIPKs and 32 typical OsCIPKs. Group I included BnaCIPK5 and -25, Group II included BnaCIPK1, -3, -8, -9, -17, -23, -24 and -26, Group III included BnaCIPK6, -7, -11 and -14 and, Group IV included BnaCIPK10, -12 and -15. As expected, all of the 17 BnaCIPKs were clustered more closely with Arabidopsis than those of other species. Another interesting feature is that the intron-rich CIPKs coincidently formed a monophyletic Group I, which was separated from the other groups that contained intron-poor CIPKs, which is consistent with previous reports [30,46].

From our phylogenetic analysis, multiple alignment and domain analysis of BnaCBLs and BnaCIPKs in canola, we concluded some of the CBL and CIPK family members may be conserved among monocots or dicots, while others were lost after the divergence of the monocots and dicots. For instance, we found that CBL2 and CBL9 are presented in the dicots Arabidopsis and canola, but absent in the monocot rice, based on a stringent orthologous analysis (Table 1). The phylogenetic analysis together with the domain motif analysis presented here will facilitate the functional annotation and study of canola *CBLs* and *CIPKs*.

### Subcellular localization of selected BnaCBL and BnaCIPK proteins

**Figure 3:**
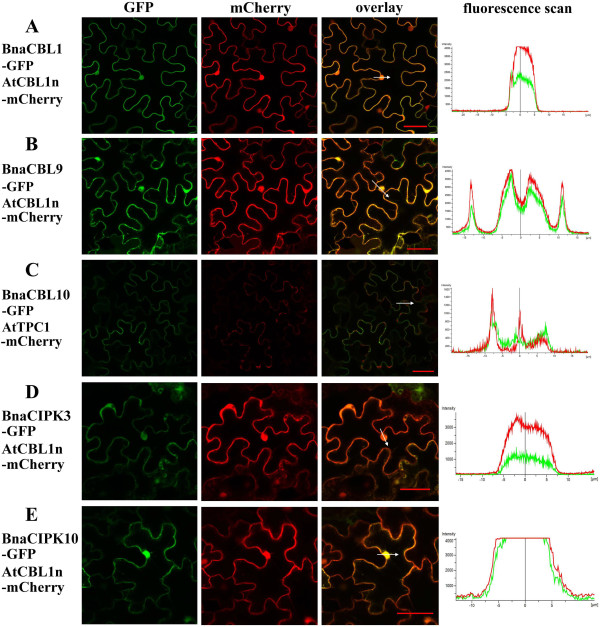
**Subcellular localization of selected BnaCBL- and BnaCIPK-GFP fusion proteins in *N. benthamiana *leaf cells. (A)** Co-localization analysis of BnaCBL1-GFP (green) and AtCBL1n-mCherry (red). **(B)** Co-localization analysis of BnaCBL9-GFP (green) and AtCBL1n-mCherry (red). **(C)** Co-localization analysis of BnaCBL10-GFP (green) and AtTPC1-mCherry (red). **(D)**-**(E)** Co-localization analysis of BnaCIPK3-GFP and BnaCIPK10-GFP with AtCBL1n-mCherry fusion protein, respectively. A white arrow marks the region and direction in which the distribution of fluorescence intensities was scanned. In each case, the extreme left panel is GFP fluorescence, the middle mCherry field and the right represents an overlay of the two images. Bar = 50 μm.

The subcellular localization of a protein may provide evidence of its function. So far, the subcellular localization of several Arabidopsis CBL and CIPK proteins has been determined [20,23,39,40], however the subcellular localization of many other CBL or CIPK proteins from Arabidopsis, rice and a few other species has not been reported. We selected three *BnaCBLs* and two *BnaCIPKs* for fusion to the *Green Fluorescent Protein* (*GFP*) reporter gene. We first fused the coding regions of *BnaCBL1,*-9 and -10 as well as, *BnaCIPK3* and -*10* to the 5′ of *GFP* in a binary vector. To help to determine the subcellular localization of the fusion proteins, two intracellular localization markers fused to mCherry fluorescent protein were used. One is CBL1n, which harbors only the N-terminal of Arabidopsis CBL1 protein, which has been shown to localize at the plasma membrane [39]. The other is the tonoplast marker, two-pore channel 1 (TPC1), from Arabidopsis [47]. An *Agrobacterium tumefaciens* suspension culture transformed with each of these *BnaCBL-* or *BnaCIPK-GFP* constructs, together with the corresponding marker construct, was co-infiltrated into leaves of *N. benthamiana*, and the GFP and mCherry signals were examined two days later. As shown in Figure 3, the BnaCBL1-GFP fusion protein emitted a green fluorescent signal in nuclei and plasma membranes of epidermal cells of leaves and, BnaCBL9-GFP appeared in the cytoplasm and nuclei (Figure 3A, B), whereas BnaCBL10-GFP was localized in the vacuolar membrane or tonoplast (Figure 3C). BnaCIPK3-GFP and BnaCIPK10-GFP were also localized in both cytoplasm and nuclei (Figure 3D, E). As a control, we tested the subcellular localization of GFP protein alone in the leaf cells of *N. benthamiana* and observed that the signal was spread all over the cytoplasm of the leaf epidermal cells as well as in the nuclei (data not shown).

Previous studies on the subcellular localizations of Arabidopsis CBL and CIPK proteins demonstrated that both AtCBL1 and AtCBL9 localize to the plasma membrane [23,39,40], although in approximately 20% of the analyzed cells, the authors also observed traces of CBL9-GFP localization in the nucleus [40]. It was also shown that AtCBL10 is localized at the vacuolar membrane [20,40].

Comparing subcellular localization of the orthologs of CBL1, -9 and -10 between Arabidopsis and canola, we concluded that the patterns were generally conserved, except for the nuclear localization of BnaCBL1. As for CIPKs, a previous study displayed a similar localization pattern that included strong fluorescence in the cytoplasm and nucleus for Arabidopsis CIPK3 and CIPK10 proteins [40], with is completely in agreement with our assay with canola orthologs.

To further analyze the subcellular locations of the respective canola CBL and CIPK proteins, we used four different online programs: WoLF PSORT, CELLO v2.5, TargetP and ESLPred, to predict the subcellular localization of the six BnaCBLs and 17 BnaCIPKs (Additional file 10). It was obvious that differences existed between the predictions from these four programs, although most predictions pointed to cytoplasmic and/or nuclear localization of BnaCBL and BnaCIPK proteins. At the same time, we used TMHMM (http://www.cbs.dtu.dk/services/TMHMM-2.0/) to predict transmembrane helices (TMHs) of these proteins and only one TMH was predicted with BnaCBL10 protein (Additional file 10). Comparing the prediction results to *in vivo* assay using GFP as presented above suggests that it is important to examine the subcellular locations of CBL or CIPK *in planta*.

### Interaction patterns between BnaCBL and BnaCIPK proteins

**Figure 4:**
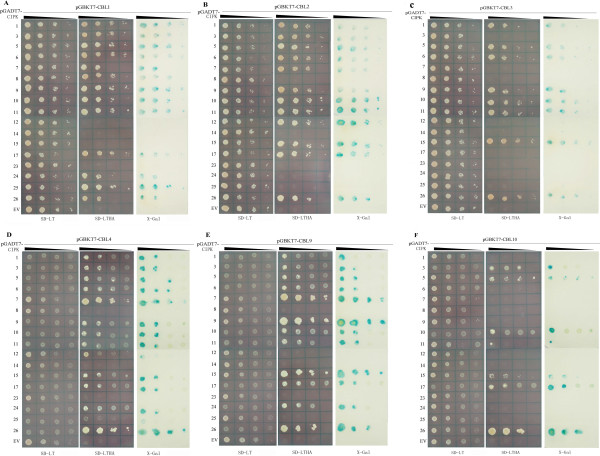
**Yeast two-hybrid analysis of interactions between canola CBL and CIPK proteins.** The yeast cells of strain AH109 harboring the indicated plasmid combinations were grown on either the nonselective (SD-LT) or selective (SD-LTHA) media, followed by β-galactosidase assay (X-Gal staining). Decreasing cell densities in the dilution series are illustrated by narrowing triangles. The **A-F** panels represent the titration assay results of interactions between BnaCBL1, -2, -3, -4, -9, -10 and 17 BnaCIPKs, respectively. The last row in each panel is the control test using pGADT7 empty vector (EV).

Most of the CBL and CIPK genes that have been characterized to date are from Arabidopsis. It has been reported that different CBL proteins interact with different CIPK proteins and the specificity of this interaction determines the network outcome [9,16]. To investigate the interaction preferences of CBL proteins with CIPKs of canola, we used a yeast two-hybrid system*.* Six *BnaCBLs* and 17 *BnaCIPKs* were cloned in-frame into pGBKT7 and pGADT7 vectors, respectively. After transformation into the yeast strain AH109, interactions were detected by growth on nonselective (SD-Leucine-Tryptophan, SD-LT), selecting media lacking histidine, leucine, tryptophan supplemented with 5 mM of 3-aminotriazole (3-AT) (SD-LTH + 3-AT), and selecting media lacking histidine, leucine, tryptophan, adenine hemisulfate (SD-LTHA). We observed that the colony growth on the two types of selective media (SD-LTH + 3-AT, SD-LTHA) was slightly different and only those yeast colonies that grew on the most stringent media SD-LTHA were scored as having interacting protein partners. The authenticity and strength of the interactions were further examined by a titration assay and X-gal staining (Figure 4).

As shown in Figure 4, BnaCBL1 interacted significantly with a subset of 13 CIPKs, among which it showed strong interactions with BnaCIPK1, -3, -5, -6, -9, -10, -11, -17, -25 but weak interactions with BnaCIPK7, -8, -24 and -26 (Figure 4A). BnaCBL2 exhibited a significant interaction with 12 CIPKs, which were BnaCIPK1, -3, -5, -6, -7, -9, -10, -11, -12, -15, -17 and -26 (Figure 4B). BnaCBL3 exhibited a strong interaction with only eight of the 17 BnaCIPKs, and they were BnaCIPK1, -5, -6, -9, -10, -11, -15 and -26 (Figure 4C). As for BnaCBL4, an ortholog of Arabidopsis SOS3(CBL4), it interacted with 14 BnaCIPKs, among which it interacted strongly with BnaCIPK1, -3, -5, -6, -7, -9, -10, -11, -15, -17, -24 and -26, but showed very weak interactions with BnaCIPK12 and -25 (Figure 4D). BnaCBL9 also exhibited a significant interaction with 12 out of 17 CIPKs, among which it interacted strongly with BnaCIPK7, -9, -15 and -26, less strongly with BnaCIPK3, -5, -6, -10, -11, -17 and -24, whereas a weaker interaction was observed between BnaCBL9 and BnaCIPK1 (Figure 4E). Lastly, BnaCBL10 was found to interact with only a small subset of seven BnaCIPKs, among which it interacted most strongly with BnaCIPK5, -10, -17 and -26, less strongly with BnaCIPK3,and -15 and, very weakly with BnaCIPK11 (Figure 4F). Looking at the interactions from a different point of view, we found that BnaCIPK14 and -23 did not show any interaction with any of the six BnaCBLs, suggesting they may interact with other unknown BnaCBL(s). On the contrary, BnaCIPK5, -10 and -26 showed interactions with all of the six BnaCBL, although the strength of interactions differed from one to another.

We then compared the interaction patterns of CBLs-CIPKs between canola and Arabidopsis to see to what extent differences existed between orthologs in two evolutionarily close species. However, a complete list of Arabidopsis CBL-CIPK interactions was not available. It has been reported that both Arabidopsis CBL1 and CBL9 interact with AtCIPK1, -5, -8, -11, -12, -17, -18, -23, -24 and -26, while AtCBL1 also interacts with AtCIPK7 and AtCBL9 with AtCIPK10, -16 and -20 [30,48]. Compared to our data, the shared interactors of AtCBL1 and BnaCBL1 were CIPK1, -5, -7, -8, -11, -17, -24 and -26, while the common interacting CIPKs of AtCBL9 and BnaCBL9 were CIPK1, -5, -10, -11, -17, -24 and -26, although we note that the full-length cDNA sequences of *BnaCIPK16, -18* and -*20* have not yet been cloned (Table 1). In addition, AtCBL3 was previously found to interact strongly with AtCIPK1, -2, -4, -6, -7, -11, -13 and weakly with AtCIPK9, -12 and -14, among these 10 CIPKs tested [6]. In comparison, in our data, BnaCBL3 interacted with BnaCIPK1, -6, -9 and -11, but not with BnaCIPK7 or -14. Lastly, Arabidopsis CBL4 did not show any interaction with AtCIPK1, -2, -4, -6, -7, -9, -11, -12, -13 and -14 [6]. However, in our experiments with canola, among the orthologs of the 10 AtCIPKs that AtCBL4 interacts with, BnaCBL4 interacted with BnaCIPK1, -6, -7, -9, -11 and -12. Overall, the interaction patterns between CBLs and CIPKs in Arabidopsis and canola showed several differences, which may be due to two reasons: the inherent biological differences between the two species and technical differences such as the vectors and yeast strains used. The results also show that predicting gene function in canola based on data from Arabidopsis does not produce accurate predictions in all cases. Comparison of CBL-CIPK interactions between canola and rice is not possible as a systematic study of OsCBL and OsCIPK interactions has not yet been reported.

Interestingly, the preferential interactions between BnaCBLs and BnaCIPKs, as reported in the Y2H assay above, do not appear to be consistent with the inferred phylogenetic relationships of the BnaCIPKs. For instance, of the closely related CIPK pairs, only BnaCIPK1 and BnaCIPK17 as well as BnaCIPK3 and BnaCIPK26 exhibited a similar interaction profile toward most of the six BnaCBLs assayed, that is, the former two interacted with BnaCBL1, -2 -4 and -9, and the latter two with BnaCBL1, -2 -4, -9 and -10 (Figure 4). On the other hand, the other three pairs of closely related duplicated CIPK pairs, which are BnaCIPK5/25, BnaCIPK8/24 and BnaCIPK9/23, displayed a rather different interaction profile, because, for example, BnaCIPK5 interacted with BnaCBL1, -2, -3, -4, -9 and -10, whereas BnaCIPK25 only interacted with BnaCBL1 and -4 and, BnaCIPK8 showed a preferential association with BnaCBL1 only, while BnaCIPK24 showed strong interaction with BnaCBL1, -4 and -9. Besides, BnaCIPK9 interacted with five of the six BnaCBLs tested, however, BnaCIPK23 did not show interaction with any of the six BnaCBLs, which was different from its counterpart (ortholog) in Arabidopsis [15,49]. Our findings indicate the necessity of empirically studying the canola CBL-CIPK network rather than simply making inferences from Arabidopsis. Similar observations were also made with CBL and CIPK complex formation in Arabidopsis [30]. Taken together, these data from a systematic Y2H assay indicate that sequence similarity and evolutionary history are not sufficient to predict CBL-CIPK interactions. Thus, understanding the exact structural features that determine the specificity of CBL-CIPK complex formation will help to explain why two closely related CIPK proteins show differential interaction profile towards CBLs. The structural analyses of the complexes formed between the regulatory domains of Arabidopsis CIPK14 and the calcium sensor CBL2 as well as between CIPK24(SOS2) and CBL4(SOS3) provide clear evidence of how and through which domain CBLs and CIPKs interact with each other [33,34]. Furthermore, analyses of the crystal structures also provide critical insights into the conformational change of CBL that occurs after binding of calcium ions through the EF-hand motifs and, the interaction of CIPK with CBL via the NAF/FISL motif within the C-terminal regulatory region.

The observed differences in interaction profile of pairs of closely related CIPK proteins might suggest that a high degree of conservation in the sequences existing between certain CBL or CIPK family members do not necessarily indicate that they are functionally redundant. For instance, Arabidopsis CIPK8 and CIPK24 (SOS2) are two phylogenetically closely related CIPKs, however their functions are quite different. AtCIPK8 regulates the low-affinity phase of the primary nitrate response [28], while CIPK24 (SOS2) is required for extrusion of Na^+^ from the cytosol to the extracellular matrix [13]. Furthermore, despite the fact that there is high similarity between BnaCBL1 and BnaCBL9 (90.6%, Additional file 2), the data presented here indicate BnaCBL1 can form a complex with BnaCIK8 and -11, whereas BnaCBL9 cannot, indicating that both Ca^2+^ sensor proteins could target specific CIPKs with different efficiency to respond to different stimuli. Similar finding was also made with Arabidopsis CBL1 and CBL9 [15,49-51].

Another intriguing question is why each of the 17 BnaCIPKs has evolved to interact with a different set of BnaCBLs. Presumably, different combinations of CBL-CIPKs respond to different exogenous and endogenous cues. For instance, in Arabidopsis, interaction of CBL1–CIPK1 is important to regulate salt stress, while interaction of CBL9–CIPK1 is essential for responses to ABA [50,52,53]. Research with the CBL-CIPK network in Arabidopsis and canola presented here suggests that there may be other factors that confer specificity to CIPK activity, which cannot be simply determined through Y2H detection of physical interactions with CBLs. One such factor could be cytosolic Ca^2+^ concentration ([Ca^2+^]_cyt_), since previous studies have demonstrated that different Arabidopsis CBL proteins show differential calcium binding efficiencies and CBL-CIPK complex formation may depend on Ca^2+^]_cyt_. For instance, free AtCBL2 binds only two Ca^2+^ ions, however, when it forms a complex with CIPK14, all four EF hands are occupied by Ca^2+^ ions [33]. In contrast, AtCBL4(SOS3) can bind four Ca^2+^ ions in a free state and binds only two Ca^2+^ ions upon interaction with CIPK24(SOS2) [34].

### Bimolecular fluorescence complementation (BiFC) assay of CBL-CIPK interactions using Yellow Fluorescent Protein (YFP)

**Figure 5:**
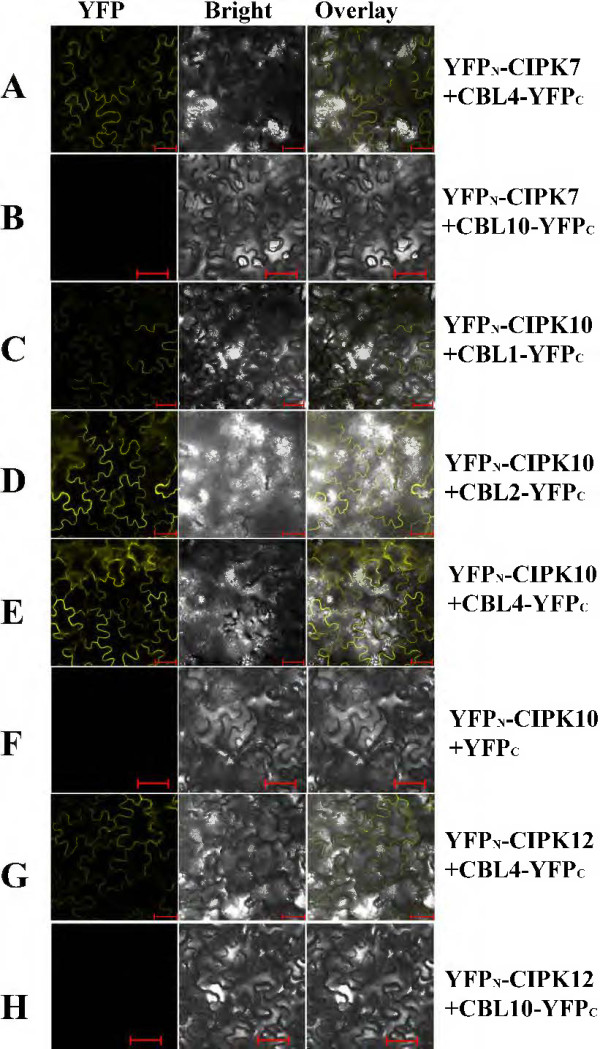
***In vivo *BiFC analysis of interaction between BnaCBL and BnaCIPK co-expressed in *N. benthamiana *leaf cells.** The coding regions of *BnaCBL* and *BnaCIPK* were fused to the N- and C-terminal halves of YFP, respectively. The fluorescence of YFP formed by the indicated plasmid combinations indicated at the right was observed 4 days after infiltration in *N. benthamiana* leaves by confocal laser microscopy. **(A)** YFP_N_-CIPK7 coexpressed with CBL4-YFPc; **(B)** YFP_N_-CIPK7 with CBL10-YFPc; **(C)** YFP_N_-CIPK10 with CBL1-YFPc; **(D)** YFP_N_-CIPK10 with CBL2-YFPc; **(E)** YFP_N_-CIPK10 with CBL4-YFPc; **(F)** YFP_N_-CIPK10 with YFPc; **(G)** YFP_N_-CIPK12 with CBL4-YFPc; **(H)** YFP_N_-CIPK12 with CBL10-YFPc. Bar = 50 μm.

We used BiFC to confirm *in planta* some of the interactions we observed between canola CBLs and CIPKs, in the Y2H assay. We chose three *BnaCIPKs*: (*BnaCIPK7*, *-10* and -*12*) because their orthologous genes are among the least-well characterized *CIPKs* in Arabidopsis. Co-expression of *YFP*_*N*_-*BnaCIPK* and *BnaCBL-YFPc* fusion genes or *YFPc* alone in leaf epidermal cells of *N. benthamiana* were performed through agrobacteria-mediated transformation. We observed that yellow fluorescence signals appeared in infiltrated leaf epidermal cells of *N. benthamiana* when *BnaCIPK7* and *BnaCBL4* were co-expressed (Figure 5A). In contrast, in controls in which a non-interacting CBL10 partner was used, no yellow signal was observed (Figure 5B). YFP signals also appeared when *BnaCIPK10* and *BnaCBL1, -2,* or -*4* were co-expressed (Figure 5C-E), while no signal of reconstructed YFP appeared in the control assay when *YFP*_*N*_-*CIPK10* was expressed together with *YFPc* only (Figure 5F). In addition, we observed strong YFP signal when BnaCIPK12 was co-expressed together with BnaCBL4 (Figure 5G), and in control leaf cells that expressed *BnaCIPK12* and *BnaCBL10*, we did not observe any YFP signal (Figure 5H). As for the intracellular locations of different CBL/CIPK complexes, research with Arabidopsis CBL/CIPK complexes shows that distinct targeting signals are located in the N-terminal domain of CBL proteins, for instance, the N-terminal MGCXXS/T motif mediating lipid modification by myristoylation and palmitoylation (S-acylation), determine the spatial localization of CBL/CIPK complexes [39,45]. Our aforementioned GFP study demonstrated that BnaCBL1 is located in the plasma membrane and nucleus, BnaCBL9 in cytoplasm and nuclei, and BnaCBL10 at the tonoplast. These observations, when combined with the BiFC results, allowed us to infer that the BnaCBL1/CIPK10 complex formed at the plasma membrane. However, the exact localization of other four BnaCBL/CIPK complexes could not be accurately determined as BnaCBL2, -4 localizations are not known yet. Nevertheless, part of the interaction patterns of BnaCBL-CIPK from our Y2H are all confirmed by the *in planta* BiFC assays, indicating the general reliability of the methods used here.

### Examining the domains of CIPK mediating interaction with CBL

**Figure 6:**
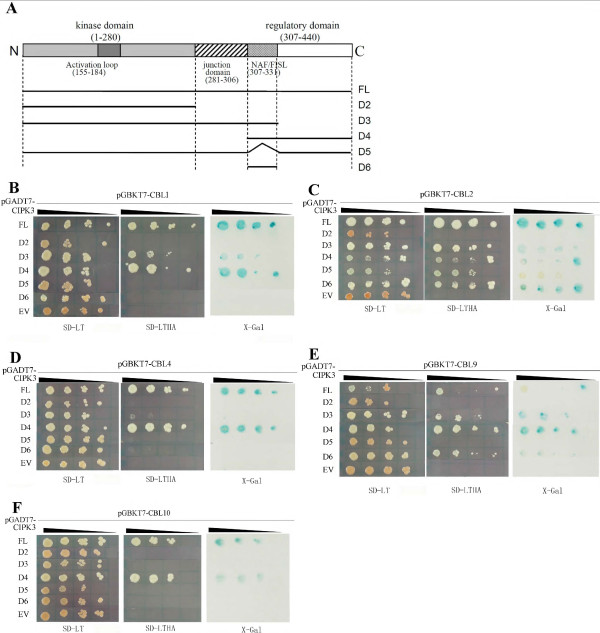
**Yeast two-hybrid analysis of interactions between CBLs and CIPK3 protein as well as its deletion versions. (A)** Schematic diagram of the domain structure of BnaCIPK3. It contains an N-terminal kinase domain and a C-terminal regulatory domain, separated by a short junction domain. In the regulatory domain, the position of the short NAF/FISL motif is indicated. FL represents the full-length BnaCIPK3 protein, and D2 through D6 represent the five deletion constructs cloned in the pGADT7 vector. **(B-F)** represent the interaction assay between BnaCBL1, -2, -4, -9 or -10 and various fragments of BnaCIPK3, respectively. In each panel, the yeast cells harboring the indicated plasmid combinations were grown on either the nonselective (SD-LT) or selective (SD-LTHA) media, followed by β-galactosidase assay (X-Gal staining). Decreasing cell densities in the dilution series are illustrated by narrowing triangles. EV is the pGADT7 vector.

Previous studies have shown that a short motif called NAF or FISL located in the C-terminal regulatory domain of CIPKs is necessary and sufficient for mediating interactions with CBLs [6,7]. To test this in the canola CBL-CIPK network, we generated a series of deletion constructs (D2-D6) by cloning *BnaCIPK3* fragments into the pGADT7 activation domain vector (Figure 6A). These constructs were then transformed separately into yeast cells harboring pGBKT7-BnaCBL1, -2, -3, -4, or -9 plasmid, respectively, and interactions were assayed by growth on selective medium as an activation of nutritional marker gene *HIS3* and *LacZ* reporter gene (Figure 6B-F). The results demonstrated that BnaCIPK3-D2 or BnaCIPK3-D5 protein, which did not contain the NAF/FISL motif (amino acids 307–331), did not interact with any of the five BnaCBLs tested (Figure 6B-F). This indicates that deletion of a NAF/FISL motif from BnaCIPK3 is sufficient to abolish the interactions with CBLs. On the other hand, BnaCIPK3-D3, which contains the N-terminal kinase domain plus NAF/FISL motif, interacted strongly with BnaCBL1, -2 and -9, but weakly with BnaCBL4 and did not interact with BnaCBL10 (Figure 6B-F). BnaCIPK-D4, which contains the C-terminal region plus NAF/FISL motif, showed interactions with all of the five BnaCBLs assayed (Figure 6B-F). In addition, the D6 construct harboring only the NAF/FISL motif of BnaCIPK3 showed significant interactions with BnaCBL2 and -9, but not with BnaCBL1, -4 or -10 for unknown reasons (Figure 6B-F). As a control, yeast cells transformed with any of the five pGBKT7-BnaCBL plasmids and the empty pGADT7 (EV) plasmid did not show any growth on the selective medium SD-LTHA, indicating that observed interactions had high specificity (Figure 6B-F, bottom rows). In addition, yeast cells expressing the full-length (FL) BnaCIPK3 and four of the five BnaCBL genes showed good growth on the selective medium, except BnaCBL9, which showed limited growth for unknown reasons (Figure 6B-F, top rows). We therefore hypothesized that other region(s) of some specific CIPK protein may also determine the interaction with CBL proteins. A previous study showed that the N-terminal region of CIPKs is also involved in determining the specificity of CBL-CIPK interaction [54].

### Expression analysis of BnaCBL and BnaCIPK genes in response to abiotic stress and hormone stimulus

**Figure 7:**
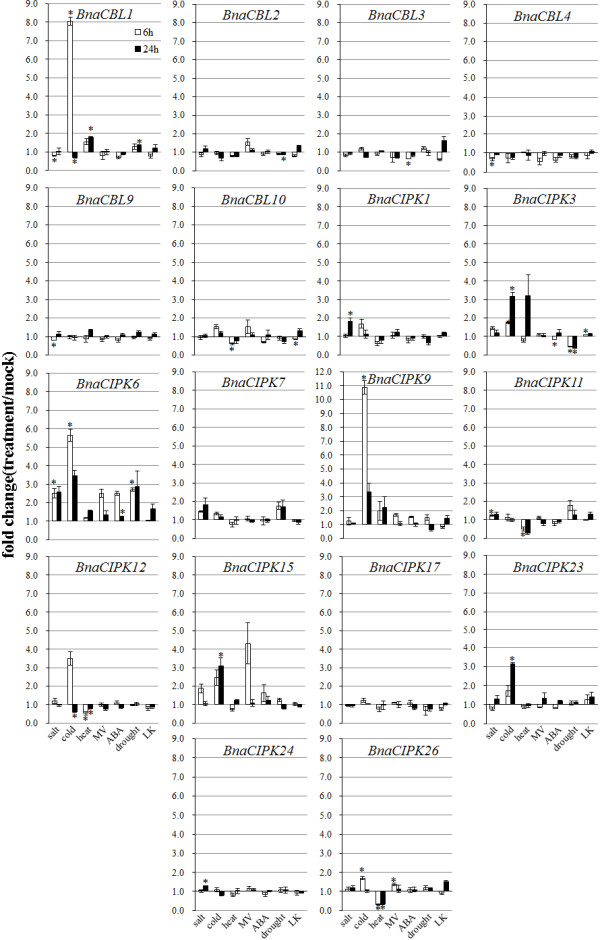
**Expression analyses of BnaCBL and BnaCIPK genes in response to a variety of treatments, including 200 mM NaCl, 50 μM ABA, 10 μM Paraquat (Methyl viologen, MV), cold (4°C), heat (37°C), drought and low potassium (LK) treatments.** Data is the mean (treatment/mock, linear scale) of three biological replicates ± S.E. Asterisks denote significant differences between treated and mock plants by Student *t*-test (*p* ≤ 0.05).

To investigate whether BnaCBL and BnaCIPK genes are involved in canola responses to abiotic stresses and stress hormone-ABA, quantitative RT-PCR was used to analyze expression patterns of six BnaCBL and 12 BnaCIPK genes in 18 d old canola seedlings under salt, cold, heat, oxidative (MV), drought, low potassium (LK) stress treatments as well exogenous ABA application (Figure 7).

Data from three biological replicates showed that in salt-treated canola seedlings, *BnaCBL1, -4* and -*9* were down-regulated at the 6 h time-point and the other three BnaCBL genes did not show significant changes at either time point tested. *BnaCBL1* transcript level showed a significant increase 6 h after cold treatment, however, it was down-regulated at 24 h time-point. In 24 h cold-treated seedlings, only *BnaCBL10* was still slightly up-regulated and expression of the *BnaCBL2, -3, -4* were down-regulated. However, *BnaCBL9* expression seemed not to be affected by cold treatment at 6 or 24 h time-point. As for heat treatment, it induced the expression of *BnaCBL1* and -*9* at both time-points and 24 h time-point only, respectively. On the other hand, heat stress significantly down-regulated the expression levels of *BnaCBL10* at 6 h time-point. Under the treatment of methyl viologen (MV, Paraquat), a reagent that induces the production of oxidative stress, no BnaCBL genes showed significant changes at either time-point assayed. In ABA-treated canola seedlings, no BnaCBL genes were significantly induced while *BnaCBL3* was repressed at 6 h time-point. Drought or water deficiency stress increased accumulation of *BnaCBL1* transcripts and down-regulated *BnaCBL2* expression at 24 h time-point. Lastly, LK treatment significantly down-regulated *BnaCBL10* expression at the early 6 h time-point, however, it slightly increased the expression levels of *BnaCBL1, -2, -3,* and -*10* at 24 h time-point (Figure 7).

As for the 12 BnaCIPK genes assayed, among the seven treatments applied, the most striking induction was observed for *BnaCIPK3, -6, -9, -12, -15, -23* and -*26* after cold treatment (Figure 7). Furthermore, the transcript level of BnaCIPK6 gene showed the most significant increases after various stress treatments (Figure 7). Salt treatment induced the expression of *BnaCIPK1, -3, -6, -7, -11, -15* and -*24* at either 6 or 24 h time-point. In heat-treated seedlings, the expression levels of *BnaCIPK3* and -*6* were up-regulated, whereas those of *BnaCIPK1, -11, -12* and -*26* down-regulated. As for MV treatment, it up-regulated the expression of *BnaCIPK6, -9, -15* and -*26* at 6 h time-point and no significant changes were observed for the other eight BnaCIPK genes at any time-point. ABA treatment up-regulated *BnaCIPK6* expression at both time-points, and repressed *BnaCIPK3* expression at 6 h time-point. As for drought treatment, only *BnaCIPK3* was repressed and *BnaCIPK6* induced at both time-points, the others did not show any significant changes. In LK-treated canola roots, the expression levels of *BnaCIPK-3, -6, -11* and -*26* were slightly induced, and no significant changes were observed with the other BnaCIPK genes at either of the two time-points assayed (Figure 7).

Taken together, the expression data of six BnaCBL and 12 BnaCIPK genes in canola seedlings or roots after various stress treatments suggests that different CBL or CIPK genes may participate in the signaling process to a single stress and, a single CBL or CIPK likely plays a role in multiple stress responses.

### Comparison of expression profiling of CBL and CIPK genes in canola, Arabidopsis and rice under abiotic stress conditions and ABA treatment

Keeping in mind that canola CBL and CIPK interaction patterns showed differences compared to their orthologs in Arabidopsis, we were curious whether responses to of CBL and CIPK transcripts to abiotic stress or hormone stimuli also differed between canola, Arabidopsis, and rice. To examine the expression profiles of Arabidopsis CBL and CIPK genes to cold, salt, ABA, heat, oxidative and LK stresses, we extracted data from publicly available microarray data sets [55,56]. Detailed data sources and results of expression changes of the two family genes in Arabidopsis under stress conditions are provided in Additional file 11. For the comparison, we focused only on those Arabidopsis genes whose orthologs in canola have been cloned and assayed by qRT-PCR as described above. We also limited our analysis to those microarray data sets with described treatments and tissues comparable to what we reported herein, for example, Paraquat (MV) but not hydrogen peroxide for oxidative stress, and aerial tissues but not roots. Among *AtCBL1, -2, -3, -4, -9* and -*10*, only *AtCBL1, -2* and -*9* were significantly regulated by ABA treatment and only *AtCBL1, -4, -9* and -*10* were significantly regulated by cold stress, with *AtCBL1* up-regulated and the other three down-regulated. We also found that the abundance of only *AtCBL4* and -*9* transcripts was down-regulated by drought treatment. Heat stress (38°C) repressed the expression of *AtCBL4* and -*9* genes. Salinity stress, down-regulated the transcription of *AtCBL2, -4* and -*10.* AtCBL3 was slightly up-regulated by MV treatment and no AtCBL genes were significantly influenced by LK treatment (Additional file 11). Compared to our data, we found that responses of BnaCBL1 gene to cold, heat and drought stresses were similar to AtCBL1 while the responses of *CBL2, -3, -4, -9* and -*10* showed both very limited consistency between Arabidopsis and canola, although there were quite many CBL genes that showed only minor changes under different stress treatments (Additional file 11).

For the CIPK genes, we found that among the 11 CIPK genes analyzed both in canola by qRT-PCR (Figure 7) and assayed in Arabidopsis through microarray (Additional file 11), 56% (9/16) of CIPK genes were up- or down-regulated under ABA treatment, with *AtCIPK3* and -*15* repressed and the other seven induced by ABA. Cold stress significantly increased the abundance of transcripts of *AtCIPK7, -9, -11,* and -*12* while decreased that of *AtCIPK17* at a late time-point. On the other hand, *AtCIPK6, -9, -11* and -*23* were significantly up-regulated by drought stress, and *AtCIPK15* and -*17* were down-regulated by drought treatment. Heat stress significantly increased the transcript abundance of *AtCIPK1, -11, -15,* and -*24* among the 16 AtCIPK genes examined. As for salinity treatment, it induced the expression of *AtCIPK6, -7, -9, -11, -12* and repressed the expression of *AtCIPK*-3, -17 and -24. However, only *AtCIPK23* was up-regulated by oxidative stress and the other 15 AtCIPK genes did not show any significant change to it. LK treatment slightly increased the transcript abundance of *AtCIPK9* while decreased that of *AtCIPK17* (Additional file 11). When the expression profiles of *AtCIPKs* and *BnaCIPKs* to these stresses were compared, we found again that both similarities and differences existed between them under specific stress conditions. For example, the responses of *CIPK7* and -*9* to cold stress were similar in canola and Arabidopsis, while those of the other genes were different. Salinity stress induced the expression of *CIPK6* and -*11* both in Arabidopsis and canola, however the expression changes of the others were quite different (Figure 7, Additional file 11).

We next compared our canola CBL and CIPK qRT-PCR data to publically available transcript expression data of these two genes families measured in rice under five different stress conditions (salt, cold, heat and drought and LK) using the rice 57 K microarray datasets [57-60] with detailed results presented in Additional file 12. For the six BnaCBL and 17 BnaCIPK genes cloned in the present study, we were only able to identify orthologous pairs for four CBL and 13 CIPK genes from rice (Table 1). Among the ten OsCBL genes, we found that all OsCBL genes were up- or down-regulated under one or more stress conditions (Additional file 12). For instance, *OsCBL1* and -*2* were up- and *OsCBL4* was down-regulated by cold stress while *OsCBL2*, *-4* and -*9* were up-regulated by heat treatment. We observed both similarities and differences in responses to abiotic stresses existed between rice and canola CBL orthologs. For example, *OsCBL1* and *BnaCBL1*, an orthologous pair, were induced by cold stress. On the other hand, orthologous *OsCBL10* and *BnaCBL10* were induced and repressed by drought stress treatment, respectively (Additional file 12).

Among the 34 OsCIPK genes, we found that 94% (32/34) of OsCIPK genes were up-or down-regulated under one or more stress conditions. However, the changes of *OsCIPK20* and -*28* to any stresses were not significant (Additional file 12). For example, *OsCIPK2, -7, -9, -14/15, -19, -24, -26* and -*27* were up-regulated by cold treatment while *OsCIPK1, -4, -5, -6, -8, -11, -12, -18, -22, -23* and -*32/33* were down-regulated by cold stress. Higher temperature (heat stress) significantly induced the expression of *OsCIPK6, -8, -14/15, -24, -25, -29* and repressed the expression of *OsCIPK1, -2, -4, -5, -7, -9, -10, -12, -16, -17, -19, -21, -22, -23, -27* and -*32/33*. Likewise, when the orthologous pairs between rice and canola were compared, similar changes were observed with some pairs while different responses to abiotic stresses were identified for other pairs. For instance, transcript abundance of *OsCIPK6* and *OsCIPK9* was increased by drought and cold treatment, respectively, which is similar to their orthologous gene *BnaCIPK6* and *OsCIPK9*, respectively (Figure 7). On the other hand, *OsCIPK5* was down-regulated by cold or heat stress, which is different from its ortholog *BnaCIPK6. OsCIPK23* was also down-regulated by cold treatment, and this is different from its orthologous gene *BnaCIPK23*. Taken together, the comparative analysis suggested that some orthologous gene pairs between different species may have retained similar functions while others (especially when canola was compared to rice) likely evolved divergent functions in response to abiotic stress or ABA stimuli between monocot and dicot species, as supported by a recent research with three basic helix-loop-helix (bHLH) genes controlling stomatal development [42].

### Functional analysis of canola CIPK24 gene in Arabidopsis

**Figure 8:**
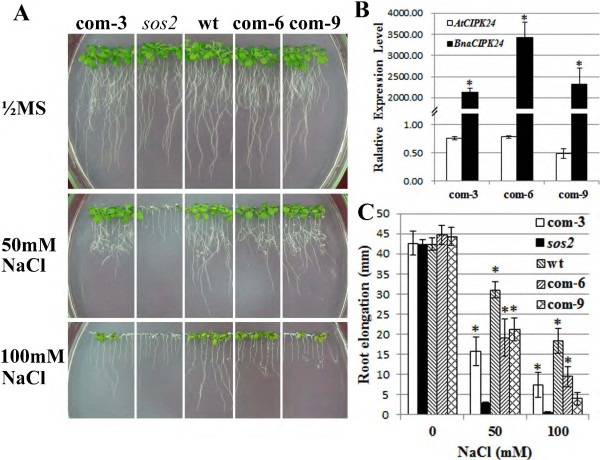
**Functional complementation of Arabidopsis *sos2-1 *mutant by canola CIPK24 gene. (A)** 4 DPS (days post-stratification) seedlings of wild-type (wt), *sos2-1* mutants and complementary lines com-3, com-6 and com-9 were transferred to ½ x MS medium containing 0, 50 and 100 mM NaCl, respectively. The pictures were taken after 7 days of treatment. **(B)** qRT-PCR assay of BnaCIPK24 transgene expression levels in three independent transgenic lines in the wild-type Arabidopsis. Data is the mean (com/wt, linear scale) of three biological replicates ± S.E. Asterisks denote significant differences of *BnaCIPK24* transcript abundance between transgenic lines and wt seedlings by Student *t*-test (*p* ≤ 0.05). The transcript levels of *AtCIPK24* (*SOS2*) in the transgenic lines compared to that in *wt* was also calculated. **(C)** Seedling root elongation was measured at day 7 after the transfer. Error bars represent S.E. (n = 24). Asterisks denote significant differences between wt or transgenic and *sos2* mutant plants by Student *t*-test (*p* ≤ 0.05).

It had been reported that Arabidopsis mutants in the SOS pathway, for instance, *sos1, sos2* and *sos3*, are specifically hypersensitive to high external Na^+^ concentrations [13,14]. To dissect the biological function of canola CIPK genes, we constructed transgenic Arabidopsis lines heterologously expressing BnaCIPK24 gene in the *sos2-1* mutant background for functional complementation test. Through qRT–PCR analysis, we confirmed the high level expression of BnaCIPK24 transgene in the *sos2-1* background (Figure 8B). We selected three representative T_3_ generation lines that were independently complemented lines (com-3, com-6 and com-9) for phenotypic assays. On normal medium plates, wild type (wt), *sos* mutants and three complemented lines displayed relatively similar growth phenotypes, although roots of the *sos2-1* seedlings were slightly shorter (Figure 8A, top panel). When treated with 50 mM NaCl, growth of *sos2* was inhibited compared with wild type plants. Expression of *BnaCIPK24* in *sos2-1* substantially alleviated the growth retardation imposed by moderate salt stress (Figure 8A, middle panel). Under 100 mM NaCl treatment, growth inhibition of *sos2* mutants was even more pronounced. However, the *sos2-1* mutants expressing *BnaCIPK24* could survive much better (Figure 8A, bottom panel). Statistical analysis of root elongation showed that all the three complementation lines were generally restored to a wild-type level of salt tolerance, which is highly correlated to the expression level of the transgene in Arabidopsis (Figure 8C). These data indicate the canola CIPK24 gene could substitute for the corresponding Arabidopsis CIPK24 (SOS2) component, and is a functional ortholog of its Arabidopsis counterpart.

## Conclusions

During their evolution, plants have acquired a set of complex and highly tuned signal transduction pathways to respond to adverse environmental conditions. Research during the last few decades has revealed that calcium signaling is as an integral process that enables plants to respond to numerous external stimuli and coordinate essential processes. The CBL–CIPK network is an important example of a plant calcium signaling pathway [9,16]. CBL–CIPK network members in Arabidopsis and rice have been demonstrated to act as convergence points or master switches for distinct signals, for example, Arabidopsis CIPK23 positively regulates both potassium and nitrate nutrition [12,15,49] and Arabidopsis CIPK24/SOS2 activates transporters that extrude toxic sodium ions from the cytoplasm to the apoplast or the vacuole through alternative interactions with CBL4 or CBL10, respectively [20,21,61]. Although functions of some members of Arabidopsis or rice CBL and CIPK have been studied, functions of many CBLs and CIPKs from Arabidopsis and rice remain uncharacterized to date. In addition, our understanding of the CBL–CIPK network in other plants remains fragmentary. So far, no systematic analysis of the CBL or CIPK family genes has been reported in the important oil crop canola.

In this present study, we carried out a detailed survey of the two gene families in canola and characterized them on the bases of phylogenetic relationship, conserved protein motifs, gene duplication, interactions between them and expression profiles to abiotic stress and the stress hormone ABA treatments. We found that each canola *CBL* and *CIPK* exhibited differential responses to multiple stress treatments, suggesting that they were major convergence points for cross-talk between different signal transduction pathways. On the other hand, multiple CIPKs seemed to be necessary to coordinate with one specific stress stimulus. For instance, transcription of eight canola CIPK genes appeared to be affected by cold treatment, indicating the involvement of multiple CBL-CIPK signaling pathways in response to cold stress. In addition, we comparatively analyzed the expression profiles of putative orthologous or paralogous pairs existing in the CBL or CIPK family genes of canola, Arabidopsis and rice under different stress or hormone treatments. Therefore, our genomic, bioinformatic and experimental analyses of the two family genes and proteins provide a solid foundation for the further functional characterization of the CBL-CIPK network involving in decoding calcium signals under different stress conditions. Taken together, by further understanding the functions and inner mechanisms of the CBL–CIPK network in canola through virus-induced gene silencing (VIGS) and overexpression, as underway in our lab, we would be able to elucidate how the canola CBL–CIPK network enables integration of multiple signals of the plant’s environment and coordinates downstream responses to stresses such as toxic ion exposure, extreme temperatures and nutrient deprivation.

## Methods

### BnaCBL and BnaCIPK gene identification

For the identification of expressed sequence tags (ESTs) coding CBL and CIPK homologues in canola, we followed previously described methods with minor modifications [62]. In brief, the full-length cDNA of 10 Arabidopsis CBL and 26 CIPK genes downloaded from TAIR (Version 10) (http://www.arabidopsis.org) were used to search against the EST database (release January 1, 2011) at NCBI (National Centre for Biotechnology Information; http://www.ncbi.nlm.nih.gov/dbEST/index.html). Only those EST hits with e-values lower than 10^-4^ were retrieved. These ESTs were further filtered, clustered, and assembled. The resultant contigs and singletons were reciprocally searched against the Arabidopsis database to identify the best hit among all the 10 AtCBL and 26 AtCIPK genes, for each contig and singleton, which is the putative ortholog.

### Plant growth and gene cloning

Wild type canola (DH12075) plants were grown in Pindstrup soil mix (Denmark) in the greenhouse with a photoperiod of 16 h light (T8 fluorescent tubes with a light intensity of approximately 100 μE m^-2^ s^-1^)/8 h dark, and a temperature of 22°C day/18°C night for 7 d. Young leaves were harvested for RNA isolation using the Plant RNA kit (Omega bio-tek, USA). RNA integrity was checked by electrophoresis on an agarose gel and quantified using the NanoDrop 1000 (NanoDrop Technologies, Inc., USA). 2.5 μg of total RNA were used to synthesize cDNAs by using RNase H minus MMLV (Fermentas, USA) and oligo(dT)_18_ (Fermentas). PCR was conducted in a 50 μL final volume including 0.5 μL of cDNA template, 1 × *Pfu* buffer, 200 μM deoxynucleotide triphosphates (dNTPs), 400 nM of each primer, and 1.25 units of *Pfu* DNA polymerase. The PCR conditions included an initial denaturation at 94°C for 3 min, followed by 35 cycles of 94°C for 30 s, 50°C for 30 s, 72°C for 1 min per kb, with a final extension at 72°C for 8 min. RACE (rapid amplification of cDNA end) was conducted as described previously [62]. The primers used are listed in the Additional file 13. PCR products were gel purified using the BioSpin Gel Extraction Kit (Bioer Technology Co., Ltd) and cloned into pJET1.2 vector supplied with the CloneJET PCR cloning kit (Fermentas) and sequenced from the two ends using BigDye reagenton an ABI3700 sequencer (Applied Biosystems).

### Phylogenetic tree construction and bioinformatics

The *CBL* and *CIPK* sequences of Arabidopsis were downloaded from TAIR10 (http://www.arabidopsis.org). To identify *CBLs* and *CIPKs* from other species, firstly, we aligned the 10 AtCBLs and 26 AtCIPKs and generated a hidden Markov model (HMM) for each; secondly we performed a HMM-based search (http://hmmer.janelia.org/, [63] for similar peptide sequences in the sequenced genomes stored in Phytozome v9.0 (http://www.phytozome.net/) and also by keyword search in the NCBI database. After that, we retrieved and inspected putative CBL sequences for the conserved four EF-hand motifs and each putative CIPK was searched for the NAF/FISL signature motif sequences. The amino acid sequences of the canola CBL and CIPKs were deduced form the putative ORFs using DNAMAN software. The phylogenetic trees of CBL and CIPK proteins from various species were constructed as described previously [62]. In brief, the predicted amino acid sequences of *BnaCBLs* or *BnaCIPKs* were aligned using ClustalX1.83 program with the same multiple alignment parameter settings as before. By using the maximum parsimony (MP) algorithm implemented in MEGA5.1 software [64], the phylogenetic trees were constructed. The amino acid sequence of others species including *Arabidopsis*, rice (*Oryza sativa*. subsp *japonica*), maize (*Zea mays*), tomato (*Solanum lycopersicum*), *Brachypodium distachyon*, cotton (*Gossypium hirsutum*)*, Gossypium raimondii, Physcomitrella patens,* pea *(Pisum sativum),* sorghum (*Sorghum bicolor)*, with the CBL or CIPK identified from *Ostreococcus tauri*[9,45] was used to root the CBL and CIPK trees, respectively.

The pairwise identity and similarity of proteins were calculated by program MatGAT v2.02 (http://bitincka.com/ledion/matgat/). The domain analysis was performed using SMART (http://smart.embl-heidelberg.de/smart/set_mode.cgi?NORMAL = 1) and other programs like PROSITE. The palmitoylation sites were predicted by CSS-Palm 3.0 (http://csspalm.biocuckoo.org/) and, myristoylation sites by Myristoylator (http://web.expasy.org/myristoylator/). The motif logos were analyzed and generated by using the corresponding protein sequences in MEME 4.9.0 (Release date: Wed Oct 3 11:07:26 EST 2012) with default parameters. The subcellular locations were predicted through three different programs, one is PSORT (http://psort.nibb.ac.jp), the second is CELLO v2.5 (http://cello.life.nctu.edu.tw) and the third is ESLPred (http://www.imtech.res.in/raghava/eslpred/index.html). TMHMM (http://www.cbs.dtu.dk/services/TMHMM-2.0/) was used to predict transmembrane helices (TMHs) of CBL or CIPK proteins. The numbers of introns of *AtCIPK* and *OsCIPK* genes were determined by comparison of genomic sequences to the cDNA sequences of respective genes. The expression profilings of CBL and CIPK genes in Arabidopsis and rice were analyzed through public datasets using the Affymetrix Arabidospsis 22 k ATH1 [55,56] and rice 57 k GeneChip [57,59,60] , respectively, which are stored in Genevestigator (https://www.genevestigator.com/gv/plant.jsp). For Arabidopsis, *CIPK26* gene has no probe in ATH1 probe set and, for rice, *OsCIPK14* and *OsCIPK15* (97.63% identity at the nucleotide level) share a probe and *OsCIPK32* and *OsCIPK33* (95.42% identity at the nucleotide level) share another probe, because of high identity of cDNAs.

### Subcellular localization and confocal microscopy

The coding region of selected BnaCBL and BnaCIPK genes was amplified by PCR using *Pfu* DNA polymerase from the corresponding plasmids with primers listed in Additional file 13. The PCR products were cloned upstream of the GFP gene in the pCsGFPBT binary vector (GenBank: DQ370426) with a Gly-Ala rich peptide linker between CDSs and GFP. Similarly, plasma membrane and tonoplast markers, which were AtCBL1n [39] and AtTPC1 (At4G03560) [47], were fused in-frame before mCherry reporter gene in the pYJmCherry binary vector, modified from pBS-mCherry transient vector. After confirmation by sequencing, these constructs and the p19 protein of tomato bushy stunt virus were separately transferred into *Agrobacterium tumefaciens* GV3101 for infiltration into leaves of *N. benthamiana*[65]. Freshly made transformed Agrobacteria cell cultures were resuspended in media containing 10 mM MES-KOH (pH 5.6), 10 mM MgCl_2_ and 0.15 mM acetosyringone (AS, Sigma), adjusted to an OD_600_ of 0.8, before being mixed at a volume ratio of 1:1:1 (construct: marker: p19). Three milliliters of the culture were taken by sterile single-use syringes to inject into the abaxial air space of *N. benthamiana* leaves. The leaf section near the injection site was squashed 2 d after infiltration and the signals of GFP and mCherry were excited at 488 and 587 nm, respectively, and were examined under an A1 confocal microscope (Nikon, Japan). The signals from three independent squashes were examined and representative images were presented.

### Yeast two-hybrid (Y2H) analysis

Yeast two-hybrid analysis was performed using the MatchMaker yeast two-hybrid system (Clontech, USA). Firstly, the coding regions of BnaCBL and BnaCIPK genes were subcloned into pGBKT7 and pGADT7 vectors, respectively. Primers used are listed in Additional file 13. Then, the plasmids were sequentially transformed into yeast strain AH109 through the lithium acetate method following the protocol described in Yeast Protocols Handbook (Clontech). After plated on three sets of media, SD-Leucine-Tryptophan (SD-LT), SD-Leucine-Tryptophan-Histidine supplemented with 5 mM 3-Amino-1,2,4-triazole(3-AT, Sigma)(SD-LTH + 3-AT), and SD-Adenine-Histidine-Leucine-Tryptophan (SD-LTHA), the yeast colonies were grown at 30°C for 2 d (SD-LT plastes) or 7 d (SD-LTH + 3-AT and SD-LTHA plates ) before photographed. For the deletion assay, different fragments of BnaCIPK3 gene were cloned into pGADT7 vector through typical restriction-ligation method using the primers listed in Additional file 13.

The putative positively interacting transformants were cultivated in YEPD (1% yeast extract, 2% peptone and 2% glucose) media for serial dilution. In brief, the exponentially grown yeast cells were centrifuged at 5000 *g* for 5 min at room temperature and adjusted to OD_600_ = 0.5 with sterilized double-distilled water. It was then diluted 1/10, 1/100 and 1/1000. Two microliters of the aforementioned serial diluted yeast cells were spotted onto SD-LT, SD-LTH + 3-AT and SC-LTHA media, grown at 30°C for 2 d (SD-LT plates) or 5 d (selective plates) before photographed.

The colony-lift filter assay was conducted following the instruction in the Yeast Protocols Handbook. The freshly grown colonies (5 d) on the selection media (SD-LTHA) were transferred onto a sterilized 9 cm filter paper and then flash frozen in liquid nitrogen for 10–20 seconds. After thawing completely, the filter paper (carried colony side up) was transferred onto presoaked filter paper in 5 ml of staining buffer (60 mM Na_2_HPO_4_,39.8 mM NaH_2_PO_4_, 10 mM KCl, 1 mM MgSO_4_, 0.817 mM 5-bromo-4-chloro-3-indolyl-β-D-galactopyranoside [X-gal] and 38.5 mM β-mercaptoethanol ) in 90 mm petri dish for 8–12 h at 37°C. After that, the reaction was stopped and filter paper dried before photographed. The Y2H, titration and X-gal assays were repeated in at least three independent experiments. Pictures were assembled in Adobe Photoshop CS (Adobe System Inc.).

### Bimolecular fluorescence complementation (BiFC) assay

To generate the BiFC constructs, the coding regions of *BnaCIPKs* with stop codons were subcloned into 35S-SPYNE(R)173, and the coding regions of *BnaCBLs* without stop codon were subcloned into 35S-SPYCE(M) vector [66] . For transient expression, the *Agrobacterium tumefaciens* strain GV3101 carrying each construct was used together with the p19 strain for infiltration of 5-week-old *Nicotiana benthamiana* leaves [67]. For microscopic observation, the reconstructed yellow fluorescence protein (YFP) signals of the lower epidermal cells of leaves cut 4 d after infiltration were examined using a Nikon A1 confocal microscope (Nikon, Japan). Three independent squashes were prepared and observed for each combination of BiFC plasmids. Images were assembled in Photoshop CS (Adobe System Inc.).

### Stress treatments and quantitative RT-PCR (qRT-PCR)

Wild type canola (DH12075) plants were grown in a greenhouse with a photoperiod of 16 h light /8 h dark. 18 d old plants were applied with abiotic stress and hormone treatments beginning at 9:45 in the morning (2.75 h after light). Salinity was increased by irrigating soil with 200 mM NaCl. Cold and heat treatment was applied by putting the plants in 4**°** or 37°C with light. ABA was applied through spraying plants with 50 μM (±)-ABA (Invitrogen, USA) and oxidative stress was performed by spraying with 10 μM Paraquat (Methyl viologen, Sigma). Drought was subjected on 14 d old plants by withholding water until light or severe wilting occurred. For low potassium (LK) treatment, a hydroponic system using a plastic box and plastic foam was used (Additional file 14) and the hydroponic medium (1/4 x MS, pH5.7, Caisson Laboratories, USA) was changed every 5 d. LK medium was made by modifying the 1/2 x MS medium, such that the final concentration of K^+^ was 20 μM with most of KNO_3_ replaced with NH_4_NO_3_ and all the chemicals for LK solution were purchased from Alfa Aesar (France). The control plants were allowed to continue to grow in fresh-made 1/2 x MS medium. Above-ground tissues, except roots for LK treatment, were harvested at 6 and 24 hours time points after treatments and flash-frozen in liquid nitrogen and stored at -80°C. The planting, treatments and harvesting were repeated three times independently.

Quantitative reverse transcriptase PCR (qRT-PCR) was performed as described earlier with modification [62,68,69]. Total RNA samples were isolated from treated and non-treated control canola tissues using the Plant RNA kit (Omega, USA). RNA was quantified by NanoDrop1000 (NanoDrop Technologies, Inc.) with integrity checked on 1% agarose gel. RNA was transcribed into cDNA by using RevertAid H minus reverse transcriptase (Fermentas) and Oligo(dT)_18_ primer (Fermentas). Primers used for qRT-PCR were designed using PrimerSelect program in DNASTAR (DNASTAR Inc.) targeting 3′UTR of each genes with amplicon size between 80 and 250 bp (Additional file 13). The reference genes used were *BnaUBC9* and *BnaUP1*[70]. qRT-PCR was performed using 10-fold diluted cDNA and *SYBR Premix Ex Taq*^*TM*^ kit (TaKaRa, Daling, China) on a CFX96 real-time PCR machine (Bio-Rad, USA). The specificity of each pair of primers was checked through regular PCR followed by 1.5% agarose gel electrophoresis, and also by primer test in CFX96 qPCR machine (Bio-Rad, USA) followed by melting curve examination. The amplification efficiency (E) of each primer pair was calculated following that described previously [62,68,71]. Three independent biological replicates were run and the significance was determined with SPSS (*p* < 0.05).

### Arabidopsis transformation and phenotypic assay

A 3.5-kb fragment including the *BnaCIPK24* coding region and 1500 bp of the 5'flanking DNA upstream of the ATG codon was amplified by PCR from canola genomic DNA with gene-specific primers. The PCR product was cloned into the binary vector pCAMBIA1303 (CAMBIA, Canberra, Australia) using *Sal* I and *Bst*E II restriction sites. The construct was transformed into *Agrobacterium tumefaciens* strain GV3101 and introduced into Arabidopsis *sos2-1* mutant plants by the floral dip method (Clough and Bent, 1998). Transgenic seeds were selected on half-strength MS medium containing 0.8% (w/v) Phytoblend (Caisson labs, USA), and 30 mg/L hygromycin B (Roche). The resistant seedlings were transplanted to soil and grown in the greenhouse to produce seeds. Homozygous T_3_ lines were first used to examine the expression levels of *BnaCIPK24* and *AtCIPK24* in Arabidopsis through qRT-PCR. Three independent high expression lines were used for the post-germination assay.

Seeds of the wild type (Col-0), the *sos2-1* mutant, and the aforementioned transgenic T_3_ line were sterilized in 2.65% bleach containing 0.03% Tween-20, then planted in triplicate on ½ x MS medium with 1% sucrose solidified with 0.8% Phytoblend, and stratified in 4°C for 3 d before transferred to a growth chamber with a photoperiod of 16 h light/8 h dark at the temperature 22–23°C. After vertically growing for 4 d, seedlings were transferred onto ½ x MS medium supplemented with or without 50 or 100 mM NaCl and continued to grow vertically for another 7 d, before the root elongation was measured and plates photographed.

### Accession numbers

The cDNA sequences of canola CBL and CIPK genes cloned in this study were deposited in GenBank under the accession No. JQ708046- JQ708066 and KC414027- KC414028.

## Competing interests

The authors declare that they have no competing interests.

## Authors’ contributions

YQJ and BY designed, supervised and carried out parts of the experiments and wrote the manuscript. HZ, WZL, HL, LW, BW, WL performed the experiments. MKD provided material, and helped in data analysis and writing. All authors read and approved the manuscript.

## Supplementary Material

Additional file 1 BnaCBL and BnaCIPK EST summary. Click here for file

Additional file 2 Amino acid residue identity and similarity of BnaCBL and BnaCIPK proteins compared with each other and with those from Arabidopsis and rice. Click here for file

Additional file 3 Analysis of EF-hand motifs in calcium binding proteins of representative species. Click here for file

Additional file 4 Multiple alignment of canola CIPK proteins and motif analysis. Click here for file

Additional file 5 List of identified calcineurin B-like (CBL) genes in other species. Click here for file

Additional file 6 List of identified CBL-interacting protein kinase (CIPK) genes in other species. Click here for file

Additional file 7 Multiple alignment of 34 rice CIPK proteins. Click here for file

Additional file 8 Phylogenetic analysis of CBL proteins from a variety of species. Click here for file

Additional file 9 Phylogenetic analysis of CIPK genes from a variety of species. Click here for file

Additional file 10 Subcellular localization predictions of BnaCBL and BnaCIPK proteins. Click here for file

Additional file 11 Arabidopsis CBL and CIPK expression profiles to different abitoic stresses. Click here for file

Additional file 12 Rice CBL and CIPK expression profiles to different abiotic stresses. Click here for file

Additional file 13 Primers used in this study. Click here for file

Additional file 14 Hydroponic system for canola. Click here for file
