# Supplementary material for: Identification and characterization of CBL and CIPK gene families in canola (Brassica napus L.)
Source: BMC Plant Biol. 2014 Jan 7;14:8. doi: 10.1186/1471-2229-14-8 (PMC3890537; doi:10.1186/1471-2229-14-8)
Supplement: Additional file 3 — Analysis of EF-hand motifs in calcium binding proteins of representative species. [file 1471-2229-14-8-S3.pdf]

A

```
AtCaM1          1 ------------------------------------------------------MAD---
AtCaM4          1 ------------------------------------------------------MAD---
OsCaM2          1 ------------------------------------------------------MAD---
OsCaM1-3        1 ------------------------------------------------------MAD---
OsCaM1-1        1 ------------------------------------------------------MAD---
OsCaM1-2        1 ------------------------------------------------------MAD---
AtCaM2          1 ------------------------------------------------------MAD---
AtCaM5          1 ------------------------------------------------------MAD---
AtCaM3          1 ------------------------------------------------------MAD---
AtCaM7          1 ------------------------------------------------------MAD---
OsCaM3          1 ------------------------------------------------------MAD---
MmCaM           1 ------------------------------------------------------MAD---
DrCaM           1 ------------------------------------------------------MAD---
HsCaM           1 ------------------------------------------------------MAD---
DmCaM           1 ------------------------------------------------------MAD---
CeCaM           1 ------------------------------------------------------MAD---
HsCML3          1 ------------------------------------------------------MAD---
SpCaM           1 -----------------------------------------------------MTTR---
ScCaM           1 ------------------------------------------------------MSS---
MmCaM-4         1 ------------------------------------------------------MSH---
AtCML24-TCH2    1 ------------------------------------------------------MSSKNG
AtCBL1          1 -------------------------------------MGCFHSKAAKEF---RGHEDPVK
BnaCBL1         1 -------------------------------------MGCFHSKVAREF---RGHEDPVK
BnaCBL9         1 -------------------------------------MGCLHSMAAREY---PGHENPVK
OsCBL1          1 -------------------------------------MGCFQSTARRPR---PGYEDPVG
BnaCBL2         1 ----------------------MAQCIDGFKHLCTSVLGCFDLDLYKQP---GGLGDPEL
BnaCBL3         1 ----------------------MSQCVDGFKHVCNSLLRCFDIDIGKSS---GGPGDPEL
BnaCBL10        1 MDWTKVSSRSSSLTVGEKVCAVFIPLFAIIDFLFSTVGQCFDCRRRRSPQV-CHHVDLAR
BnaCBL4         1 -------------------------------------MGCSLSKKKKIAIPPPGYEDPDL
consensus       1                                                       mad

                              EF hand 1                      EF hand 2
                     E helix     loop      F helix           E helix
AtCaM1          4 --QLTDEQISEFKEAFSLFDKDGDGCITTKELGTVMRSLGQNPTEAE----LQDMINEVD
AtCaM4          4 --QLTDEQISEFKEAFSLFDKDGDGCITTKELGTVMRSLGQNPTEAE----LQDMINEVD
OsCaM2          4 --QLTDEQIAEFKEAFSLFDKDGDGCITTKELGTVMRSLGQNPTEAE----LQDMINEVD
OsCaM1-3        4 --QLTDDQIAEFKEAFSLFDKDGDGCITTKELGTVMRSLGQNPTEAE----LQDMINEVD
OsCaM1-1        4 --QLTDDQIAEFKEAFSLFDKDGDGCITTKELGTVMRSLGQNPTEAE----LQDMINEVD
OsCaM1-2        4 --QLTDDQIAEFKEAFSLFDKDGDGCITTKELGTVMRSLGQNPTEAE----LQDMINEVD
AtCaM2          4 --QLTDDQISEFKEAFSLFDKDGDGCITTKELGTVMRSLGQNPTEAE----LQDMINEVD
AtCaM5          4 --QLTDDQISEFKEAFSLFDKDGDGCITTKELGTVMRSLGQNPTEAE----LQDMINEVD
AtCaM3          4 --QLTDDQISEFKEAFSLFDKDGDGCITTKELGTVMRSLGQNPTEAE----LQDMINEVD
AtCaM7          4 --QLTDDQISEFKEAFSLFDKDGDGCITTKELGTVMRSLGQNPTEAE----LQDMINEVD
OsCaM3          4 --QLTDDQIAEFKEAFSLFDKDGDGCITTKELGTVMRSLGQNPTEAE----LQDMINEVD
MmCaM           4 --QLTEEQIAEFKEAFSLFDKDGDGTITTKELGTVMRSLGQNPTEAE----LQDMINEVD
DrCaM           4 --QLTEEQIAEFKEAFSLFDKDGDGTITTKELGTVMRSLGQNPTEAE----LQDMINEVD
HsCaM           4 --QLTEEQIAEFKEAFSLFDKDGDGTITTKELGTVMRSLGQNPTEAE----LQDMINEVD
DmCaM           4 --QLTEEQIAEFKEAFSLFDKDGDGTITTKELGTVMRSLGQNPTEAE----LQDMINEVD
CeCaM           4 --QLTEEQIAEFKEAFSLFDKDGDGTITTKELGTVMRSLGQNPTEAE----LQDMINEVD
HsCML3          4 --QLTEEQVTEFKEAFSLFDKDGDGCITTRELGTVMRSLGQNPTEAE----LRDMMSEID
SpCaM           5 --NLTDEQIAEFREAFSLFDRDQDGNITSNELGVVMRSLGQSPTAAE----LQDMINEVD
ScCaM           4 --NLTEEQIAEFKEAFALFDKDNNGSISSSELATVMRSLGLSPSEAE----VNDLMNEID
MmCaM-4         4 --GFTKEEVAEFQAAFNRFDKNKDGHISVEELGDVMKQLGKNLPEKD----LKALISKLD
AtCML24-TCH2    7 VVRSCLGSMDDIKKVFQRFDKNGDGKISVDELKEVIRALSPTASPEE----TVTMMKQFD
AtCBL1         21 LASETAFSVSEVEALFELFKSISSSVVDDGLINKEEFQLALFKSRKRENIFANRIFDMFD
BnaCBL1        21 LASETAFSVSEVEALFELFKSISSSVVDDGLINKEEFQLALFKNRKKENLFANRIFDMFD
BnaCBL9        21 LASETAFSVSEVEALYELFKSISSSVVDDGLINKEEFQLALFKNRKKENLFANRIFDLFD
OsCBL1         21 LASETAFSVSEVEALFELFKSISGSVIDDGLINKEEFRLALFKNKRKENLFANRIFDLFD
BnaCBL2        36 LARDTVFSVSEIEALYELFKKISSAVIDDGLINKEEFQLALFKTNKKESLFADRVFDLFD
BnaCBL3        36 LARDTVFSVSEIEALYELFKKISSAVIDDGLINKEEFQLALFKTNKKESLFADRVFDLFD
BnaCBL10       60 LAHGSPFSVNEVEALYELFKKLSCSIIDDGLIHKEELRLALFQAPYGENLFLDRVFDLFD
BnaCBL4        24 LASVTPFTAAEVEVLYELFKKLSSSIIEDGLIHKEEFQLALLGNRNRNNLFADRIFDVFD
consensus      61   qltdeqi efkeafslFdkdgdg ittkelgtvmrsLgqnpteae    lqdminevD
```

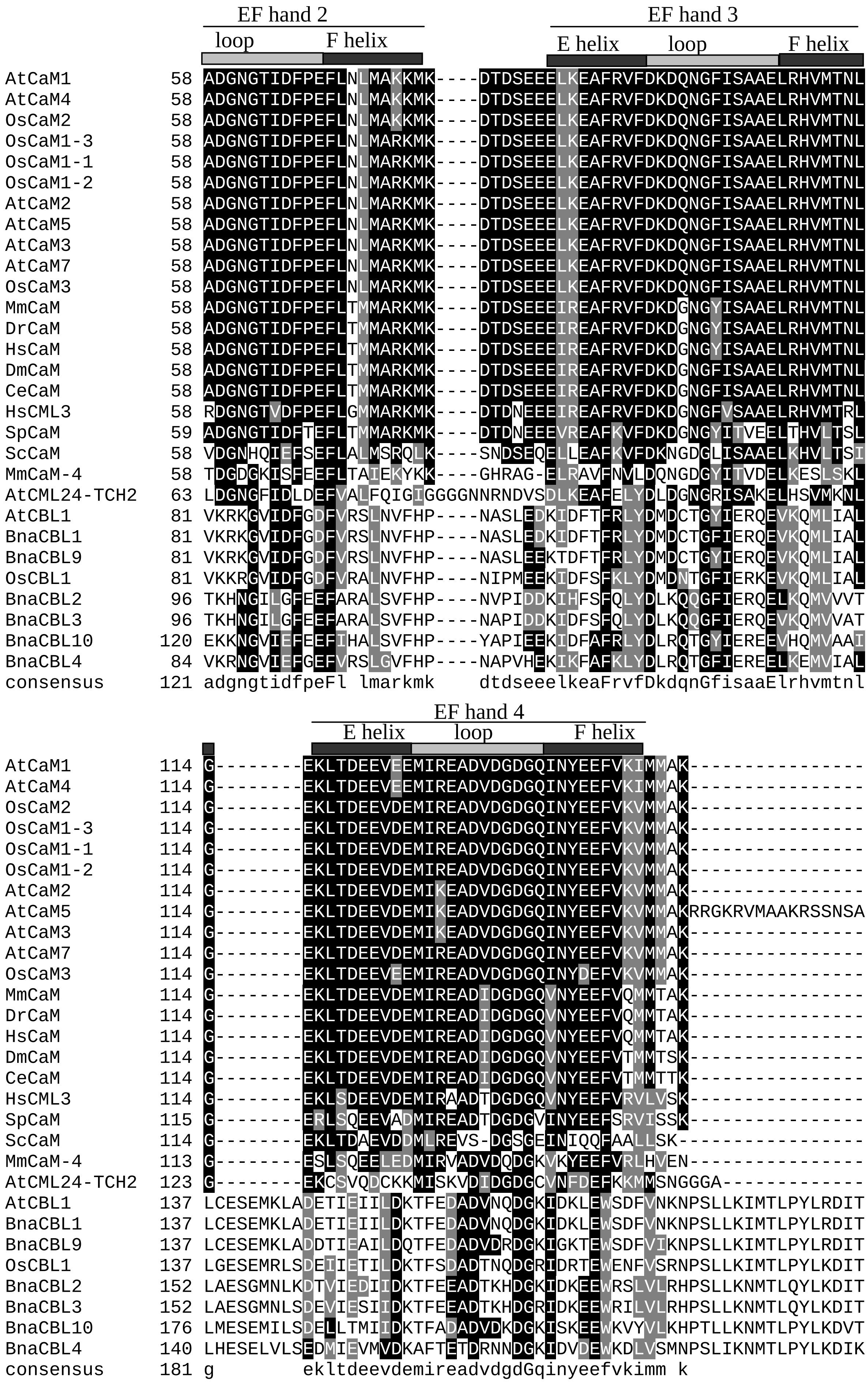
EF hand 2
loop
F helix
EF hand 3
E helix
loop
F helix
AtCaM1 58 ADGNGTIDFPEFLNLMAKKMK----DTDSEEELKEAFRVFDKDQNGFISAAELRHVMTNL
AtCaM4 58 ADGNGTIDFPEFLNLMAKKMK----DTDSEEELKEAFRVFDKDQNGFISAAELRHVMTNL
OsCaM2 58 ADGNGTIDFPEFLNLMAKKMK----DTDSEEELKEAFRVFDKDQNGFISAAELRHVMTNL
OsCaM1-3 58 ADGNGTIDFPEFLNLMARKMK----DTDSEEELKEAFRVFDKDQNGFISAAELRHVMTNL
OsCaM1-1 58 ADGNGTIDFPEFLNLMARKMK----DTDSEEELKEAFRVFDKDQNGFISAAELRHVMTNL
OsCaM1-2 58 ADGNGTIDFPEFLNLMARKMK----DTDSEEELKEAFRVFDKDQNGFISAAELRHVMTNL
AtCaM2 58 ADGNGTIDFPEFLNLMARKMK----DTDSEEELKEAFRVFDKDQNGFISAAELRHVMTNL
AtCaM5 58 ADGNGTIDFPEFLNLMARKMK----DTDSEEELKEAFRVFDKDQNGFISAAELRHVMTNL
AtCaM3 58 ADGNGTIDFPEFLNLMARKMK----DTDSEEELKEAFRVFDKDQNGFISAAELRHVMTNL
AtCaM7 58 ADGNGTIDFPEFLNLMARKMK----DTDSEEELKEAFRVFDKDQNGFISAAELRHVMTNL
OsCaM3 58 ADGNGTIDFPEFLNLMARKMK----DTDSEEELKEAFRVFDKDQNGFISAAELRHVMTNL
MmCaM 58 ADGNGTIDFPEFLTMMARKMK----DTDSEEEIREAFRVFDKDGNGYISAAELRHVMTNL
DrCaM 58 ADGNGTIDFPEFLTMMARKMK----DTDSEEEIREAFRVFDKDGNGYISAAELRHVMTNL
HsCaM 58 ADGNGTIDFPEFLTMMARKMK----DTDSEEEIREAFRVFDKDGNGYISAAELRHVMTNL
DmCaM 58 ADGNGTIDFPEFLTMMARKMK----DTDSEEEIREAFRVFDKDGNGFISAAELRHVMTNL
CeCaM 58 ADGNGTIDFPEFLTMMARKMK----DTDSEEEIREAFRVFDKDGNGFISAAELRHVMTNL
HsCML3 58 RDGNGTVDFPEFLGMMARKMK----DTDNEEEIREAFRVFDKDGNGFVSAAELRHVMTRL
SpCaM 59 ADGNGTIDFTEFLTMMARKMK----DTDNEEEVREAFKVFDKDGNGYITVEELTHVLTSL
ScCaM 58 VDGNHQIEFSEFLALMSRQLK----SNDSEQELLEAFKVFDKNGDGLISAAELKHVLTSI
MmCaM-4 58 TDGDGKISFEEFLTAIEKYKK----GHRAG-ELRAVFNVLDQNGDGYITVDELKESLSKL
AtCML24-TCH2 63 LDGNGFIDLDEFVALFQIGIGGGGNNRNDVSDLKEAFELYDLDGNGRISAKELHSVMKNL
AtCBL1 81 VKRKGVIDFGDFVRSLNVFHP----NASLEDKIDFTFRLYDMDCTGYIERQEVKQMLIAL
BnaCBL1 81 VKRKGVIDFGDFVRSLNVFHP----NASLEDKIDFTFRLYDMDCTGFIERQEVKQMLIAL
BnaCBL9 81 VKRKGVIDFGDFVRSLNVFHP----NASLEEKTDFTFRLYDMDCTGYIERQEVKQMLIAL
OsCBL1 81 VKKRGVIDFGDFVRALNVFHP----NIPMEEKIDFSFKLYDMDNTGFIERKEVKQMLIAL
BnaCBL2 96 TKHNGILGFEEFARALSVFHP----NVPIDDKIHFSFQLYDLKQQGFIERQELKQMVVVT
BnaCBL3 96 TKHNGILGFEEFARALSVFHP----NAPIDDKIDFSFQLYDLKQQGFIERQEVKQMVVAT
BnaCBL10 120 EKKNGVIEFEEFIHALSVFHP----YAPIEEKIDFAFRLYDLRQTGYIEREEVHQMVAAI
BnaCBL4 84 VKRNGVIEFGEFVRSLGVFHP----NAPVHEKIKFAFKLYDLRQTGFIEREELKEMVIAL
consensus 121 adgngtidfpeFl lmarkmk dtdseeelkeaFrvfDkdqnGfisaaElrhvmtnl
EF hand 4
E helix
loop
F helix
AtCaM1 114 G--------EKLTDEEVEEMIREADVDGDGQINYEEFVKIMMAK----------------
AtCaM4 114 G--------EKLTDEEVEEMIREADVDGDGQINYEEFVKIMMAK----------------
OsCaM2 114 G--------EKLTDEEVDEMIREADVDGDGQINYEEFVKVMMAK----------------
OsCaM1-3 114 G--------EKLTDEEVDEMIREADVDGDGQINYEEFVKVMMAK----------------
OsCaM1-1 114 G--------EKLTDEEVDEMIREADVDGDGQINYEEFVKVMMAK----------------
OsCaM1-2 114 G--------EKLTDEEVDEMIREADVDGDGQINYEEFVKVMMAK----------------
AtCaM2 114 G--------EKLTDEEVDEMIKEADVDGDGQINYEEFVKVMMAK----------------
AtCaM5 114 G--------EKLTDEEVDEMIKEADVDGDGQINYEEFVKVMMAKRRGKRVMAAKRSSNSA
AtCaM3 114 G--------EKLTDEEVDEMIKEADVDGDGQINYEEFVKVMMAK----------------
AtCaM7 114 G--------EKLTDEEVDEMIREADVDGDGQINYEEFVKVMMAK----------------
OsCaM3 114 G--------EKLTDEEVEEMIREADVDGDGQINYDEFVKVMMAK----------------
MmCaM 114 G--------EKLTDEEVDEMIREADIDGDGQVNYEEFVQMMTAK----------------
DrCaM 114 G--------EKLTDEEVDEMIREADIDGDGQVNYEEFVQMMTAK----------------
HsCaM 114 G--------EKLTDEEVDEMIREADIDGDGQVNYEEFVQMMTAK----------------
DmCaM 114 G--------EKLTDEEVDEMIREADIDGDGQVNYEEFVTMMTSK----------------
CeCaM 114 G--------EKLTDEEVDEMIREADIDGDGQVNYEEFVTMMTTK----------------
HsCML3 114 G--------EKLSDEEVDEMIRAADTDGDGQVNYEEFVRVLVSK----------------
SpCaM 115 G--------ERLSQEEVADMIREADTDGDGVINYEEFSRVISSK----------------
ScCaM 114 G--------EKLTDAEVDDMLREVS-DGSGEINIQQFAALLSK-----------------
MmCaM-4 113 G--------ESLSQEELEDMIRVADVDQDGKVKYEEFVRLHVEN----------------
AtCML24-TCH2 123 G--------EKCSVQDCKKMISKVDIDGDGCVNFDEFKKMMSNGGGA-------------
AtCBL1 137 LCESEMKLADETIEIILDKTFEDADVNQDGKIDKLEWSDFVNKNPSLLKIMTLPYLRDIT
BnaCBL1 137 LCESEMKLADETIEIILDKTFEDADVNQDGKIDKLEWSDFVNKNPSLLKIMTLPYLRDIT
BnaCBL9 137 LCESEMKLADDTIEAILDQTFEDADVDRDGKIGKTEWSDFVIKNPSLLKIMTLPYLRDIT
OsCBL1 137 LGESEMRLSDEIIETILDKTFSDADTNQDGRIDRTEWENFVSRNPSLLKIMTLPYLKDIT
BnaCBL2 152 LAESGMNLKDTVIEDIIDKTFEEADTKHDGKIDKEEWRSLVLRHPSLLKNMTLQYLKDIT
BnaCBL3 152 LAESGMNLSDEVIESIIDKTFEEADTKHDGRIDKEEWRILVLRHPSLLKNMTLQYLKDIT
BnaCBL10 176 LMESEMILSDELLTMIIDKTFADADVDKDGKISKEEWKVYVLKHPTLLKNMTLPYLKDVT
BnaCBL4 140 LHESELVLSEDMIEVMVDKAFTETDRNNDGKIDVDEWKDLVSMNPSLIKNMTLPYLKDIK
consensus 181 g eekltdeevdemireadvdgdGqinyeefvkimm k

```
AtCaM1                 ----------------------
AtCaM4                 ----------------------
OsCaM2                 ----------------------
OsCaM1-3               ----------------------
OsCaM1-1               ----------------------
OsCaM1-2               ----------------------
AtCaM2                 ----------------------
AtCaM5             166 EYKEKNGRRKSHCRIL------
AtCaM3                 ----------------------
AtCaM7                 ----------------------
OsCaM3                 ----------------------
MmCaM                  ----------------------
DrCaM                  ----------------------
HsCaM                  ----------------------
DmCaM                  ----------------------
CeCaM                  ----------------------
HsCML3                 ----------------------
SpCaM                  ----------------------
ScCaM                  ----------------------
MmCaM-4                ----------------------
AtCML24-TCH2           ----------------------
AtCBL1             197 TTFPSFVFHSEVDEIAT-----
BnaCBL1            197 TTFPSFIFNSEVDEIAT-----
BnaCBL9            197 TTFPSFVFNSEVDEIAT-----
OsCBL1             197 TTFPSFVFNSEVDDLVT-----
BnaCBL2            212 TTFPSFVFHSQVEDT-------
BnaCBL3            212 TTFPSFVFHSQVEDT-------
BnaCBL10           236 TAFPSFIFNTEVED--------
BnaCBL4            200 ATFPSFVLSSEDEELELQNLYF
consensus          241
```

B

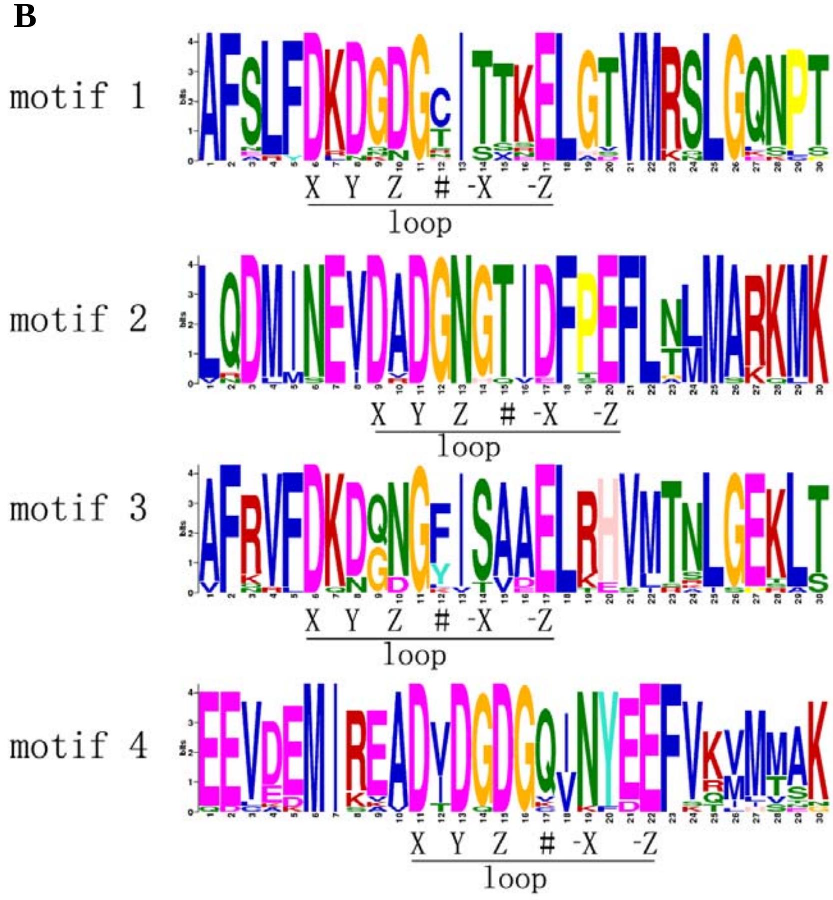

C

species	locus number/ GenBank Acc.#	protein ID	protein name	number of EF-hand motifs
Arabidopsis *(Arabidopsis thaliana)*	At5g37780	AtCAM1	calmodulin 1	4
	At2g41110	AtTCH1/CAM2	calmodulin 2	4
	At3g56800	AtCAM3	calmodulin 3	4
	At1g66410	AtCAM4	calmodulin 4	4
	At2g27030	AtCAM5	calmodulin 5	4
	At3g43810	AtCAM7	calmodulin 7	4
	At5g37770	AtCML24/TCH2	calmodulin-like 24	4
rice (*Oryza sativa* cv. japonica)	LOC_Os01g16240	OsCaM1-3	Calmodulin1-3	4
	LOC_Os01g17190	OsCaM3	Calmodulin3	4
	LOC_Os03g20370	OsCaM1-1	Calmodulin1-1	4
	LOC_Os05g41210	OsCaM2	Calmodulin2	4
	LOC_Os07g48780	OsCaM1-2	Calmodulin1-2	4
baker's yeast (*Saccharomyces cerevisiae*)	NP_009667	ScCaM	Calmodulin	4
fission yeast (*Schizosaccharomyces pombe*)	NP_593340	SpCaM	Calmodulin	4
Caenorhabditis elegans	NP_503386	CeCaM	Calmodulin	4
fruit fly (*Drosophila melanogaster*)	ACT88125	DmCaM	Calmodulin	4
human (*Homo sapiens*)	NP_005176	HsCML3	Calmodulin-like protein 3	4
	BT007607	HsCaM	Calmodulin	4
mouse (*Mus musculus*)	BC145379	MmCaM	Calmodulin	4
	NP_064420	MmCaM4	Calmodulin-4	4
zebrafish (*Danio rerio*)	BC164893	DrCaM	Calmodulin	4

Additional file 3: Analysis of EF-hand motifs in calcium binding proteins of representative species. A. Multiple alignment of calmodulin (CaM), calmodulin-like proteins(CML) with CBLs from representative species. Multiple sequence alignment was performed using the ClustalX1.83 and illustrated by BOXSHADE (http://www.ch.embnet.org/software/BOX_form.html). Identical amino acids are shaded in black, and similar amino acids are shaded in gray. The four EF-hand motifs are indicated by overbars. The regions corresponding to the E helices, the Ca^{2+}-binding loops and the F helices are indicated by the black, gray, and black bars, respectively. The consensus sequences for these protein sequences are indicated beneath the relevant sequences. B. An MEME analysis of the four canonical EF-hand-containing motifs in calmodulin and calmodulin-like proteins of representative species. The 12-residue Ca^{2+}-binding loop within each of the four EF-hand motifs is underlined. Ca^{2+} is bound in a pentagonal bipyramidal geometry with seven sites of

coordination occurring through interactions with six amino acids (positions 1, 3, 5, 7, 9 and 12, alternatively called X, Y, Z, #, -X and -Z). C. Sequences used to construct the EF-hand motif logos in the MEME analysis as presented in B. The numbers of EF-hand motif of each protein sequence were analyzed through SMART (http://smart.embl-heidelberg.de/smart/set_mode.cgi?NORMAL=1).
