# Supplementary material for: Identification and characterization of CBL and CIPK gene families in canola (Brassica napus L.)
Source: BMC Plant Biol. 2014 Jan 7;14:8. doi: 10.1186/1471-2229-14-8 (PMC3890537; doi:10.1186/1471-2229-14-8)
Supplement: Additional file 4 — Multiple alignment of canola CIPK proteins and motif analysis. [file 1471-2229-14-8-S4.pdf]

(A)

```
BnaCIPK11     1 --------MPEIEIVADDGDN-RNN--NALFGKYELGKLLGCGAFAKVFHARDRRSGQSV
BnaCIPK12     1 --------MAELTKETSLPKE-RSSPQALILGRYEMGKLLGHGTFAKVYLARNVKTNESV
BnaCIPK6      1 ----MVGAKPVMKTEENGSDGGANTSTGLLHGRYELGRLLGHGTFAKVYHARNVTTGKSV
BnaCIPK10     1 ----------------------MENKPSVLTDKYEVGRLLGQGTFAKVYYGRSVHTNQSV
BnaCIPK15     1 ----------------------MEKKRTVLMLRYEVGKLLGQGTFAKVYHARNLISGDGV
BnaCIPK25     1 ---------------------MEEERRVVLFGKYEIGRLLGKGTFAKVYHAKHITTGESV
BnaCIPK5      1 ---------------------MEEERR-VLFGKYEMGRLLGKGTFAKVYYGKEITSGESV
BnaCIPK14     1 ---------------------MEEKQRGAVLGKYEVGKLVGCGAFAKVYRGRDTGTGQSV
BnaCIPK7      1 -------------MDSLPQPQNQSSPAKILLGKYELGRRLGSGSFAKVHLARSFETNELV
AtCIPK24      1 -----------------------MTKKMRRVGKYEVGRTIGEGTFAKVKFARNTDTGDNV
BnaCIPK24     1 ----------------MDQKKRITTKKTRKLGKYEVGRTIGEGSFAKVKFARNTDTGENV
BnaCIPK8      1 -------------------------MVVRKVGKYELGRTIGEGTFAKVKFAQNTETGESV
BnaCIPK23     1 MASRTTPSRSTPSRSTPSSGSSSSSISRTRVGKYELGRTLGEGTFAKVKFARNVDKGESV
BnaCIPK9      1 ---------------MIGSRKRTTPASRTRVGNYEMGRTLGEGSFAKVKYARNTVTGDIA
BnaCIPK26     1 --------------------MNR-PKVQRRVGKYEVGKTIGQGSFAKVRYAKNTETGESV
BnaCIPK3      1 --------------------MNRRQQVKRRVGKYEVGRTIGEGTFAKVKFARNSETGEPV
BnaCIPK1      1 ----------------MVRKHEEEVRKEMRLGKYELGRTLGEGNFGKVKFAKDTVSGQPF
BnaCIPK17     1 -----------------------MVTKGMRVGKYELGRTLGEGNSAKVKLATDTVSGQSF
consensus     1                                . ...**.*...* *...**...... .....

BnaCIPK11    50 AVKILNKKKLLANPALANNIKREISIMRRLS-HPNIVGLHEVMATKTKIFFAMEFVKGGE
BnaCIPK12    52 AIKVIDKEKILKG-GLIAHIKREISILRRVR-HPNIVQLFEVMATKSKIYFVMEYVRGGE
BnaCIPK6     57 ALKVVAKEKVIKG-GMEEQIKREISVMKMVK-HPNIVELHEVMASKTKIYFAMELVRGGE
BnaCIPK10    39 AIKMIDKDKVMKVG-LMEQIKREISVMRIAK-HPNVVELYEVMATKTRIYFVMEYCKGGE
BnaCIPK15    39 AVKVIDKDRILKVGGMTDQIKREISVMRLLS-HPNIVHLHEVMATKSKIYFVMEHVKGGE
BnaCIPK25    40 AIKIINKDHVMKRPGMMDQIKREISIMRLVR-HPNIVELKEVMATKTKIFFVMEFVRGGE
BnaCIPK5     39 AIKVINKDHVLKRQGMMEQIKREISIMKLVR-HPNIVELKEVMATKTKIFFVMEFVRGGE
BnaCIPK14    40 AIKVVSKQRLNKGG---LNVKREIAIMHRLR-HPYIVRLSEVLATKSKIFFVMEFAKGGE
BnaCIPK7     48 AIKIIDKKKTIDSN-MEPRIIREIDAMRRLRDHPNILKIHEVMATKSKIYIVMELASGGE
AtCIPK24     38 AIKIMAKSTILKNR-MVDQIKREISIMKIVR-HPNIVRLYEVLASPSKIYIVLEFVTGGE
BnaCIPK24    45 AIKIMAKSTILKNK-MADQIKREISIMKIVR-HPNIVRLYEVLASPSKIYIVLEFVTGGE
BnaCIPK8     36 AMKIVDRNTILKRK-MVDQIKREISIMKLVR-HPCVVRLYEVLASRTKIYIILEYITGGE
BnaCIPK23    61 AIKVIDKEKVLKNK-MIAQIKREISTMKLIK-HPNVIRMIEVMASKTKIYFVLELVTGGE
BnaCIPK9     46 AIKILDRDKVLRHK-MVEQLKREISTMKLIK-HPNVVEIIEVMASKTKIYIVLELVNGGE
BnaCIPK26    40 ALKILDKEKVLKNK-MSEQIRREISTMKLIN-HPNVVRLYEVLASKTKIYIVLEFGVGGE
BnaCIPK3     41 ALKILDKEKVLKHK-MSEQIRREIATMKLIK-HPNVVQLYEVMASKTKIFIILEYVTGGE
BnaCIPK1     45 AVKIIDKSRISHLN-FSLQIKREIRTLKMVK-HPNIVRLHEVLASKTKIYMVMECVMGGE
BnaCIPK17    38 AVKIIDKSSIKRLN-VSFQIKREIRTLKVLK-HPNIVRLHEVLASKTKIYMVLECVTGGD
consensus    61 *.*.........    . ....***....... **..... **.*....*....*.. **.

                                                         Activation loop
BnaCIPK11   109 LFNKISKHGRLSEDLSRRYFQQLISAVGYCHARGVYHRDLKPENLLIDENGNLKVSDFGL
BnaCIPK12   110 LFNKVAKG-RLKEDVARKYFQQLISAVTFCHARGVYHRDIKPENLLLDENGNLKVSDFGL
BnaCIPK6    115 LFAKVAKG-RLREDAARVYFQQLISAVDFCHSRGVYHRDLKPENLLLDEQGNLKITDFGL
BnaCIPK10    97 LFNKVAK-GKLRDDVAWKYFHQLINAVDFCHSRQVYHRDIKPENLLLDDNENLKVSDFGL
BnaCIPK15    98 LFNQVST-GKLREGVARKYFQQLVRAVHYCHTLGVCHRDLKPENLLLDEHGNLKVSDFGL
BnaCIPK25    99 LFAKVVK-GKLKEDAARRYFQQLISAVDFCHSRGVSHRDLKPENLLVDENGDLKVSDFGL
BnaCIPK5     98 LFDKISKEGKLHEDAARRYFHQLISAVDFCHSRGVSHRDLKPENLLLDENGDLKISDFGL
BnaCIPK14    96 LFAKVSK-GRFSEDLSRRYFHQLISAVGYCHSRGVFHRDLKPENLLLDDKLDLKISDFGL
BnaCIPK7    107 LFSKLLRRGRLPESTARRYFQQLASALQFSHRDGVAHRDVKPQNLLLDKEGNLKVSDFGL
AtCIPK24     96 LFDRIVHKGRLEESESRKYFQQLVDAVAHCHCKGVYHRDLKPENLLLDTNGNLKVSDFGL
BnaCIPK24   103 LFDRIVHKGRLEESEARKYFQQLIDAIAHCHCKGVYHRDLKPENLLLDNNGNLKVSDFGL
BnaCIPK8     94 LFDKIVRNGRLSEAEARKYFHQLIDGVDYCHSKGVYHRDLKPENLLLDSQGNLKISDFGL
BnaCIPK23   119 LFDKISSTGRLKEDEARKYFQQLINAVDYCHSRGVYHRDLKPENLLLDANGTLKVSDFGL
BnaCIPK9    104 LFDKIAQQGRLKEDEARRYVQQLINAVDYCHSRGVYHRDLKPENLLLDANGVLKVSDFGL
BnaCIPK26    98 LFDKIVHDGRLQEDAARKYFQQLINAVDYCHSRGVYHRDLKPENLLLDAQGNLKVSDFGL
BnaCIPK3     99 LFDKIVNDGRMKEDEARRYFQQLVHAVDYCHSRGVYHRDLKPENLLLDAYGNLKISDFGL
BnaCIPK1    103 LFDRIVSKGKLSETEGRKMFQQLIDGISYCHSKGVFHRDLKLENVLLDANGHIKITDFGL
BnaCIPK17    96 LFDRIVSKGKLSETEGRTLFQQLIDGVSYCHNKGVFHRDLKLENVLLDAKGHIKITDFGI
consensus   121 **..... ...... ......**. .....*...*.***.*..*.*.*  ....*..***.
                                                              ▲
```

Activation loop

```
BnaCIPK11 169 SALTDHVRPDGLLHTLCGTPAYVAPEILSKKGYDGAKVDVWSCGIVLFVLAAGFLPFNDP
BnaCIPK12 169 SAVSDQIRQDGLFHTFCGTPAYVAPEVLARKGYDAGKVDIWSCGVVLFVLMAGYLPFHDR
BnaCIPK6  174 SAFAEHLRQDGLLHTTCGTPAYVAPEVILKRGYDGAKADLWSCGVILFVLLAGYLPFQDD
BnaCIPK10 156 SALADCKRQDGLLHTTCGTPAYVAPEVINRKGYDGTKADIWSCGVVLFVLLAGYLPFHDS
BnaCIPK15 157 SALSDNRRQDGLLHTTCGTPAYVAPEVINRNGYDGFKADVWSCGVILFVLLAGYLPFRDS
BnaCIPK25 158 SALPEQLLQDGLLHTQCGTPAYVAPEVLRKKGYDGAKADIWSCGVVLYVLLAGYLPFQEE
BnaCIPK5  158 SALPEQILHDGLLHTQCGTPAYVAPEVLRKKGYDGAKADIWSCGVVLYALLAGCLPFRDE
BnaCIPK14 155 SALAEQIRPDGLLHTLCGTPAYVAPEVLAKKGYDGAKIDVWSCGVILFVLNAGYLPFNDH
BnaCIPK7  167 SALPEHLR-NGLLHTACGTPAYTAPEVISRRGYDGAKADAWSCGVILFVLLVGEVPFDDS
AtCIPK24  156 SALPQEG--VELLRTTCGTPNYVAPEVLSGQGYDGSAADIWSCGVILFVILAGYLPFSET
BnaCIPK24 163 SALPQQG--VELLRTTCGTPNYAAPEVLNGQGYDGSAADIWSCGVILFVIMAGFLPFSET
BnaCIPK8  154 SALPEQG--VTILKTTCGTPNYVAPEVLSHKGYNGAVADVWSCGVILYVLMAGYLPFDEM
BnaCIPK23 179 SALPQQVREDGLLHTTCGTPNYVAPEVINNKGYDGAKADLWSCGVILFVLMAGYLPFEDS
BnaCIPK9  164 SAFSLQVREDGLLHTACGTPNYVAPEVLSDKGYDGAAADVWSCGVILFVLMAGYLPFDEP
BnaCIPK26 158 SALSRQVRGDGLLHTACGTPNYAAPEVLNDQGYDGATADLWSRGVILFVLLAGYLPFDES
BnaCIPK3  159 SALSQQVRDDGLLHTSCGTPNYVAPEVLNDGGYDGATADMWSCGVILYVLLAGYLPFDDS
BnaCIPK1  163 SALPQHFRDDGLLHTTCGSPNYVAPEVLANRGYDGAASDIWSCGVILYVILTGCLPFDDR
BnaCIPK17 156 CALPQHFREDGLLHTTCGSPNYVAPEVLANKGYDGAASDIWSCGVILYVILTGCLPFDDA
consensus 181 .*...... .....*.**.* *.***.. ..**.....*.**.*..*.....*..** .
                                 ◆
```

```
BnaCIPK11 229 NLMNMYKKIYKGTYRCPRWMSQDLKRFISRLLDINPETRITIDEILKDPWFVKGGLKTIK
BnaCIPK12 229 NVMAMYKKIYKGEFRCPRWFSPELTRLCSRLLETNPEKRFTFPQIMENSWFKKG-FKHVK
BnaCIPK6  234 NLVNMYKKIYRGDFKCPGWFSSDARRLVVKLLDPNPNTRITIEKVMDSTWFKKR----SK
BnaCIPK10 216 NLMEMYRKIGKGDFKAPSWFAPEVRRLLCKMLDPNPETRITIAKIKESSWFRKG------
BnaCIPK15 217 NLIDLYKKIGKAEFKFPNWFAPGARRLLKKILDPNPNTRISTEKIMQSSWFRRG------
BnaCIPK25 218 NLMNMYRKIFKAEFDFPPWFSPEARRLISKLLVVDPEKRISIPAITRTPWFRKT------
BnaCIPK5  218 NLMNMYRKIFRADFEFPPWFSPESRRLVSRLLVVDPDRRISVTEIMRTPWLREN------
BnaCIPK14 215 NLMVMYRKIYKGEFRIPKWTSPDLRRLLTRLLDTNPLSRITIEEIIRDPWFRQG------
BnaCIPK7  226 NIASMYRKIQRRDYKFPSWISKQAKSIIYQMLDPNPATRMGIETVMKTTWFKKS------
AtCIPK24  214 DLPGLYRKINAAEFSCPPWFSAEVKFLIHRILDPNPKTRIQIQGIKKDPWFRLN------
BnaCIPK24 221 DLPSLYRKISAAEFSCPQWFSADVKSLIQRILDPNPNTRIQIQGIRKHSWFRIN------
BnaCIPK8  212 DLPTLYSKIDNADFSCPSYFALGAKALIHRILDPNPETRITIAEIRKDEWFAKD------
BnaCIPK23 239 NLTSLYKKIFKAEFTCPPWFSASAKKLIKRILDPKPATRITFAEVIENEWFKKG------
BnaCIPK9  224 NLMTLYKRICKAEFNCPPWFSPGAKNVIKRILDPSPITRISIAELLEDEWFKQG------
BnaCIPK26 218 NLMTLYKKITAAEYTCPPWLSPGAKKLIDRILDPSPVTRISIQEILEDAWFKKN------
BnaCIPK3  219 NLMNLYKKISSGEFNCPPWLSLGAMKLITRILDPNPMTRVTPQEVFEDEWFKKD------
BnaCIPK1  223 NLAVLYQKICKGDPPIPRWLSPGARTMVKRMLDPNPATRITVTGIKASEWFKQD------
BnaCIPK17 216 NLAVLFRNIFKGDPPIPRWLSPGAKTMIKRMLDPNPITRMTIAGIKANDWFNHD------
consensus 241 ... ....* .... .* .......... ..*...* .*...  ..   *...
```

NAF/FISL motif

```
BnaCIPK11 289 FHDEVG-----------LDNGAVKGKEDGGGG-------------GEYEVVKSLNAFDLI
BnaCIPK12 288 FYVEDDKLCNVVDDDDELETGSVESDRSSTVSESDVEFFKPARRVGGLPRPASLNAFDII
BnaCIPK6  290 NEPEVQD----------LDLSVHKFK----------------------EETETLNAFHII
BnaCIPK10 270 LHMKQKK----------MEKRVRETNPLEASAAGPSENGGRVTEENNTDQPTSINAFDII
BnaCIPK15 271 LEEDKDH----------ADAETDDTEPMPLNDD----------CDKAKKQCINLNAFEII
BnaCIPK25 272 FKAPMIA----------AINSPGNEL--------------EEEEDGAAVSPKFFNAFQFI
BnaCIPK5  272 FNPPLAF----------EID---------------------DREETEAVSPKFFNAFEFI
BnaCIPK14 269 YDDRMSK----------FHLE--------------------DSDMKLPANEMRMNAFHII
BnaCIPK7  280 LETSQFN----------------------------------FESEPVLEKSSSITAFDLI
AtCIPK24  268 YVPIRAREE---------EEVNLDDIRAVFDGIEG--SYVAENVERNDEGPLMMNAFEMI
BnaCIPK24 275 YMPTRAKEE---------EEVNLDDVRAVFDGIEG--SYVAENIERRYEGPLMMNAFEMI
BnaCIPK8  266 YTPVQVIDY---------EHVNLDDVYAAFDDPE---ERKEAQVGTGDTGPLTLNAFDLI
BnaCIPK23 293 YKAPKYEN----------ADVSLDDVDAIFDESGESKNLVVERKEEGPKNPVTMNAFELI
BnaCIPK9  278 YKTPSFEQDD--------EDITIDDVDAAFSNSKE--CLVTEKKEK----PESMNAFELI
BnaCIPK26 272 YKRPVFEEK---------EEANLDDVEAVFKDSEE--HHVTEKKEE---QPTSMNAFELI
BnaCIPK3  273 YKPPVFEEK---------DDSNMDDVDAVFKDSEE--HHVTEKKEE---QPAAINAFEII
BnaCIPK1  277 YTPSVPDDD------------DDEEEEVDTDDDSFSVQELGSEEGKGSESPTIINAFQLI
BnaCIPK17 270 YTPSNSDDE------------NDAS----------SFQEDVSEEEKSPDFPTVINAFELI
consensus 301 .                 .   ...                .  .     . ...**..*
```

```
                                NAF/FISL motif
                                                       PPI motif
BnaCIPK11  325 S-FSSGLDLSGLFDGCSNSVGEPERFLSEKSPEKLAEEVEEFAKV-EKLRV-NKK--KEE
BnaCIPK12  348 S-FSQGFDLSGLFD----DDGEGSRFVSGAPVSKIISKLEEIAKV-VSFTV-RKKDCRVS
BnaCIPK6   318 A-LSEGFDLSPLFEEKKKEENREMRFATSRPASSVISSLEEAARVGDKFDV-RKSESRVR
BnaCIPK10  320 A-SSSGFDLTGLFGD--VYDKRESRFTSQKPASVIISKLEEVAQRLKLSIR-KREAGLFK
BnaCIPK15  311 S-LSTGFDLSGLFEK--GEEKEEMRFTSNREASEIMEKLVEVGRELKMNVR-KKEE---G
BnaCIPK25  308 SSMSSGFDLSSLFE---SKRKVRSMFTSRWSAAEIVAKIEGIGKEIGMKVK-RTKDFKVR
BnaCIPK5   301 SSMSSGFDLSNLFQ---SKRKVQSVFTSRFSAAEITEKIETVTKQMDMNVK-TTKDFRVK
BnaCIPK14  299 S-GSPGFNLSGLFS---GKFDRVERFVSGWAAARVVEKLEEMAAAENLTVA-SKEAWGMK
BnaCIPK7   306 S-LSSGLDLSGLFE---SKKKKERRFTAKVSVVEVEEKAKAIGERLGYTVR-KTKK----
AtCIPK24   317 T-LSQGLNLSALFDRRQDFVKRQTRFVSRREPSEIIANIEAVANSMGFKSHTRNFKTRLE
BnaCIPK24  324 T-LSQGLNLSALFDRRQDFVKRQTRFVSRREPSVIIANIEAVATSMGFRAHTRNFKTRLE
BnaCIPK8   314 I-LSQGLNLATLFDRGKESMKHQTRFISHKPANVVLSSMEVVSQSMGFKTHIRNYKMRVE
BnaCIPK23  343 S-TSQGLNLGSLFEKQMGLVKRKTRFTSKCSANDIVTKIEAAAGPLGFDVKKNNYKMKLL
BnaCIPK9   324 S-SSNEFSLENLFEKQAQLVKKETRFTSNRPASEIMSKMEETAKPLGFNVRKDKYKIKMK
BnaCIPK26  318 S-MSRALDLGNLFEEEEEGYKRETRFAATGPANELVQKIEEASKPLGFDIQKKNYKMRLE
BnaCIPK3   319 S-MSRGLNLENLFDPEQE-FKRETRITLRGGANEIIDKIEEAAKPLGFDVQKKNYKMRLE
BnaCIPK1   325 G-MSSFLDLSGFFE-QEDVSERRIRFTSNSSARYLLEKIETAVTEMGFSVQKKHAKLKVK
BnaCIPK17  308 G-MSSFLDLSGLFE-TEKVSDRQIRFTSNRLGIDVLEKIKTTLMEMGFYVQKKHTMLKAI
consensus  361 . .* ...*...*.       .. ..... . . ... ....... ... .. .. . ..

BnaCIPK11  380 FEFEMEGPNGKFTIGIYISRLNDLLVVVEARRRGGEVDCYKEM-----WNDKLRLQLIRV
BnaCIPK12  401 LEGSRQGVKGPLTIAAEIFELTPSLVVVEVKKKGGDRTEYEEF-----CNKELKPKLQTL
BnaCIPK6   376 MEGKLSGRKGKLAVEAEIFAVAPSFVVVEVKKDHGDTLEYNNF-----CSTALRPALKDI
BnaCIPK10  376 LETSKEGRKGKLSMDAEIFQVTPTFHMVEMKKSNGDTLEYQKL-----VKEDLRPALADI
BnaCIPK15  364 WRVKMEGRKAGAGVEAEVFEIAPSFHMVVMKKSGGDTAEYKRL-----MKESIRPALMDI
BnaCIPK25  364 MQGKTEGRKGQISVTAEVFEVAPEVAVVELCKSAGDTLEYNKL-----YEEQVRPALKDI
BnaCIPK5   357 IEGKAEGRKGRLSMTAEVFEVAPEMSVVEFCKSAGDTLEYNRF-----YEE-VRPALNDI
BnaCIPK14  354 IQGEG----GSFAMVVEINQLTDELVMIEVRQRQ---RAGGDI-----WSDTLRPFLVEI
BnaCIPK7   357 ---EVVLGSGRTAIAVEAVELAVGVVVVEVKVVD---REEGDS-----RWSDLITELEVI
AtCIPK24   376 GLSSIKAGQ--LAVVIEIYEVAPSLFMVDVRKAAGETLEYHK------FYKKLCSKLENI
BnaCIPK24  383 GLSSIKAGQ--FAVVIEVYEVAPSLFMVDVRKAAGETLEYHK------FYKKLCEKLENI
BnaCIPK8   373 GLSANKSSH--FSVILEVFKVAPTFLMVDIQNAAGDAEEYLKFVNVLQFYKTFCGKLDDI
BnaCIPK23  402 GEKSGRKGQ--LAVATEVFQVAPSLYMVEMRKSGGDTLEFHK------FYKNLTTGLKDI
BnaCIPK9   383 GDKSGRKGQ--LSVATEVFEVAPSLHVVELRKTGGDTLEFHK------FYKTFSSGLKDV
BnaCIPK26  377 NVNAGRKGN--LKVATEIFQVSPSLHMVEVRKTKGDTLEFHK------FYKKLSTTLNDV
BnaCIPK3   377 NVKAGRKGN--LNVATEIFQVAPSLHMVQVSKSKGDTLEFHK------FYKKLSNSLENV
BnaCIPK1   383 QEETNHKGQVGLLVTAEVFEIKPSLNVVELRKSYGDSCLYRQ----------LCERLLKD
BnaCIPK17  366 QQESNRKGQSGLSLTAEVFEIIPLLNVVELRKTHGDSLLYEQ----------LCDRLSNE
consensus  421      ..... . .  ......... ................      .   .   * ..

BnaCIPK11  435 TDQTPNASI--------------------------
BnaCIPK12  456 TADEVDDPVAVSAVVDETASGVANSPPVCFLPSDTE
BnaCIPK6   431 FWTSTPA----------------------------
BnaCIPK10  431 VWVWQSDKDEQLIPDSNQETEQQQQEEEEPL----
BnaCIPK15  419 VLAWH------------------------------
BnaCIPK25  419 VWSWHGDSNNNNNIIIDDSNINNYISDENSGSDC--
BnaCIPK5   411 VWSWHGDNTAVNQIPDGHGTV--------------
BnaCIPK14  402 AHKPDDTVAGLQTTHYQEGASSLRDVNTTT------
BnaCIPK7   406 VLSWHNDVM--------------------------
AtCIPK24   428 IWRATEGIPKSEILRTITF-----------------
BnaCIPK24  435 IWRATEGMPKPELFRTITF-----------------
BnaCIPK8   431 IWKPPDASVRNRVTKTKSKRR--------------
BnaCIPK23  454 VWKTIDEEKEEGTEGKVSGGTTNGTVAAS-------
BnaCIPK9   435 VWNTDGAAQDHKV----------------------
BnaCIPK26  429 VWKSGESSGLNNK----------------------
BnaCIPK3   429 VWTNNEVKKAES-----------------------
BnaCIPK1   433 VGTSSPEQELVT-----------------------
BnaCIPK17  416 LSTSSHVQKLKV-----------------------
consensus  481 ..
```

(B)

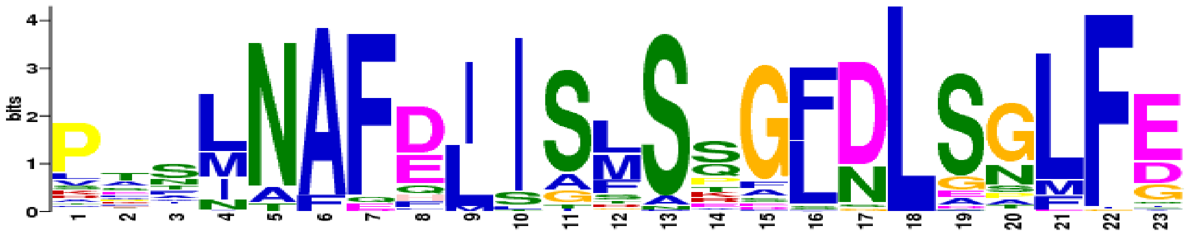

(C)

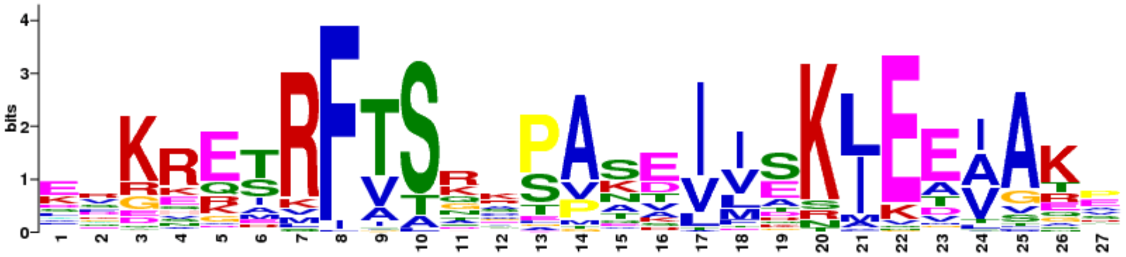

Additional file 4. Multiple alignment of canola CIPK proteins and motif analysis. (A) A multiple alignment of amino acid sequences of 17 canola CIPKs (BnaCIPKs) and Arabidopsis CIPK24(SOS2). Hyphens indicate gaps introduced to maximize the sequence alignment. Identical residues are highlighted in black, and similar residues are highlighted in gray. CIPK proteins consist of a conserved N-terminal kinase domain, and a C-terminal regulatory domain, which is separated from the kinase domain by a variable junction domain. The activation loop is denoted by overbars. The conserved NAF or FISL motif within the rather divergent C-terminal regulatory domain is denoted by dashed lines. The protein–phosphatase interaction (PPI) motif within the C-terminus of these kinases is marked by dots above the sequences. The closed triangle indicates the catalytic base in the kinase domain, and the closed square shows the phosphorylated residue in the activation loop. (B) An MEME analysis of the NAF/FISL motif in 26 Arabidopsis CIPKs, 34 rice CIPKs and 17 canola CIPKs. (C) An MEME analysis of the PPI motif in 26 Arabidopsis CIPKs, 34 rice CIPKs and 17 canola CIPKs.
