# Supplementary material for: Identification and characterization of CBL and CIPK gene families in canola (Brassica napus L.)
Source: BMC Plant Biol. 2014 Jan 7;14:8. doi: 10.1186/1471-2229-14-8 (PMC3890537; doi:10.1186/1471-2229-14-8)
Supplement: Additional file 7 — Multiple alignment of 34 rice CIPK proteins. [file 1471-2229-14-8-S7.pdf]

```
OsCIPK16     1 ------------------------------------------------------------
OsCIPK30     1 ------------------------------------------------------------
OsCIPK27     1 ------------------------------------------------------------
OsCIPK6      1 ------------------------------------------------------------
OsCIPK10     1 ------------------------------------------------------------
OsCIPK2      1 ------------------------------------------------------------
OsCIPK18     1 ------------------------------------------------------------
OsCIPK11     1 ------------------------------------------------------------
OsCIPK26     1 ------------------------------------------------------------
OsCIPK28     1 MAADLQTFGALLKYPKVEGPIRKNASAPASACSSPHRRGDPLDADERRLARGGSRSAEFG
OsCIPK14     1 ------------------------------------------------------------
OsCIPK15     1 ------------------------------------------------------------
OsCIPK20     1 ------------------------------------------------------------
OsCIPK5      1 ------------------------------------------------------------
OsCIPK12     1 ------------------------------------------------------------
OsCIPK19     1 ------------------------------------------------------------
OsCIPK13     1 ------------------------------------------------------------
OsCIPK25     1 ------------------------------------------------------------
OsCIPK29     1 ------------------------------------------------------------
OsCIPK22     1 ------------------------------------------------------------
OsCIPK4      1 ------------------------------------------------------------
OsCIPK7      1 ------------------------------------------------------------
OsCIPK3      1 ------------------------------------------------------------
OsCIPK31     1 ------------------------------------------------------------
OsCIPK32     1 ------------------------------------------------------------
OsCIPK33     1 ------------------------------------------------------------
OsCIPK9      1 ------------------------------------------------------------
OsCIPK23     1 ------------------------------------------------------------
OsCIPK24     1 ------------------------------------------------------------
OsCIPK8      1 ------------------------------------------------------------
OsCIPK1      1 ------------------------------------------------------------
OsCIPK17     1 ------------------------------------------------------------
OsCIPK21     1 ------------------------------------------------------------
OsCIPK34     1 ------------------------------------------------------------
consensus    1
```

```
OsCIPK16    1 ------------------------MARRAREEEADQVERKLVLGRYELGRLLGQGTFAKV
OsCIPK30    1 -----------------------------MAMETTSQDSQVIMGRYKLGRLLGRGTFAKV
OsCIPK27    1 -----------------------------MEGKG------VLEGRYEMGRVLGHGNFGRV
OsCIPK6     1 -----------------------MMAAAAEEGEGKKGGGTVLQGRYEMGRVLGHGNFGRV
OsCIPK10    1 ---------------------------------MVEQKGNILMKRYEIGKLLGQGSFAKV
OsCIPK2     1 ---------------------------------MAEQRGNMLMKKYEMGKLLGQGTFAKV
OsCIPK18    1 -------------------------------MMELEKNGNILLRRYEIGKLLGQGTFAKV
OsCIPK11    1 ----------------------------------MMDGRSILMGRYEVGKQLGQGTFAKV
OsCIPK26    1 ----------------------------------MDDRRTILMDRYEIGRQLGQGNFAKV
OsCIPK28   61 AEALILAEGEQASRRVMQGSILVKTESDSPGNAEICAKRSVLMERYVIGRQLGQGTFGKV
OsCIPK14    1 ----------------------------------MESRGKILMERYELGRLLGKGTFGKV
OsCIPK15    1 ----------------------------------MESRGKILMERYELGRLLGKGTFGKV
OsCIPK20    1 ----------------------------------MPEKGTVVMSRYELGRSLGHGTFSKV
OsCIPK5     1 ----------------------------------MEKKASILMNRYELGRMLGQGTFAKV
OsCIPK12    1 MLMATVSPARREPTPQAVRASPMPSAAAALVRRGGGGSGGTVLGKYELGRVLGQGSFAKV
OsCIPK19    1 --MAATPPSSQ-------HRRPLSSSASAASLAGKPRGGGLLLGRYELGRLLGHGTFAKV
OsCIPK13    1 ---------------------MARMGISKGGSGKEAKKPPLLLGRFEVGKLLGQGNFAKV
OsCIPK25    1 -------------------------MFRSMGTGTGTKPPAMTTERYEFGPLVGEGNFAKV
OsCIPK29    1 --------------MPPSTGSVPPAASTPAAGDEATAAGRVLLGRYELGGLLGRGASAKV
OsCIPK22    1 --------------MPPAGDDESPAATG------DGYSKKVLQGRYELGRVLGQGASSKV
OsCIPK4     1 ---------------------MAMAAMDARKKKSGGGGGEPLLGKYELGRMLGRGTFAKV
OsCIPK7     1 -----------------------MAATKSKAAKKG----APLLGKYELGRLLGRGTFAKV
OsCIPK3     1 --------------------------MYRAKRAALSPKVKRRVGKYELGRTIGEGTFAKV
OsCIPK31    1 --------------------------MYKAKRTAAQ-KVRRCLGKYELGRAIGQGTFAKV
OsCIPK32    1 ---------------------------------MSTTKVKRRVGKYELGRTIGEGTFAKV
OsCIPK33    1 ---------------------------------MSTTKVKRRVGKYELGRTIGEGTFAKV
OsCIPK9     1 -------------------MAEAEAEAAGAGAGAGPARRTTRVGRYELGKTIGEGSFAKV
OsCIPK23    1 ---------------------------------MSVSGGRTRVGRYELGRTLGEGTFAKV
OsCIPK24    1 ----------------------------MGGEEGMAAGRKKRVGRYEVGRTIGQGTFAKV
OsCIPK8     1 ---------------------------------MVGGGALRRVGKYEVGRTIGEGTFAKV
OsCIPK1     1 ---------------------------MVNGEAEAECTRASLLGRYEIGRTLGEGNFGKV
OsCIPK17    1 ---------------------------MVKGG------REALLGGYEMGRTLGEGNFGKV
OsCIPK21    1 ------------------------------------------------------------
OsCIPK34    1 ------------------------------------MGFVESIGRYRVGRTIGAGTFAKV
consensus  61                                         ............. ......
```

```
OsCIPK16    37 YYGRDLRSGESVAIKVIDK-----------ARLRRTEG-MVEQLRREISIMRMV-RHPNV
OsCIPK30    32 YKAYKLATGEAVAIKVFDK-----------EAVQR-SG-TVEQVKREVDVMRRV-HHRHV
OsCIPK27    26 HAARDVRTGRAVAMKVVSK-----------DKVER-AG-MAEQIKREIAVMKMV-SHPSV
OsCIPK6     38 HVARDLRTGRSVAVKVVAK-----------EKVVR-AG-MMEQIKREIAVMKRV-SHPNI
OsCIPK10    28 YHGRNIKNSQSVAIKVIDK-----------EKILKCE--LMDQIRREISVMNLV-RHPCI
OsCIPK2     28 YHARNTETSESVAIKMIDK-----------EKVLKGG--LMDQIKREISVMKLV-RHPNI
OsCIPK18    30 YHGRNIVTSQSVAIKVIDK-----------DKIFKVG--LMDQIKREISVMKLV-RHPNI
OsCIPK11    27 YYARNLTTGQAVAIKMINK-----------DKVMKVG--LMEQIKREISIMRLV-KHPNV
OsCIPK26    27 YYARNLTSGQAVAIKMIDK-----------EKVTRVG--LMVQIKREISIMRLV-KHPNI
OsCIPK28   121 YYARNLSSGQSVAIKMIDK-----------EKILKVG--LMEQIKREISIMRLV-RHPNV
OsCIPK14    27 HYARNLESNQSVAIKMMDK-----------QQVLKVG--LSEQIRREITTMRLV-AHKNI
OsCIPK15    27 HYARNLESNQSVAIKMMDK-----------QQILKVG--LSEQIRREITTMRLV-AHKNI
OsCIPK20    27 YQARSLVSGETVAVKVIDK-----------EKALRAGAGMVDQIEREVAVMRLVGRHPNV
OsCIPK5     27 YHARNLASNQSVAIKVIDK-----------EKVLRVG--MIDQIKREISIMRLV-RHPNI
OsCIPK12    61 YQARHLETDECVAIKVLDK-------------EKAVKGGMVHLVKREINVLRRVR-HPNI
OsCIPK19    52 YQARSADSGEPVAIKVLDK-------------EKAMRHGLVPHIKREIAILRRVR-HPNI
OsCIPK13    40 YHARNVATGEEVAIKVMEK-------------EKIFKSGLTAHIKREIAVLRRVR-HPHI
OsCIPK25    36 YLGRHRATGEEVAIKVMDK-------------EKLVRLGATELIKREIAVMQRLR-HPNV
OsCIPK29    47 YLARDLLTGRDVAIKSFPN---PRHGGGLRGGEEDVLLR-PAPIEREAAILPRLR-HRHV
OsCIPK22    41 YRARDARTGAHVAVKAIRKQQQPHHHPSCRSPEAAAAARRCVEVEREVAALRRVRGHPHV
OsCIPK4     40 YLARAVAG-GEAVAVKVID----------KAEVMGT-AGMAPRVLREVAAMRRLR-HPHV
OsCIPK7     34 YHARSLAPGADPVAVKVLD----------KPDLAAAGAGMATRVLREVAAMRRLR-HPNV
OsCIPK3     35 RFAKNTENDEPVAIKILDK-------------EKVQKHRLVEQIRREICTMKLVK-HPNV
OsCIPK31    34 RFAKNMETGDHVAIKILDK-------------AKVQKHRLVEQIRREICTMKLIQ-HPNV
OsCIPK32    28 KFARDTETGDPVAIKILDK-------------EKVLKHKMVEQIKREISTMKLIK-HPNV
OsCIPK33    28 KFARDTETGDPVAIKILDK-------------EKVLKHKMVEQIKREISTMKLIK-HPNV
OsCIPK9     42 KVARDTRTGDTLAIKVLDR-------------NHVLRHKMVEQIKREISTMKLIK-HPNV
OsCIPK23    28 KFARNADSGENVAIKILDK-------------DKVLKHKMIAQIKREISTMKLIR-HPNV
OsCIPK24    33 KFAVDADTGAAVAMKVLDK-------------DTILNHRMLHQIKREISIMKIVR-HPNI
OsCIPK8     28 KFAQNTESGESVAMKVVDR-------------SSILKHKMADQIKREISIMKLVR-HPNV
OsCIPK1     34 KYARHLATGAHFAIKILDR-------------NKILSLRFDDQIRREIGTLKLLK-HPNV
OsCIPK17    28 KYARHLATGGHFAVKVLDR-------------GRVVSLRAGDQIRREIATLKLLR-HPHV
OsCIPK21     1 ------------------------------------------------------------
OsCIPK34    25 RLAVDADTGATVAVKVIDK-------------RMVIRNNLMYQVKREITAMKLLN-HPNI
consensus  121 . .. . ..  ........                .   ................ ....
```

```
OsCIPK16    84 VGIREVLASRARVFVVMEYARGGELFAKVARG------RLTEEHARRYFQQLVAAVGFCH
OsCIPK30    78 IRLHEVMATRSRIYFVMEYASGGELFTRLSRS-----PRFPEPVARRYFQQLITAVEFCH
OsCIPK27    72 VELHEVMATRTKVYLALELVRGGELFDRIARH-----GRVGEGVARRYFRQLVSAVDFCH
OsCIPK6     84 VELHEVMATRSKIYLALELVRGGELFGRIVRL-----GRVREDAARHYFRQLVSAVDFCH
OsCIPK10    74 VQLYEVMATKTKIYFILEYVKGGELFNKVRR------GRLKEEVARKYFQQLISAIDFCH
OsCIPK2     74 VQLYEVMATKTKIYFVLEHVKGGELFNKVQR------GRLKEDAARKYFQQLICAVDFCH
OsCIPK18    76 VQLYEVMATKSKIYFVLEYVKGGELFNKVAK------GRLKEDAARKYFQQLVSAVDFCH
OsCIPK11    73 LQLFEVIASKSKIYFVLEYAKGGELFNKIAKE-----GKLSEDSARRYFHQLINAVDYCH
OsCIPK26    73 LQLFEVMASKSKIYFVLEYAKGGELFKKISK------GKFSEDVARRYFHQLISGIDYCH
OsCIPK28   167 LQLFEVMATKSNIYFALEYAKGGELFHKMAR------AKLNEESARNYFQQLISAMDYCH
OsCIPK14    73 VQLHEVMATRNKIYFVMEYVKGGELFEKVAKR-----GKLTEVVAHKYFQQLISAVDYCH
OsCIPK15    73 VQLHEVMATRNKIYFVMEYVKGGELFEKVAKR-----GKLTEVVAHKYFQQLISAVDYCH
OsCIPK20    76 VRLHEVMASRSKIYFVMELVRGGELLARLVAGG----GRLGEDAARRYFHQLVAAVDFCH
OsCIPK5     73 VQLHEVMASKSKIYFAMEYVRGGELFSRVARG------RLKEDAARKYFQQLIGAVDFCH
OsCIPK12   107 VQLFEVMASKTKIYFVMEYVRGGELFSRVSKG------RLREDTARRYFQQLVSAVDFCH
OsCIPK19    98 VRLFEVMATKSKIYFVMELVRGGELFGRVAKG------RLKEDTARRYFQQLVSAVGFCH
OsCIPK13    86 VQLYEVMATKLRIYFVMEYVRGGELFARVARG------RLPEADARRYFQQLVSAVAFCH
OsCIPK25    82 VRIHEVMVNKRRICVVMEYVRGGALYRYFRRGPSGGAAGLREHEARRFFQQLVSAVAYCH
OsCIPK29   102 MRLREILATRKKVHFVLDLAAGGELFSLLDASG-----RMTEDLARHYFRQLISAVRYCH
OsCIPK22   101 VALLDVLATRSTVYLVLELASGGSVLSALDSRGGG---HYDEPAARRLFAQLASAVAHAH
OsCIPK4     87 LRLHEVLATRARIYLVMELATGGDLLSRLAALPRR---RLPESAARRVFVQLVDALSYCH
OsCIPK7     83 LRLHEVLATRSKVYLVMELAPGGDLLSRLASLPSR---RLPEHAAQRVFLQLVSALIYCH
OsCIPK3     81 VRLFEVMGSKARIFIVLEYVTGGELFEIIATN-----GRLKEEEARKYFQQLINAVDYCH
OsCIPK31    80 VHLHEVMGSKTRIFIVLEYVMGGELHDIIATS-----GRLKEDEARKYFQQLINAVDYCH
OsCIPK32    74 VRIYEVMGSKTKIYIVLEYVTGGELFDTIVNH-----GRMREDEARRYFQQLINAVDYCH
OsCIPK33    74 VRIYEVMGSKTNIYIVLEYVTGGELFDTIVNH-----GRMREDEARRYFQQLINAVDYCH
OsCIPK9     88 VQLHEVMASKSKIYMVLEYVDGGELFDKIVNS-----GRLGEDEARRYFHQLINAVDYCH
OsCIPK23    74 IRMHEVMASKTKIYIVMELVTGGELFDKIASR-----GRLKEDDARKYFQQLINAVDYCH
OsCIPK24    79 VRLNEVLAGKTKIYIILELITGGELFDKIARQ-----GKLRENEARKYFQQLIDAINYCH
OsCIPK8     74 VRLHEVLASRKKIFIILEFITGGELFDKIIRH-----GRLNEADARRYFQQLIDGVDFCH
OsCIPK1     80 VRLHEVAASKTKIYMVLEYVNGGELFDKIAVKG-----KLSEHEGRRLFQQLIDAVSYCH
OsCIPK17    74 VRLHEVAASKTKIYMVLEFVNGGELFERIAVKG-----KLSEKEGRRLFQQLIDGVSYCH
OsCIPK21     1 ------------------------------------------------------------
OsCIPK34    71 VKIYEVIATKTKICLVMEYVSGGQLSDKLSYLK-----RLDEKEAKKYFYQLIDAVDYCH
consensus  181 .........................  ....      ... .. ......... ......
```

Activation loop

```
OsCIPK16   138 GRGVAHRDLKPENLLLDEEG-----RLKVTDFGLAALPEQLRQDGL----LHTQCGTPAY
OsCIPK30   133 SRGVYHRDLKPENLLLDARG-----DLKVTDFGLSALDGGLRGDGL----LHTTCGTPAY
OsCIPK27   127 GRGVYHRDLKPENLLLDEAG-----NLKVADFGLSALACHARPDGL----LHTACGTPAY
OsCIPK6    139 SRGVYHRDLKPENLLLDEAG-----NLKVVDFGLSALADHARADGL----LHTLCGTPGY
OsCIPK10   128 SRGVYHRDLKPENLLLDENR-----NLKISDFGLSALAECKRQDGL----LHTTCGTPAY
OsCIPK2    128 SRGVYHRDLKPENLLLDENS-----NLKVSDFGLSALADCKRQDGL----LHTTCGTPAY
OsCIPK18   130 SRGVYHRDLKPENLLVDENG-----NLKITDFGLSALAESRRQDGL----LHTTCGTPAY
OsCIPK11   128 SRGVYHRDLKPENLLLDENE-----NLKVSDFGLSALAESKRQDGL----LHTTCGTPAY
OsCIPK26   127 SRGVYHRDLKPENLLLDENE-----SLKVSDFGLSALSESKRHDGL----LHTTCGTPAY
OsCIPK28   221 SRGVYHRDLKPENLLLDENE-----TLKVSDFGLSALAESRRQDGL----LHTACGTPAY
OsCIPK14   128 SRGVYHRDLKPENLLLDENE-----NLKVSDFGLSALSESKRQDGL----LHTTCGTPAY
OsCIPK15   128 SRGVYHRDLKPENLLLDENE-----NLKVSDFGLSALSESKRQDGL----LHTTCGTPAY
OsCIPK20   132 SRGVYHRDLKPENLLVDDDGSGGGGNLKVTDFGLSALSASRRHDGL----LHTTCGTPSY
OsCIPK5    127 SRGVYHRDLKPENLLVDENG-----NLKVSDFGLSAFKECQKQDGL----LHTTCGTPAY
OsCIPK12   161 ARGVFHRDLKPENLLVDENG-----DLKVSDFGLAAGPDQFDPDGL----LHTFCGTPAY
OsCIPK19   152 ARGVFHRDLKPENLLVDEHG-----DLKVSDFGLSAVADQFHPDGL----LHTFCGTPSY
OsCIPK13   140 ARGVFHRDIKPENLLVDDAG-----DLKVSDFGLSAVADGMRRDGL----FHTFCGTPAY
OsCIPK25   142 SRGVFHRDIKLDNLLVDEQG-----NLKVADFGLSALADMERREAH----LQTVCGTPLF
OsCIPK29   157 SRGVYHRDIKPENLLLDDAG-----DLKVADFGLGAVADGALHH--------TLCGTPAY
OsCIPK22   158 SLGVFHRDIKPENLLLDERG-----DLRLTDFGLSAFADADQHLGATDGLAATHCGSPAY
OsCIPK4    144 ARGVAHRDVKPQNVLLDGDG-----NLKVSDFGLAALPDTLRDDGR----LHTACGTPAY
OsCIPK7    140 ARGVSHRDVKPQNVLLDAHG-----NLKVSDFGLAALPDSLRDDGR----LHTACGTPAF
OsCIPK3    136 SRGVYHRDLKLENLLLDASG-----NLKVSDFGLSALTEQVKADGLLH----TTCGTPNY
OsCIPK31   135 SRGVYHRDLKLENLLLDTAG-----NIKVSDFGLSAISEQVKADGLLH----TTCGTPNY
OsCIPK32   129 SRGVYHRDLKPENLLLDSYG-----NLKVSDFGLSALSQQIKDDGLLH----TTCGTPNY
OsCIPK33   129 SRGVYHRDLKPENLLLDSYG-----NLKVSDFGLSALSQQIKDDGLLH----TTCGTPNY
OsCIPK9    143 SRGVYHRDLKPENLLLDSHG-----ALKVSDFGLSAFAPQTKEDGLLH----TACGTPNY
OsCIPK23   129 SRGVYHRDLKPENLLLDASG-----TLKVSDFGLSALSQQVREDGLLH----TTCGTPNY
OsCIPK24   134 SKGVYHRDLKPENLLLDSRG-----NLKVSDFGLSTLAQKG--VGLLH----TTCGTPNY
OsCIPK8    129 SKGVYHRDLKPENLLLDSQG-----NLKISDFGLSAWPAQG--GALLR----TTCGTPNY
OsCIPK1    135 DKGVYHRDLKPENVLVDRRG-----NIKISDFGLSALPQHLGNDGLLH----TTCGSPNY
OsCIPK17   129 DRGVYHRDLKPENVLVDQKG-----NIKISDFGLSALPQHLGNDGLLH----TTCGSPNY
OsCIPK21     1 ------------------------------------------------------------
OsCIPK34   126 RRGVYHRDLKPENLLVDNQG-----NLKVSDFGLSVLKK---PGQFLS----TSCGSPCY
consensus  241 .................. .     ............ .  . ...    ..........
```

Activation loop

```
OsCIPK16   189 VAPEVLRKRGYDGARADLWSCGVVLYVLLCGFLPFQHENYAKMYQKIFKAEYQVPPWVSG
OsCIPK30   184 VAPEVLLKRGYDGAKADIWSCGVILFVLLAGYLPFNETNLVILYRNITESNYRCPPWFSV
OsCIPK27   178 VAPEVLAGNGYDGAKADLWSCGVILYVLLAGALPFQDDNLVCMYRKMRRGDFCCPPWVTT
OsCIPK6    190 AAPEVLRDKGYDGAKADLWSCGVILYVLLAGSLPFPDDNIVTLYRKAQRGDYRCPAWLST
OsCIPK10   179 VAPEVINRKGYDGAKADVWACGVILYVLLAGYLPFQDKNVINMYKKICKAEFKWPSWFSS
OsCIPK2    179 VAPEVINRRGYDGAKADIWSCGVILFVLLAGYLPFHDKNLMDMYKKIGKAEFKCPSWFNT
OsCIPK18   181 VAPEVISRKGYDGVKVDTWSCGVILFVLMAGYLPFQDSNLMEMYRKIGKAEFKCPAWFSS
OsCIPK11   179 VAPEVLSRKGYDGAKADVWSCGVILFVLVAGYLPFHDPNLIEMYRKICRADFRCPRYFSA
OsCIPK26   178 VAPEVLSRRGYDGAKADIWSCGVILFVLVSGYLPFHDTNLIEMYRKIAKAEYKCPRSFSA
OsCIPK28   272 VAPEVLSRKGYSGSKADVWSCGVILFVLVANYLPFHDRNIIQMYRKIAKAEYRCPRHFSA
OsCIPK14   179 VAPEVISKIGYDGAKSDIWSCGVILFVLVAGYLPFQGPNLMEMYRKIQHGEFRCPGWFSR
OsCIPK15   179 VAPEVISKIGYDGAKSDIWSCGVILFVLVAGYLRFQGPNLMEMYRKIQHGEFRCPGWFSR
OsCIPK20   188 VAPEIIGDKGYDGATADVWSCGVILFLLLAGYLPFFDSNLMEMYKKITNGEFKVPDWFTP
OsCIPK5    178 VAPEIINKRGYDGAKADIWSCGVILFVLLAGYLPFHDSNLMEMYRKISKGDVKFPQWFTT
OsCIPK12   212 VAPEVLRRRGYDGAKADIWSCGVILFALMAGYLPFHDHNIMVLYRKIYNGEFRCPRWFSK
OsCIPK19   203 VAPEVLARRGYDGAKADIWSCGIILFVLMAGYLPFHDQNLMAMYRKIYRGEFRCPRWFSK
OsCIPK13   191 VAPEVLSRRGYDAAGADLWSCGVVLFVLMAGYLPFQDRNLAGMYRKIHKGDFRCPKWFSP
OsCIPK25   193 LAPEVFKRRGYDGAKADVWACGVVLYVLLTGRKPFPDEHVSRLYRLIGQNQFQCPPSFSP
OsCIPK29   204 VAPEILSRKGYNPAKVDIWSCGVVLFVLAAGYLPFNDASLVNMYRKIYAGKFRCPAWFSP
OsCIPK22   213 VAPEILLKRRYDASKADVWSCGVVLFVLTAGYLPFNDGNLMAMYRKICAAKFRCPKWCSQ
OsCIPK4    195 AAPEVLRRRAYDGAKADAWSCGVILFVLLAGHLPFDDSNIADMCRKAHRREYELPRWVSQ
OsCIPK7    191 AAPEVLRRKAYDGAKADAWSCGVILFVLLAGHLPFDDSNIADMCRKAHRREYALPRWVSQ
OsCIPK3    187 VAPEVIEDRGYDGAAADIWSCGVILYVLLAGFLPFEDDNIIALYKKISEAQFTCPSWFST
OsCIPK31   186 VAPEVIEDKGYDGALADLWSCGVILFVLLAGYLPFEDENIVSLYNKISGAQFTCPSWFSA
OsCIPK32   180 VAPEVLEDQGYDGAMADLWSCGVILFVLLAGYLPFEDSNLMTLYKKISNAEFTFPPWTSF
OsCIPK33   180 VAPEVLEDQGYDGAMADLWSCGVILFVLLAGYLPFEDSNLMTLYKKISNAEFTFPPWTSF
OsCIPK9    194 VAPEVLADKGYDGMAADVWSCGIILFVLMAGYLPFDDPNLMTLYKLICKAKVSCPHWFSS
OsCIPK23   180 VAPEVINNKGYDGAKADLWSCGVILFVLMAGYLPFEDSNLMSLYKKIFKADFSCPSWFST
OsCIPK24   183 VAPEVLSNNGYDGSAADVWSCGVILYVLMAGYLPFEEDDLPTLYDKITAGQFSCPYWFSP
OsCIPK8    178 VAPEVLSHKGYDGALADTWSCGVILYVLLAGYLPFDEVDLTTPLREDRECRIFIPSLVS-
OsCIPK1    186 IAPEVLQNRGYDGSLSDIWSCGVILYVMLVGYLPFDDRNLVVLYQKIFKGDTQIPKWLSP
OsCIPK17   180 IAPEVLQNKGYDGSLSDIWSCGVILYVMLIGYLPFDDRNIVVLYQKIFKGDTQIPKWLSH
OsCIPK21     1 ----------------------------MLTGNLPFDDQNTVVLYQKILKGDARIPKWLSP
OsCIPK34   174 VAPEVIQHKSYDGAAADVWSCGVILFELLAGYLPFQDCSLTNLYRRISRAQFVFPQWLSV
consensus  301 ...... ...........................* . ... ..... ......* ...
```

```
OsCIPK16   249 DARRLIVRLLVVDPAKRISIPEIMR-TPWFKKGFVPP-----VPTSPVSPK---------
OsCIPK30   244 EARKLLARLLDPNPKTRITISKIMD-RPWFQQATCPLGDMSLVASAPSVLL---------
OsCIPK27   238 DARKLIKSLLDPNPGTRITVAGLLE-TPWFRKTAPVPRPIIADPAAAPVDT---------
OsCIPK6    250 DARRLIPRLLDPNPTTRISVAQLVE-TPWFKKTS-ISRPVSIELPPAFADP---------
OsCIPK10   239 DIRKLLRRILDPNPATRISVSEIME-DPWFRVGLNSDLLNKTIPTDKVDKVV--------
OsCIPK2    239 DVRRLLLRILDPNPSTRISMDKIME-NPWFRKGLDAKLLRYNLQPK---DAI--------
OsCIPK18   241 DVRKLVSRILDPNPRSRMPITKIME-TYWFKKGLDSKLILKNVETNEPVTAL--------
OsCIPK11   239 ELKDLIHKILDSDPSTRISIPRIKR-STWYRKPVEINAKNSEAATTNSISS---------
OsCIPK26   238 ELKDLLYKILDPDPSTRISIPKIKR-SAWYRKSSDVNALKSKHETGDKVYKG--------
OsCIPK28   332 ELKELLYGILDPDPSTRMSISRIKR-SAWYRKPIAISALNNETGKKSCT-----------
OsCIPK14   239 KLQKLLYKIMDPNPSTRISIQKIKE-STWFRKGPEENRILKERTLNENTTK---------
OsCIPK15   239 KLQKLLYKIMDPNPSTRISIQKIKE-STWFRKGPEENRILKERTLNENTTK---------
OsCIPK20   248 DARSLISRLLDPNPTTRITIDELVK-HPWFKKGHT-----KRPASSNTMK----------
OsCIPK5    238 DVRRLLSRLLDPNPNIRITVEKLVE-HPWFKKGYKPAVMLSQPNESNNLKDV--------
OsCIPK12   272 DFTRLITRLLDANPKTRITVPEIIE-SDWFKKGYKPVKFYIED----------DKLYNLS
OsCIPK19   263 DLSSLLNRILDTNPETRITVKEVME-SRWFQKGFRPVRFYVED----------DQVHSLA
OsCIPK13   251 ELIRLLRGVLVTNPQRRATAEGIME-NEWFKIGFRRFSFRVED----------DRTFTCF
OsCIPK25   253 DLARLVRRLLQPDPDRRITIPEIME-MRWFKRGFKEVTYYIDSNDRLRSLDGLDGEPELY
OsCIPK29   264 ELRCLVRRILDPNPATRIDTEEIIT-HPWFRQ----------------------------
OsCIPK22   273 ELRSLIGRMLDPEPDTRIKIGEIFD-HPWLQQ----------------------------
OsCIPK4    255 PARRLVSRLLDPNPDTRVAVESLAAHHPWFKR----------------------------
OsCIPK7    251 PARRLVSRLLDPNPATRLAVAELAT-HPWFKR----------------------------
OsCIPK3    247 GAKKLITRILDPNPTTRITISQILE-DPWFKKGYKPPVFDEKYETSFDDVDA--------
OsCIPK31   246 EAKRLIARILDPNPATRITTSQVLQ-DQWFKKGYESPVFDDKYYPYFHDVYD--------
OsCIPK32   240 PAKRLLTRILDPNPMTRVTIPEILE-DEWFKKGYKRPEFDEKYDTTLDDVYA--------
OsCIPK33   240 PAKRLLTRILDPNPMTRITIPEILE-DEWFKKGYKRPEFDEKYDTTLDDVDA--------
OsCIPK9    254 GAKKFIKRILDPNPCTRITIAQILE-DDWFKKDYKPPLFEQGEDVSLDDVDA--------
OsCIPK23   240 SAKKLIKKILDPNPSTRITIAELIN-NEWFKKGYQPPRFET-ADVNLDDINS--------
OsCIPK24   243 GATSLIHRILDPNPKTRITIEQIRE-DTWFKKTYVAIKRGEDENVDLDDVQA--------
OsCIPK8        ------------------------------------------------------------
OsCIPK1    246 SARDLLRRILEPNPMKRINIAGIKE-HEWFQKDYTPVVPYDDDD-DNYLDSV--------
OsCIPK17   240 SAQNLLRRILEPNPMKRIDMAGIKS-HEWFQKDYIPVLPYDDDDEDVQFGAR--------
OsCIPK21    34 GAQDILRKILDPNPITRLDITGIRA-HEWFRQDYTPAMPFDDDDDNNISDGN--------
OsCIPK34   234 PQKKIIIRILDPSPITRAKISDIFD-DKWLQDHCNPSARIENDDDCDVIEEAS-------
consensus  361 ...... ....... .....  ...    .....
```

```
OsCIPK16   294 ----------------------------------KWEEDDVLLDGGDSGAMS--------
OsCIPK30   294 ----------------------------------ARKEASQQHDDEEDDGFAREKKKRSN
OsCIPK27   288 ----------------------------------RGNAGDDKDEP---------------
OsCIPK6    299 ----------------------------------APAKEEAEKDE---------------
OsCIPK10   290 ---HVD-------------------------MDSTFGNLSNNINEGKQEAEN--------
OsCIPK2    287 ---PVD-------------------------MSTDFDSFNSAPTLEKKPSN---------
OsCIPK18   292 ---ADVN-----------------------VVFSSMGSSSSKKTEEKQDAGK--------
OsCIPK11   289 ------------------------------GVATTSGSAECSTSEENQGSLS--------
OsCIPK26   289 ---EATTSDTTECSIFEGNRASSRDKVYTNGEATTSDSPECSNSDGKQASLS--------
OsCIPK28   380 ------------------------------SEAPFSGPTICISSERNQEPPN--------
OsCIPK14   289 ------------------------------NVALVLGVRRKKNAHEDVKPMS--------
OsCIPK15   289 ------------------------------NVAPVLGVRRKKNAHEDVKPMS--------
OsCIPK20   292 -------------------------------------LNEEEKPANAAMNMK--------
OsCIPK5    289 ---HTAF--------------------------SADHKDNEGKAKEPASSLK--------
OsCIPK12   321 DDVLNLEPADPVPPPLGLAPPVPPPPQGDDPDGSGSESDSSVVSCPATLSTGES------
OsCIPK19   312 DGDNDMPELEPSEPPPPPPFPPPPPQQDDDGEESGWESDSSVASCPATLSSEERR-----
OsCIPK13   300 ELDDDAAVDAPTSPPDTPRTVDSGDVGAAPTRPRKAGSLTSCDSAPSLLEGRFG------
OsCIPK25   312 DSDTDTIESSSSSESPTPVAGTPRGMHTSVSAPALSELDRMEDSASLPLPLPLP------
OsCIPK29   295 ----------------------------------DASHFAMAQLMQHGHDEEAKF-----
OsCIPK22   304 ----------------------------------DGSSSSFGMIQAASSHSKPEV-----
OsCIPK4    287 ----------------------------------SLSVDSQLDGLLNGEPERA-------
OsCIPK7    282 ----------------------------------SLSLDSQLGSLLGGQPERE-------
OsCIPK3    298 ---------------------------------AFGDSED---RHVKEETEDQ-------
OsCIPK31   297 ---------------------------------AFGDSEE---KHVKEAMEEQ-------
OsCIPK32   291 ---------------------------------VFNDSEE---HHVTEKKEE--------
OsCIPK33   291 ---------------------------------VFNDSEE---HHVTEKKEE--------
OsCIPK9    305 ---------------------------------AFDCSEE---NLVAEKREK--------
OsCIPK23   290 ---------------------------------IFNESGDQ-TQLVVERREER-------
OsCIPK24   294 ---------------------------------VFDNIEDKYVSEQVTHNDGG-------
OsCIPK8        ------------------------------------------------------------
OsCIPK1    296 -----------------------------------LPIKEQIDEAKQEK-----------
OsCIPK17   291 -----------------------------------LPAKEQINDEPGDKN----------
OsCIPK21    85 -----------------------------------LHMTENQ-DIETSPA----------
OsCIPK34   286 -----------------------------TDSDSSHNTEVKETEEMTAET----------
consensus  421
```

```
                                        NAF/FISL motif                            PPI motif
                           - - - - - - - - - - - - - - - -                        ..........
OsCIPK16   312 -----------PRTCNAFQLISSMSSGFDLSGMFES------------EQK-AATVFTSR
OsCIPK30   320 VIMSSPVIDVRPSSMNAFDIIS-RSRGLDLSKMFDA------------EERRSEARFSTR
OsCIPK27   299 -----------PEVLNAFHLIS-LSEGFDLSPLFEHDPAASPGRA---TARAGGTRFATR
OsCIPK6    310 -----------PETLNAFHLIS-LSEGFDLSPLFEGDSAKG--------RRDGGMLFATR
OsCIPK10   314 -----------LTSLNAFDIIS-LSSGFDLSAMFEDENS---------KEESK---FTST
OsCIPK2    310 --------------LNAFDIIS-LSTGLDLSGMFE-ESD---------KKESK---FTST
OsCIPK18   318 -----------LTNLNAFDIIS-LSEGFDLSGLFEETDK---------KKEAR---FTSS
OsCIPK11   311 -----------LPNLNAFDIIS-LSTGFNLSGFFEDTHG---------HQEER---FTTR
OsCIPK26   338 -----------LPNLNAFDIIS-LSTGFDLSNLFEERYG---------RREER---FTTR
OsCIPK28   402 -----------LHNLNAFDIIS-LSTGFDLSGLFGERYG---------RRESL---FTSR
OsCIPK14   311 -----------VTNLNAFEIIS-FSKGFDLSGMFIVKEW---------RNEAR---FTSD
OsCIPK15   311 -----------VTNLNAFEIIS-FSKGFDLSGMFIVKEW---------RNEAR---FTSD
OsCIPK20   307 -----------PASLNAFDIIS-LSQGFDLSGMFCCHGH---------SSRTQDQLFVTG
OsCIPK5    312 -----------PVSLNAFDIIS-LSKGFDLSGLFEND-----------KEQKADSRFMTQ
OsCIPK12   375 --QRVRGSLPRPASLNAFDIIS-FSKGFNLSGLFEE--------------RGNEIRFVSG
OsCIPK19   367 --QRPLGSLTRPASLNAFDIIS-FSKGFDLSGLFEE--------------RGSEVRFISA
OsCIPK13   354 ----LGGSSRRRSSLNAFDIIS-FSPGFDLSGLFDQDDGGGAGAGSIPEQQKHTARFVSA
OsCIPK25   366 ----PRPRMPRPKSLNAFDIIA-SSPSFDLSGLFEE--------------RGERMRFVSG
OsCIPK29   316 --KTEFKEDDMARDMTAFDILA-CSPGSDLSGLFGAEPG-------------KER-VFVG
OsCIPK22   325 --EKWEAELEQAMELNAFDIIG-FASGCDLSGLIGPLPD-------------RVRFVLPG
OsCIPK4    306 ----VAFQAAPPPPLNAFDIIS-MSPGLDLSGLFGEHDKS-----------LREKRFTTT
OsCIPK7    301 ----LAFQAPP--PLNAFDIIS-MSPGLDLSGLFGESKR------------RREKRFVTT
OsCIPK3    315 -----------PTSMNAFELIS-LNQALNLDNLFEAKKE-----------YKRETRFTSQ
OsCIPK31   314 -----------PTLMNAFELIS-LNKGLNLDNFFESDKK-----------YKRETRFTSQ
OsCIPK32   307 -----------PEALNAFELIS-MSAGLNLGNLFDSEQE-----------FKRETRFTSK
OsCIPK33   307 -----------PEALNAFELIS-MSAGLNLGNLFDSEQE-----------FKRETRFTSK
OsCIPK9    321 -----------PESMNAFALIS-RSQGFNLGNLFEKEMMGM---------VKRETSFTSQ
OsCIPK23   309 -----------PSVMNAFELIS-TSQGLNLGTLFEKQSGS----------VKRETRFASR
OsCIPK24   314 -----------PLVVNAFEMIT-LSQGLDLSALFDRQQEF----------VKRQTRFVSR
OsCIPK8       ------------------------------------------------------------
OsCIPK1    310 -----------PTHINAFQLIG-MASALDLSGFFEEEDAS-----------QRKIRFTST
OsCIPK17   306 -----------SHQINAFQLIG-MASSLDLSGFFEDEEVS-----------QRRIRFTST
OsCIPK21    99 -----------ISQINAFQLIG-MSSCLDLSGFFEKEDVS-----------ERKIRFVSN
OsCIPK34   307 -----------DRFINAFQLIA-RCSDLDLSGLFQ---------------EQKTKLASP
consensus  481            .  ........ .. .........                  .   ....
```

PPI motif

```
OsCIPK16   348 APAATVIQKLEAVGRSLGYSATRGKGW-----------KLRLE---ATADGANGRLAVT
OsCIPK30   367 ETTTAIVSKLEEIAEAGRFSFKLKEKG------------RVELE---GSQDGRKGALAIE
OsCIPK27   344 EAASGVVARLEALAMGG---------------------AR-------------------
OsCIPK6    350 EPASGVISRLEGVAARGGGRMRVTKSG------------ARGVRLEGAERGGAKGRLAVA
OsCIPK10   350 NTATTITKKLEDVAKNLRLKFLKKNGG------------LLKME---GSKPGRKGVMSIN
OsCIPK2    342 STASTIISKIEDIAKGLRLKLTKKDGG------------LLKME---GSKPGRKGVMGID
OsCIPK18   354 QSASAIISKLEDVASCSKLTVKKKEGG------------VLKME---GASEGRKGVLAID
OsCIPK11   347 QPVTTVLGKLKELAKRLKLKVKKKDNG------------VLRLA---APKEGKKGFLELD
OsCIPK26   374 QPAAAIFAKLNELARRFKLKIKKKENG------------VLRLV---APKEGIKGLLELD
OsCIPK28   438 KPAAAVLVKLKELAKALNLKVTKTDNG------------VLKLA---TTKEGRKGRLELD
OsCIPK14   347 KSASTIISKLEDVAKALNLRVRKKDNG------------VVKMQ---GRKEGRNGVLQFD
OsCIPK15   347 KSASTIISKLEDVAKALNLRVRKKDNG------------VVKMQ---GRKEGRNGVLQFD
OsCIPK20   346 KPATAIVSRLEEIAETEHFTVKKKQKKRQEEDGM-----AVKLQ---GWKEGRKGQLAID
OsCIPK5    349 KPASAIVSKLEQIAETESFKVKKQDG-------------LVKLQ---GSKEGRKGQLAID
OsCIPK12   418 EPMSDIVKKLEEIAKVKSFTVR--RKD--------------WRVSIEGTREGVKGPLTIG
OsCIPK19   410 EPMQTIITKLEEIAKVKSFFVR--RKD--------------WRVSIEGTREGLKGPLTIG
OsCIPK13   409 APVEVIVATLEAAAAAAGMAVRE-RED--------------GSISMEGTREGEHGALAVA
OsCIPK25   407 APVADIIAKLQEIAGMVSFTAR--TKD--------------CQVSIEATRNGQKGALAIS
OsCIPK29   359 EPAAAVLSRVEEAGKKEGYMVTR-------------------------EGKKGTGPVYVK
OsCIPK22   369 GDSKSVLDKVEKLGREEGLVVRR-------------------------KEEEWCGGVHVE
OsCIPK4    350 ASPEKTLEQLGLAGGKLGYVVVVGKKG--------------VECLPLAGGRLSSGIAAMS
OsCIPK7    342 ASPERTVERLGQAGAKLGYFMVG-KKG--------------VERLPLGG---LSGLVAMS
OsCIPK3    352 CPPKEIITKIEEAAKPLGFDIQKKNY----------------KMRMENLKAGRKGNLNVA
OsCIPK31   351 CPPKEIINRIEEAANLLGFNIQKRNY----------------RMRMENIKEGRKGHLNIA
OsCIPK32   344 CPPKEIVRKIEEAAKPLGFDVQKKNY----------------KLRLEKVKAGRKGNLNVA
OsCIPK33   344 CPPKEIVRKIEEAAKPLGFDVQKKNYKICSPCLTTICMNIPFKLRLEKVKAGRKGNLNVA
OsCIPK9    360 CTPQEIMSKIEEACGPLGFNVRKQNY----------------KMKLKGDKTGRKGYLSVA
OsCIPK23   347 LPANEILSKIEAAAGPMGFNVQKRNY----------------KLKLQGENPGRKGQLAIA
OsCIPK24   352 KPAKTIVATIEVVAETMGLKVHSQNY----------------KLRLEGVSSNRMSPFAVV
OsCIPK8        ------------------------------------------------------------
OsCIPK1    347 HSPKDLFDKIENVVTEMGFQVQRGNSK-----------LKVMKNGRGSKNLRNPSSFLVC
OsCIPK17   343 HPPKDAFDKIESSATELGFQVQRGHSK-----------LKLMRNCKGSK---NPESFMVS
OsCIPK21   136 YSPTSLFEKIESTVTEKGFQVQKNSGK-----------LKVIQVCKEPANPRGHGNLLIS
OsCIPK34   339 HPVQETFDKIKVAAKDVSMAVKRMNSS-----------LVEIQDSKLLP--RSNLDLTLS
consensus  541  .    .. ......  ... ...                          . . .... . .
```

```
OsCIPK16   393 VEALEVAADVAVVEFAHDAGDELEFNKFCAVDVRPGLADIVWAWQGD-------RPAAPD
OsCIPK30   412 AEIFKVAPEVHVVEVRKTGGDSPDFRDFYKQELKPSLGDMVWAWQGG-------DSPPLV
OsCIPK27   363 -----VAPSLLMVDVKKDGGDAMEYRPFFSEELRPALKDIVWSPAAT------------
OsCIPK6    398 ADIFSVAPSVLVVDVKKDGGDTLEYRSFCSEELRPALQDIVWGAAAD-------PTPTAA
OsCIPK10   395 AEIFQITPDFHLVEFTKINGDTLEYQKVK-QEMRPALKDIVWAWQGE-------QPQPQS
OsCIPK2    387 AEIFEVTPNFHLVELKKTNGDTLEYRKVLNQEMRPALKDIVWAWQGE-------QPKQQQ
OsCIPK18   399 AEIFEVTPSFHLVEIKKNNGDTLEYQHLWKEDMKPALKDIVWAWQGE-------RQDQQP
OsCIPK11   392 AEIFEVTPSFLLVELKKTNGDTMEYRKLVKEDIRPALKDIVWVWQGD------EHLNSQS
OsCIPK26   419 AEVFELAPSFHLVEFKKSNGDTIEYQKLMKEDIRPALKDIVWAWQGG------QHQQPEQ
OsCIPK28   483 AEVSEVAP-FLLVELKKTNGDTLEYQRMMKEDIRPSLKDIIWTWQGNSIELPNDELTVQC
OsCIPK14   392 IEIFEVTTSYHIIEMKQTSGDSLEYRQLLEEGIRPALKDIVLAWHGDE------------
OsCIPK15   392 IEIFEVTTSYHIIEMKQTSGDSLEYRQLLEEGIRPALKDIVLA-----------------
OsCIPK20   398 AEIFEVSPSCYVVEVKKTAGDTLEYQAFCNRDLRPSLNDICWTSPAT-------AASEKN
OsCIPK5    393 AEIFEVTPSFFVVEVKKSAGDTLEYEKFCNKGLRPSLRDICWDGQSE-------HPSLAQ
OsCIPK12   462 AEIFELTPSLVVVEVKRKAGDNEEYEDFCNMELKPGMQHLVHQMLPAPNGTPVSEKVERS
OsCIPK19   454 AEIFELTPSLVVVEVKKKAGDKEEYDDFCNRELKPGMQHLVHHMGSVPNIPSDTE-----
OsCIPK13   454 AEIYELTPELVVVEVRRKAGGAAEYEEFFRARLKPSLRELVCDDRPCPEDSGELSRSL--
OsCIPK25   451 AKVFELTRELVMVQVCKKAGDTAEYRRFCDNELKAGLRGLVVDALPPPVEGGGHGGAAAA
OsCIPK29   394 GENGGIVAKVCVFKIADAVSVVEVVKGYGAEAARFWKARLEPAMKPPAAI----------
OsCIPK22   404 ATSGKFTAYVRVNLLPKKILMIEAERVIGSEIPKFWHQLQIGNLLVRK------------
OsCIPK4    396 VEMSEVAPPLLLVELRLEVAAGDVDGGDGEVKGFGWEQLRMELG-DVVRAWHSCEDLCEI
OsCIPK7    384 MEMSEVSPSMMLVELRLE--GGDDGDGDGGAEEFGWEELRAELGDDVVMAWHGCDGGKKD
OsCIPK3    396 TEVFQVAPSLHVVELKKAKGDTLEFQKFYRTLST-QLKDVVWKCDGEVEGNGAAA-----
OsCIPK31   395 TEVFQVAPSLHVVELKKAKGDTLEFQKFYQTLST-QLKDVVWELEDAAEDMS--------
OsCIPK32   388 TEILQVAPSLHMVEVRKAKGDTLEFHKFYKNLSR-TLKDVVWKSDDLQNQLS--------
OsCIPK33   404 TEILQVAPSLHMVEVRKAKGDTLEFHKFYKNLSR-TLKDVVWKSDDLQNQLS--------
OsCIPK9    404 TEVFEVAPSLHMVELRKTGGDTLEFHNFYNNFSS-ELKDIVWKSESDAKAAKKREYKNAE
OsCIPK23   391 TEVFEVTPSLYMVELRKSNGDTLEFHKFYHNISN-GLKDVMWKPESSIIAGDEIQHRRSP
OsCIPK24   396 LQVFEVAPSLFMVDVRKVAGDTLEYHRFYKNLCN-KMESIIWRPIEVSAKSALLRTATC-
OsCIPK8        -----------------------------------------------------------
OsCIPK1    396 TEVVELGPSLYVVELKKSHGDPILYRQLCERLSDELGVCKTEQIQRTESLEDDLESFDSG
OsCIPK17   389 AEVFELGPSVNVVELRKSNGDPALYRQLCERISSDMGARNTEQIFATASLEDDLQNSNAG
OsCIPK21   185 AEVFEINESLYVVELKRSSGDCSLYRQLCASLSEDLGICKRQQLLKKDSMRQDLCRYNSS
OsCIPK34   386 AEVIKVTPAHCVVEVSKSTGDLRSYKEFCRSLSSLLNGGQLSASSSDMECD---------
consensus  601 ...... ... ......  ....... .    ... ......
```

```
OsCIPK16   446 VAAATVECSPA-------------------------------------------------
OsCIPK30   465 PAAGRRPITKRS------------------------------------------------
OsCIPK27       ------------------------------------------------------------
OsCIPK6    451 V-----------------------------------------------------------
OsCIPK10   447 LNEQS-------------------------------------------------------
OsCIPK2    440 QPTC--------------------------------------------------------
OsCIPK18   452 EDHGQP------------------------------------------------------
OsCIPK11   446 ILQGEQQQSPLPPELPQDQLQPSLPQQEKQDMPEPPLLPQVPQEEVQTSIPAEQTKN---
OsCIPK26   473 SMQGMQG----------EQQPSRLPSQQPQG-----------------------------
OsCIPK28   542 SEGEVQKQSRLPSIKNFLDRVTVTYSTSRTSWSWTHGHGNCSKNASKNGIKGEAYNADCV
OsCIPK14       ------------------------------------------------------------
OsCIPK15       ------------------------------------------------------------
OsCIPK20   451 QLPAVSEVSPLSSPRN--------------------------------------------
OsCIPK5    446 SSTLTQSSKSISRHAI--------------------------------------------
OsCIPK12   522 SSLQAPLTLKLIGTEGSMS-----------------------------------------
OsCIPK19       ------------------------------------------------------------
OsCIPK13       ------------------------------------------------------------
OsCIPK25   511 AEAE--------------------------------------------------------
OsCIPK29       ------------------------------------------------------------
OsCIPK22       ------------------------------------------------------------
OsCIPK4    455 ------------------------------------------------------------
OsCIPK7    442 KEGILL------------------------------------------------------
OsCIPK3        ------------------------------------------------------------
OsCIPK31       ------------------------------------------------------------
OsCIPK32       ------------------------------------------------------------
OsCIPK33       ------------------------------------------------------------
OsCIPK9    463 SYDIDSCWRKQRWDAKWICKCKWELGHHNHSCCQYSSASFFVMLEMKIYPPFEAPVILPS
OsCIPK23   450 ------------------------------------------------------------
OsCIPK24       ------------------------------------------------------------
OsCIPK8        ------------------------------------------------------------
OsCIPK1    456 SSLPGF------------------------------------------------------
OsCIPK17   449 TPLFAL------------------------------------------------------
OsCIPK21   245 F-----------------------------------------------------------
OsCIPK34       ------------------------------------------------------------
consensus  661
```

```
OsCIPK16      ----------------------------------------------
OsCIPK30      ----------------------------------------------
OsCIPK27      ----------------------------------------------
OsCIPK6       ----------------------------------------------
OsCIPK10      ----------------------------------------------
OsCIPK2       ----------------------------------------------
OsCIPK18      ----------------------------------------------
OsCIPK11      ----------------------------------------------
OsCIPK26      ----------------------------------------------
OsCIPK28   602 SRRSIEVCAKLLEPDDQMTKLIDRLDRRRCKLIDRFRPKERLEMTD
OsCIPK14      ----------------------------------------------
OsCIPK15      ----------------------------------------------
OsCIPK20      ----------------------------------------------
OsCIPK5       ----------------------------------------------
OsCIPK12      ----------------------------------------------
OsCIPK19      ----------------------------------------------
OsCIPK13      ----------------------------------------------
OsCIPK25      ----------------------------------------------
OsCIPK29      ----------------------------------------------
OsCIPK22      ----------------------------------------------
OsCIPK4       ----------------------------------------------
OsCIPK7       ----------------------------------------------
OsCIPK3       ----------------------------------------------
OsCIPK31      ----------------------------------------------
OsCIPK32      ----------------------------------------------
OsCIPK33      ----------------------------------------------
OsCIPK9    523 FFASSVLSASIIYGHGVRYF--------------------------
OsCIPK23      ----------------------------------------------
OsCIPK24      ----------------------------------------------
OsCIPK8       ----------------------------------------------
OsCIPK1       ----------------------------------------------
OsCIPK17      ----------------------------------------------
OsCIPK21      ----------------------------------------------
OsCIPK34      ----------------------------------------------
consensus  721
```

Additional file 7. A multiple alignment of amino acid sequences of 34 rice CIPKs (OsCIPKs). Hyphens indicate gaps introduced to maximize the sequence alignment. Identical residues are highlighted in black, and similar residues are highlighted in gray. Rice CIPK proteins consist of a conserved N-terminal kinase domain, and a C-terminal regulatory domain, which is separated from the kinase domain by a variable junction domain. The conserved NAF or FISL motif within the rather divergent C-terminal regulatory domain is denoted by dashed lines. The protein–phosphatase interaction (PPI) motif within the C-terminus of these kinases is marked by dots above the sequences.
