# Supplementary material for: Identification and characterization of CBL and CIPK gene families in canola (Brassica napus L.)
Source: BMC Plant Biol. 2014 Jan 7;14:8. doi: 10.1186/1471-2229-14-8 (PMC3890537; doi:10.1186/1471-2229-14-8)
Supplement: Additional file 8 — Phylogenetic analysis of CBL proteins from a variety of species. [file 1471-2229-14-8-S8.doc]

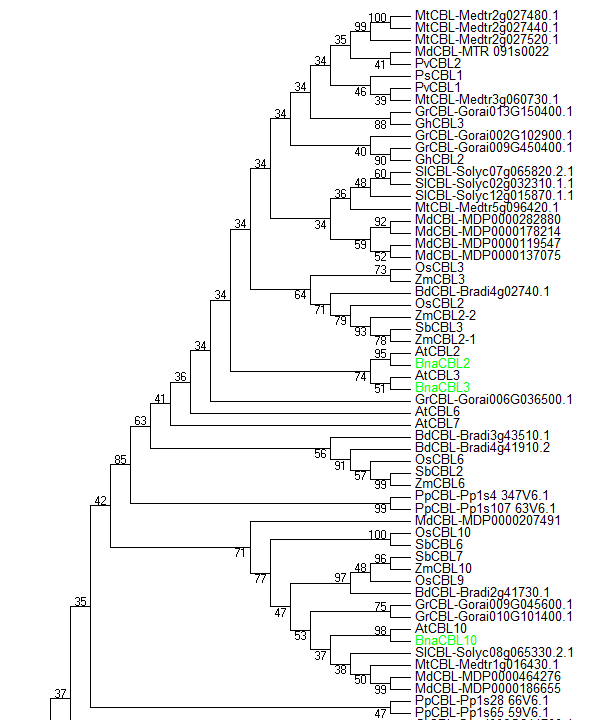


Group IV

Group II


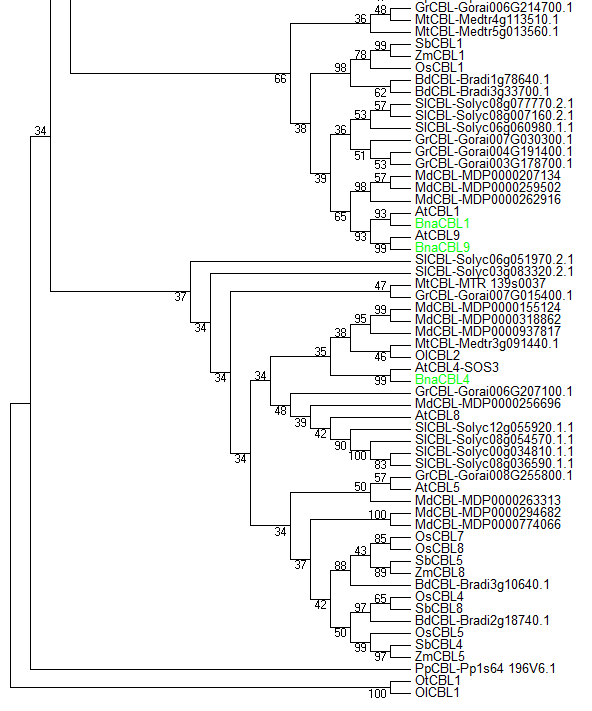


Group III

Group I

Additional file 8. Phylogenetic analysis of calcineurin B-like proteins (CBLs) proteins from a variety of species. The evolutionary relationship was inferred using the maximum parsimony (MP) algorithm implemented in MEGA5.1 program. A CBL from the marine green algae *Ostreococcus tauri* (*Ot*) and *Ostreococcus lucimarinus* (*Ol*), respectively, were used to root the tree. For clarity of presentation, the respective CBL proteins are depicted by a two to three-letter code denoting the species in combination with numbers or locus ID representing the exact CBL from that species. The green highlighted CBL proteins are from canola (*Brassica napus* L.). The analysis involved 117 amino acid sequences. The numbers on the nodes are percentages from a bootstrap analysis of 500 replicates. There were a total of 1047 positions in the final dataset. At, *Arabidopsis thaliana;* Bd*, Brachypodium distachyon;* Bna, *Brassica* *napus*; Gm*, Glycine max;* Gh*, Gossypium hirsutum;* Gr*, Gossypium raimondii;* Md*, Malus domestica;* Mt*, Medicago truncatula;* Ol, *Ostreococcus lucimarinus;* Ot, *Ostreococcus tauri;* Os*, Oryza sativa;* Pp*, Physcomitrella patens;* Ps*, Psium sativum;* Pv, *Phaseolus vulgaris;* Sl*, Solanum lycopersicum;* Sb, [*Sorghum. bicolor*](app:ds:Sorghum bicolor)*;* Zm*, Zea mays.*
