# Supplementary material for: Identification and characterization of CBL and CIPK gene families in canola (Brassica napus L.)
Source: BMC Plant Biol. 2014 Jan 7;14:8. doi: 10.1186/1471-2229-14-8 (PMC3890537; doi:10.1186/1471-2229-14-8)
Supplement: Additional file 9 — Phylogenetic analysis of CIPK genes from a variety of species. [file 1471-2229-14-8-S9.doc]

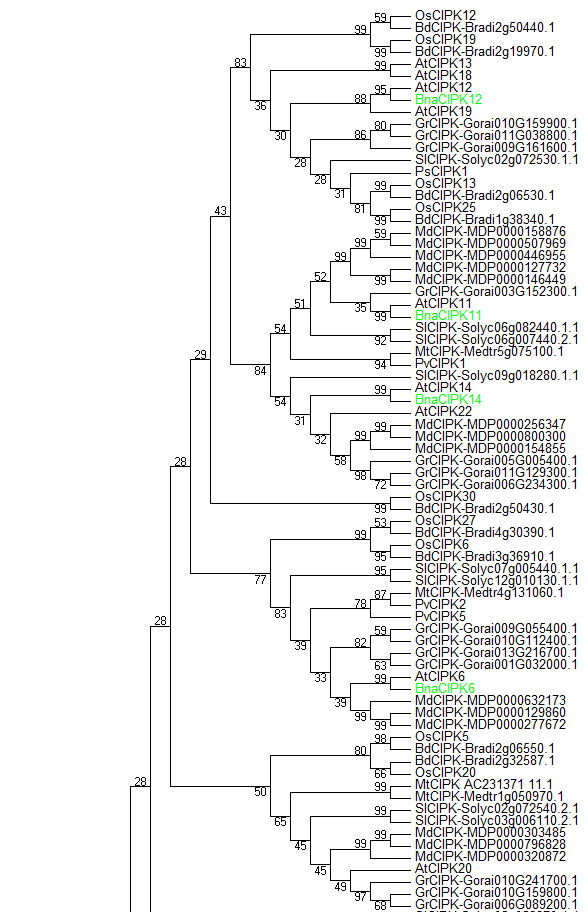


Group

IVb

Group

III


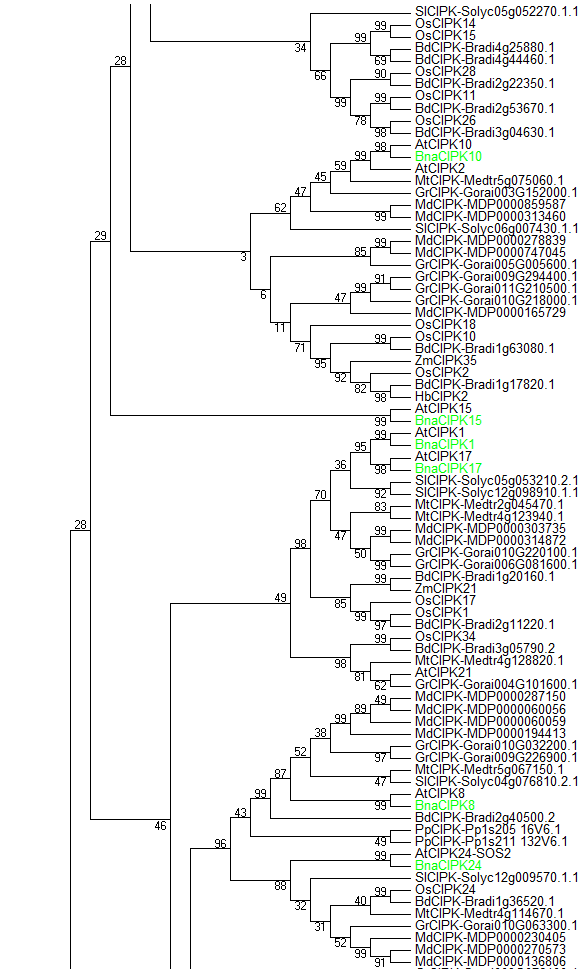


Group

IVa

Group

IIa


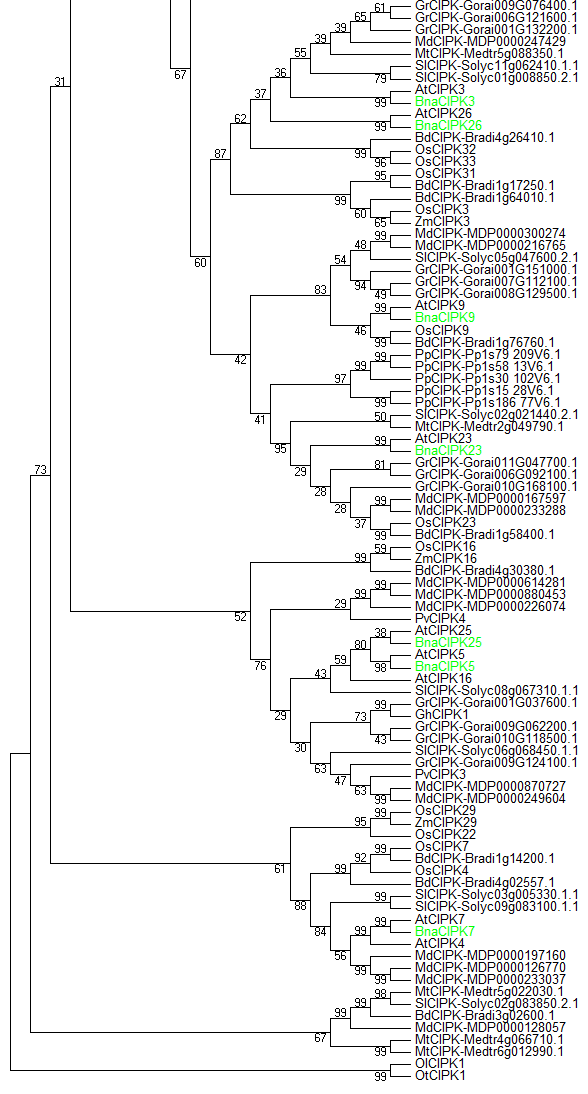


Group

IIb

Group

I

Group

III

Additional file 9. Phylogenetic analysis of CBL-interacting protein kinases (CIPKs) from a variety of species. The evolutionary relationship was inferred using the maximum parsimony (MP) method implemented in MEGA5.1 program. A CIPK from the marine green algae *Ostreococcus tauri* (*Ot*) and *Ostreococcus lucimarinus* (*Ol*) were used to root the tree. For clarity of presentation, the respective CIPK proteins are depicted by a two to three-letter code denoting the species in combination with numbers representing the exact CIPK from that species. The green highlighted CIPK proteins are from canola. The numbers on the nodes are percentages from a bootstrap analysis of 500 replicates. The analysis involved 245 amino acid sequences. There were a total of 1207 positions in the final dataset. At, *Arabidopsis thaliana;* Bd *, Brachypodium distachyon;* Bna, *Brassica* *napus*; Gm*, Glycine max;* Gh*, Gossypium hirsutum;* Gr*, Gossypium raimondii;* Md*, Malus domestica;* Mt*, Medicago truncatula;* Ol, *Ostreococcus lucimarinus;* Ot, *Ostreococcus tauri;* Os*, Oryza sativa;* Pp*, Physcomitrella patens;* Ps*, Psium sativum;* Pv, *Phaseolus vulgaris;* Sl*, Solanum lycopersicum;* Sb, [*Sorghum. bicolor*](app:ds:Sorghum bicolor)*;* Zm*, Zea mays.*
