# Supplementary material for: Identification and characterization of CBL and CIPK gene families in canola (Brassica napus L.)
Source: BMC Plant Biol. 2014 Jan 7;14:8. doi: 10.1186/1471-2229-14-8 (PMC3890537; doi:10.1186/1471-2229-14-8)
Supplement: Additional file 14 — Hydroponic system for canola. [file 1471-2229-14-8-S14.doc]

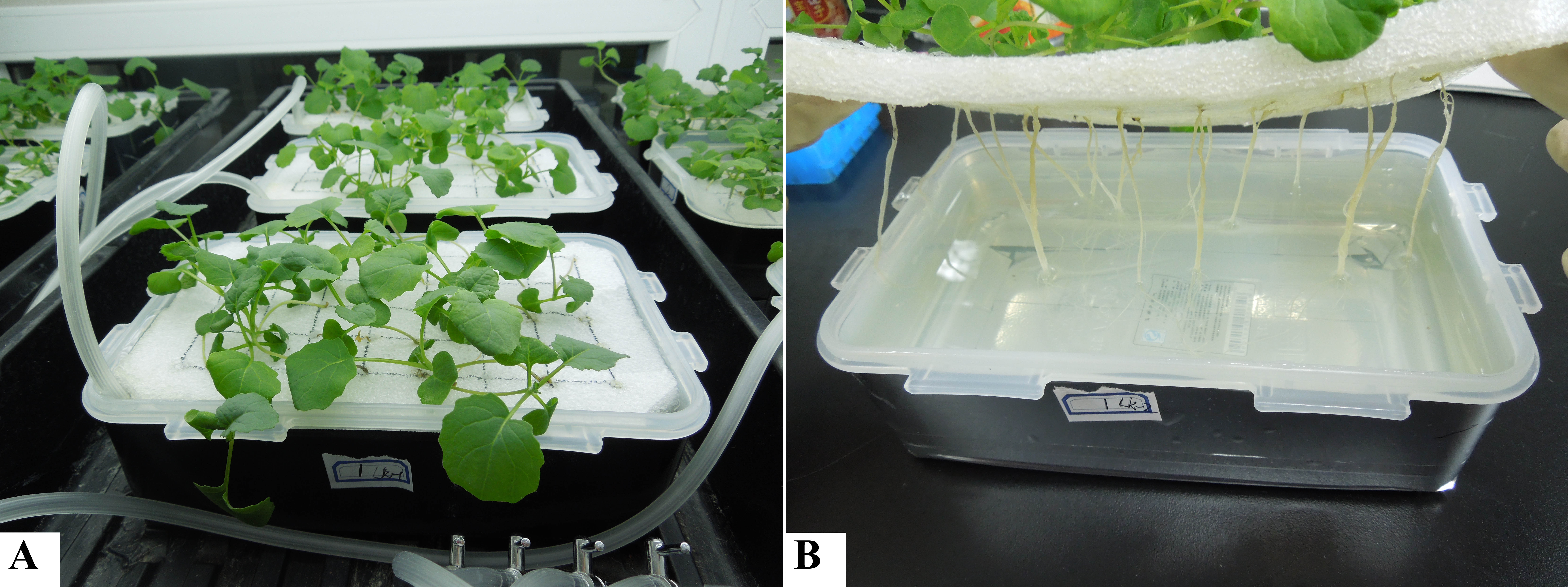


Additional file 14: Hydroponic system for canola. (A) 18 d old canola seedlings after treated with low potasssium (LK, 10 μM K^+^) for 6 h. Plants were aerated constantly. (B) Canola roots from 6 h LK-treated seedlings.
